# Association between common cardiovascular risk factors and clinical phenotype in patients with hypertrophic cardiomyopathy from the European Society of Cardiology (ESC) EurObservational Research Programme (EORP) Cardiomyopathy/Myocarditis registry

**DOI:** 10.1093/ehjqcco/qcac006

**Published:** 2022-02-09

**Authors:** Luis R Lopes, Maria-Angela Losi, Nabeel Sheikh, Cécile Laroche, Philippe Charron, Juan Gimeno, Juan P Kaski, Aldo P Maggioni, Luigi Tavazzi, Eloisa Arbustini, Dulce Brito, Jelena Celutkiene, Albert Hagege, Ales Linhart, Jens Mogensen, José Manuel Garcia-Pinilla, Tomas Ripoll-Vera, Hubert Seggewiss, Eduardo Villacorta, Alida Caforio, Perry M Elliott, The Cardiomyopathy Registry Investigators Group, Christopher Peter Gale, Branko Beleslin, Andrzej Budaj, Ovidiu Chioncel, Nikolaos Dagres, Nicolas Danchin, David Erlinge, Jonathan Emberson, Michael Glikson, Alastair Gray, Meral Kayikcioglu, Aldo Maggioni, Klaudia Vivien Nagy, Aleksandr Nedoshivin, Anna-Sonia Petronio, Jolien Roo Hesselink, Lars Wallentin, Uwe Zeymer, Alida Caforio, Juan Ramon Gimeno Blanes, Philippe Charron, Perry Elliott, Juan Pablo Kaski, Aldo P Maggioni, Luigi Tavazzi, Michal Tendera, S Komissarova, N Chakova, S Niyazova, A Linhart, P Kuchynka, T Palecek, J Podzimkova, M Fikrle, E Nemecek, H Bundgaard, J Tfelt-Hansen, J Theilade, J J Thune, A Axelsson, J Mogensen, F Henriksen, T Hey, S K Nielsen, L Videbaek, S Andreasen, H Arnsted, A Saad, M Ali, J Lommi, T Helio, M S Nieminen, O Dubourg, N Mansencal, M Arslan, V Siam Tsieu, T Damy, A Guellich, S Guendouz, C M Tissot, A Lamine, S Rappeneau, A Hagege, M Desnos, A Bachet, M Hamzaoui, P Charron, R Isnard, L Legrand, C Maupain, E Gandjbakhch, M Kerneis, J-F Pruny, A Bauer, B Pfeiffer, S B Felix, M Dorr, S Kaczmarek, K Lehnert, A-L Pedersen, D Beug, M Bruder, M Böhm, I Kindermann, Y Linicus, C Werner, B Neurath, M Schild-Ungerbuehler, H Seggewiss, B Pfeiffer, A Neugebauer, P McKeown, A Muir, J McOsker, T Jardine, G Divine, P Elliott, M Lorenzini, O Watkinson, E Wicks, H Iqbal, S Mohiddin, C O'Mahony, N Sekri, G Carr-White, T Bueser, R Rajani, L Clack, J Damm, S Jones, R Sanchez-Vidal, M Smith, T Walters, K Wilson, S Rosmini, A Anastasakis, K Ritsatos, V Vlagkouli, T Forster, R Sepp, J Borbas, V Nagy, A Tringer, K Kakonyi, L A Szabo, M Maleki, F Noohi Bezanjani, A Amin, N Naderi, M Parsaee, S Taghavi, B Ghadrdoost, S Jafari, M Khoshavi, C Rapezzi, E Biagini, A Corsini, C Gagliardi, M Graziosi, S Longhi, A Milandri, L Ragni, S Palmieri, I Olivotto, A Arretini, G Castelli, F Cecchi, A Fornaro, B Tomberli, P Spirito, E Devoto, P Della Bella, G Maccabelli, S Sala, F Guarracini, G Peretto, M G Russo, R Calabro, G Pacileo, G Limongelli, D Masarone, V Pazzanese, A Rea, M Rubino, S Tramonte, F Valente, M Caiazza, A Cirillo, G Del Giorno, A Esposito, R Gravino, T Marrazzo, B Trimarco, M-A Losi, C Di Nardo, A Giamundo, F Musella, F Pacelli, A Scatteia, G Canciello, A Caforio, S Iliceto, C Calore, L Leoni, M Perazzolo Marra, I Rigato, G Tarantini, A Schiavo, M Testolina, E Arbustini, A Di Toro, L P Giuliani, A Serio, F Fedele, A Frustaci, M Alfarano, C Chimenti, F Drago, A Baban, L Calò, C Lanzillo, A Martino, M Uguccioni, E Zachara, G Halasz, F Re, G Sinagra, C Carriere, M Merlo, F Ramani, A Kavoliuniene, A Krivickiene, E Tamuleviciute-Prasciene, M Viezelis, J Celutkiene, L Balkeviciene, M Laukyte, E Paleviciute, Y Pinto, A Wilde, F W Asselbergs, A Sammani, J Van Der Heijden, L Van Laake, N De Jonge, R Hassink, J H Kirkels, J Ajuluchukwu, A Olusegun-Joseph, E Ekure, K Mizia-Stec, M Tendera, A Czekaj, A Sikora-Puz, A Skoczynska, M Wybraniec, P Rubis, E Dziewiecka, S Wisniowska-Smialek, Z Bilinska, P Chmielewski, B Foss-Nieradko, E Michalak, M Stepien-Wojno, B Mazek, L Rocha Lopes, A R Almeida, I Cruz, A C Gomes, A R Pereira, D Brito, H Madeira, A R Francisco, M Menezes, O Moldovan, T Oliveira Guimaraes, D Silva, C Ginghina, R Jurcut, A Mursa, B A Popescu, E Apetrei, S Militaru, I Mircea Coman, A Frigy, Z Fogarasi, I Kocsis, I A Szabo, L Fehervari, I Nikitin, E Resnik, M Komissarova, V Lazarev, M Shebzukhova, D Ustyuzhanin, O Blagova, I Alieva, V Kulikova, Y Lutokhina, E Pavlenko, N Varionchik, A D Ristic, P M Seferovic, I Veljic, I Zivkovic, I Milinkovic, A Pavlovic, G Radovanovic, D Simeunovic, M Zdravkovic, M Aleksic, J Djokic, S Hinic, S Klasnja, K Mircetic, L Monserrat, X Fernandez, D Garcia-Giustiniani, J M Larrañaga, M Ortiz-Genga, R Barriales-Villa, C Martinez-Veira, E Veira, A Cequier, J Salazar-Mendiguchia, N Manito, J Gonzalez, F Fernández-Avilés, C Medrano, R Yotti, S Cuenca, M A Espinosa, I Mendez, E Zatarain, R Alvarez, P Garcia Pavia, A Briceno, M Cobo-Marcos, F Dominguez, E De Teresa Galvan, J M García Pinilla, N Abdeselam-Mohamed, M A Lopez-Garrido, L Morcillo Hidalgo, M V Ortega-Jimenez, A Robles Mezcua, A Guijarro-Contreras, D Gomez-Garcia, M Robles-Mezcua, J R Gimeno Blanes, F J Castro, C Munoz Esparza, M Sabater Molina, M Sorli García, D Lopez Cuenca, Palma de Mallorca, T Ripoll-Vera, J Alvarez, J Nunez, Y Gomez, P L Sanchez Fernandez, E Villacorta, C Avila, L Bravo, E Diaz-Pelaez, M Gallego-Delgado, L Garcia-Cuenllas, B Plata, J E Lopez-Haldon, M L Pena Pena, E M Cantero Perez, E Zorio, M A Arnau, J Sanz, E Marques-Sule

**Affiliations:** Serviço de Cardiologia, Hospital Garcia de Orta, Av. Torrado da Silva, Almada 2805-267, Portugal; Institute of Cardiovascular Science, University College London, Gower St, London WC1E 6BT, UK; St. Bartholomew's Hospital, Barts Heart Centre, Barts Health NHS Trust, Whitechapel Rd, London E1 1BB, UK; Department of Advanced Biomedical Sciences, University Federico II, Corso Umberto I, 40, Naples 80138, Italy; Department of Cardiology and Division of Cardiovascular Sciences, Guy's and St. Thomas’ Hospitals and King's College London, Strand, London WC2R 2LS, UK; EORP, European Society of Cardiology, Sophia-Antipolis, France; APHP, Centre de Référence des Maladies Cardiaques Héréditaires, Assistance Publique-Hôpitaux de Paris, ICAN, Hôpital Pitié-Salpêtrière, 47-83 Bd de l'Hôpital, Paris 75013, France; Sorbonne Université, Inserm UMR1166, 15-21 Rue de l'École de Médecine, Paris 75006, France; Hospital Clínico Universitario Virgen de la Arrixaca, Ctra. Madrid-Cartagena, Murcia 30120 El, Spain; Institute of Cardiovascular Science, University College London, Gower St, London WC1E 6BT, UK; Centre for Inherited Cardiovascular Diseases, Great Ormond Street Hospital, London WC1N 3JH, UK; EORP, European Society of Cardiology, Sophia-Antipolis, France; Maria Cecilia Hospital, GVM Care&Research, Via Corriera, 1, Cotignola 48033 RA, Italy; Maria Cecilia Hospital, GVM Care&Research, Via Corriera, 1, Cotignola 48033 RA, Italy; Centre for Inherited Cardiovascular Diseases, Fondazione IRCCS Policlinico San Matteo, Viale Camillo Golgi, Pavia 27100, Italy; Serviço de Cardiologia, Centro Hospitalar Universitário Lisboa Norte, Lisbon 1169-050, Portugal; CCUL, Faculdade de Medicina, Universidade de Lisboa, Av. Prof. Egas Moniz MB, Lisbon 1649-028, Portugal; Clinic of Cardiac and Vascular Diseases, Institute of Clinical Medicine, Faculty of Medicine, Vilnius University, Universiteto g. 3, Vilnius 01513, Lithuania; State Research Institute Centre for Innovative Medicine, Vilnius, Lithuania; Assistance Publique-Hôpitaux de Paris, Hôpital Européen Georges Pompidou, Département de Cardiologie, Paris, France & Université Paris Descartes, Sorbonne Paris Cité, Paris 75006, France; 2nd Department of Internal Cardiovascular Medicine, General University Hospital and First Medical Faculty, Charles University, Opletalova 38, Prague 110 00, Czech Republic; Department of Cardiology, Odense University Hospital, J. B. Winsløws Vej 4, Odense 5000, Denmark; Unidad de Insuficiencia Cardiaca y Cardiopatías Familiares. Servicio de Cardiología. Hospital Universitario Virgen de la Victoria. IBIMA. Málaga and Ciber-Cardiovascular. Instituto de Salud Carlos III. Madrid, Spain; Inherited Cardiovascular Disease Unit Son Llatzer University Hospital & IdISBa, Palma de Mallorca, Spain; Universitätsklinikum Würzburg, Deutsches Zentrum für Herzinsuffizienz (DZHI), Comprehensive Heart Failure Center (CHFC), Am Schwarzenberg 15, Haus 15A, 97078 Wurzburg, Germany; Member of National Centers of expertise for familial cardiopathies (CSUR), Cardiology Department, University Hospital of Salamanca. Institute of Biomedical Research of Salamanca (IBSAL), CIBERCV, Salamanca, Spain; Cardiology, Department of Cardiological, Thoracic and Vascular Sciences and Public Health, University of Padova, Via VIII Febbraio, 2, Padova 35122, Italy; Institute of Cardiovascular Science, University College London, Gower St, London WC1E 6BT, UK; St. Bartholomew's Hospital, Barts Heart Centre, Barts Health NHS Trust, Whitechapel Rd, London E1 1BB, UK

**Keywords:** Cardiovascular risk factors, Hypertension, Diabetes, Obesity, Hypertrophic cardiomyopathy, Genotype

## Abstract

**Aims:**

The interaction between common cardiovascular risk factors (CVRF) and hypertrophic cardiomyopathy (HCM) is poorly studied. We sought to explore the relation between CVRF and the clinical characteristics of patients with HCM enrolled in the EURObservational Research Programme (EORP) Cardiomyopathy registry.

**Methods and results:**

1739 patients with HCM were studied. The relation between hypertension (HT), diabetes (DM), body mass index (BMI), and clinical traits was analysed. Analyses were stratified according to the presence or absence of a pathogenic variant in a sarcomere gene. The prevalence of HT, DM, and obesity (Ob) was 37, 10, and 21%, respectively. HT, DM, and Ob were associated with older age (*P*<0.001), less family history of HCM (HT and DM *P*<0.001), higher New York Heart Association (NYHA) class (*P*<0.001), atrial fibrillation (HT and DM *P*<0.001; Ob p = 0.03) and LV (left ventricular) diastolic dysfunction (HT and Ob *P*<0.001; DM *P* = 0.003). Stroke was more frequent in HT (*P*<0.001) and mutation-positive patients with DM (*P* = 0.02). HT and Ob were associated with higher provocable LV outflow tract gradients (HT *P*<0.001, Ob *P* = 0.036). LV hypertrophy was more severe in Ob (*P* = 0.018). HT and Ob were independently associated with NYHA class (OR 1.419, *P* = 0.017 and OR 1.584, *P* = 0.004, respectively). Other associations, including a higher proportion of females in HT and of systolic dysfunction in HT and Ob, were observed only in mutation-positive patients.

**Conclusion:**

Common CVRF are associated with a more severe HCM phenotype, suggesting a proactive management of CVRF should be promoted. An interaction between genotype and CVRF was observed for some traits.

## Introduction

Hypertrophic cardiomyopathy (HCM) is a common genetic disease associated with heart failure, atrial fibrillation (AF), and sudden cardiac death (SCD).^1^ In many patients, the disease is caused by mutations in genes encoding cardiac sarcomere proteins typified by incomplete penetrance and variable clinical expression, even within families carrying the same causal variant.^1^ Comorbidities such as hypertension (HT) provide a possible explanation for some of this variability, but there are few data on the influence of common cardiovascular risk factors (CVRF) on the HCM phenotype and almost no studies comparing the effect of various CVRFs.^2–5^ We hypothesized that CVRFs are associated with a more severe and/or earlier HCM phenotype.

The EURObservational Research Programme (EORP) Cardiomyopathy/Myocarditis registry is designed to collect prospective clinical data on patients with cardiomyopathies, with the aim of providing insight into disease characteristics and contemporary management of patients with heart muscle diseases in Europe.^6,7^ In this study, our goal was to report the prevalence of CVRF in patients with HCM and to determine their association with the clinical phenotype.

## Methods

### Study population and design

The overall design of the EORP cardiomyopathy registry and inclusion criteria are published elsewhere.^7^ In brief, the registry is a prospective observational multinational survey of consecutive patients presenting to cardiology centres in Europe. Baseline data were collected using a web-based electronic case report form (CRF). Enrollment took place between December 2012 and December 2016. The EORP department of the European Society of Cardiology (ESC) is responsible for study management, data quality control, and statistical analyses.

Inclusion criteria for the adult cardiomyopathies registry were: (i) age at enrollment >18 years, (ii) willingness and ability to give informed consent, (iii) ability to comply with all study requirements, and (iv) documented cardiomyopathy fulfilling standard diagnostic criteria for probands or relatives.

Participating centres managed the approvals of national or regional ethics committees or Institutional Review Boards, according to local regulations. Written informed consent was obtained from all participants before data collection.

The following variables were extracted from the EORP registry database: body mass index (BMI)—categorized in two groups as overweight (BMI≥25 Kg/m^2^) or obese (BMI≥30 Kg/m^2^); HT; and diabetes mellitus (DM). BMI was calculated from height and weight registered in the “physical characteristics” section of the CRF. Although no specific definition for HT or DM was provided, the investigators were expected to follow current guidelines.^8–10^ The relation between HT, DM, and BMI and clinical markers of disease severity was analysed using the following variables: age; sex; family history of HCM; family history of SCD; anaemia, chronic obstructive pulmonary disease, renal insufficiency; cardiac symptoms, ECG, echocardiogram, cardiac magnetic resonance (CMR) imaging, ambulatory ECG monitoring, exercise test, medications, pacemaker and implantable cardioverter–defibrillator (ICD) implantation, and other invasive therapies. Categorical data/classifications (e.g. “diastolic dysfunction”) were registered in the CRF by each recruiting centre investigators, who were expected to follow current guidelines.

In genotyped patients (450, 26%), analyses were stratified according to the presence (“genotype-positive”) or absence of a likely pathogenic/pathogenic variant in one of the eight most prevalent sarcomere genes (*MYBPC3, MYH7, TNNT2, TNNI3, MYL2, MYL3, ACTC1, TPM1*), as recorded by the investigators in the CRF based on the information they had from local testing laboratories.

### Statistical analysis

Univariable analysis was applied to both continuous and categorical variables. Continuous variables were reported as mean ± SD. Among-group comparisons were made using a non-parametric test (Kruskal–Wallis test). Categorical variables were reported as counts and percentages. Among-group comparisons 2×2 were made using a Chi-square test or Fisher's Exact test if any expected cell count was less than five.

Stepwise multivariable logistic regression analyses were performed to establish the relationship of different demographic variables associated with NYHA ≥ 2, with AF, with maximum LV thickness and with LV ejection fraction, including into the model all the candidate variables (variables with *P*<0.10 in univariate). A significance level of 0.05 was required to allow a variable into the model (SLENTRY = 0.05) and a significance level of 0.05 was required for a variable to stay in the model (SLSTAY = 0.05). No interaction was tested. To verify that the models were optimal, Hosmer and Lemeshow Goodness-of-Fit test and percent concordant were calculated. A two-sided *P*<0.05 was considered statistically significant. All analyses were performed using SAS statistical software version 9.4 (SAS Institute, Inc., Cary, NC, USA).

## Results

### Prevalence of hypertension, diabetes, obesity (Tables 1–3 and Supplemental material) and relation to demographic variables and family history

The study cohort comprised 1739 adult patients with HCM; 648 patients (37%) were hypertensive, 176 (10%) were diabetic (type I: 13 and type II: 163), and 1043 (60%) were overweight (*n* = 683, 39%) or obese (*n* = 360 21%). 75 (4%) were both hypertensive and diabetic, 147 (9%) both hypertensive and obese, 13 (1%) diabetic and obese, and 47 (3%) had all three risk factors; 816 patients (47%) had none of these three risk factors reported.

**Table 1:** Comparison between hypertensive and non-hypertensive patients (variables with significant associations; please see supplemental table 1 for all comparisons and stratification according to genotype)

	HTN (*n* = 648)	No HTN (*n* = 1091)	*P*-value
**Demographic and clinical characteristics**			
Age at first evaluation (years)	60.0 (51.0;67.0)	44.0 (32.0;55.0)	<0.001 §
Age at enrolment (years)	63.0 (55.0;71.0)	48.0 (37.0;60.0)	<0.001 §
Gender - Female	277/648 (42.75%)	434/1091 (39.78%)	0.224∼
Body Mass Index (kg/m²)	27.8 (25.1;31.1)	25.7 (23.2;28.4)	<0.001 §
Family history of HCM	156/524 (29.77%)	505/940 (53.72%)	<0.001∼
Family history of SCD	109/621 (17.55%)	241/1041 (23.15%)	0.007∼
Diabetes mellitus II	116/648 (17.90%)	47/1091 (4.31%)	<0.001∼
Hyperlipidaemia/dyslipidaemia	369/648 (56.94%)	266/1091 (24.38%)	<0.001∼
Physical activity (any intensity for > = 2 years)	217/499 (43.49%)	455/867 (52.48%)	0.001∼
Smoking	206/591 (34.86%)	295/995 (29.65%)	0.031∼
Renal impairment	103/648 (15.90%)	55/1091 (5.04%)	<0.001∼
Anaemia	44/641 (6.86%)	36/1076 (3.35%)	<0.001∼
Chronic obstructive pulmonary disease	42/648 (6.48%)	25/1091 (2.29%)	<0.001∼
**Symptoms**			
Age at first symptom (years)	55.0 (45.0;63.0)	39.0 (25.0;51.5)	<0.001 §
Unexplained syncope (suspected arrhythmic cardiogenic+mechanism uncertain)	85/553 (15.37%)	178/900 (19.78%)	0.034∼
Anginal chest pain	245/559 (43.83%)	268/911 (29.42%)	<0.001∼
NYHA functional class > II	107/532 (20.11%)	138/881 (15.66%)	0.032∼
Palpitations	189/559 (33.81%)	358/911 (39.30%)	0.035∼
Ankle oedema	52/559 (9.30%)	55/911 (6.04%)	0.019∼
Paroxysmal nocturnal dyspnoea	37/559 (6.62%)	37/911 (4.06%)	0.029∼
**Arrhythmia and stroke history**			
History of Atrial Fibrillation	205/648 (31.64%)	258/1091 (23.65%)	<0.001∼
History of stroke	57/648 (8.80%)	54/1091 (4.95%)	<0.001∼
Resuscitated ventricular fibrillation/cardiac arrest	8/648 (1.23%)	41/1091 (3.76%)	0.002∼
History of BBB	82/451 (18.18%)	70/607 (11.53%)	0.002∼
**ECG**			
Rhythm : Atrial fibrillation and Atrial flutter	74/628 (11.78%)	73/1056 (6.91%)	<0.001∼
QT interval (ms)	434.0 (400.0;462.0)	425.5 (400.0;454.0)	0.023 §
PR interval (ms)	170.0 (154.0;200.0)	160.0 (144.0;188.0)	<0.001 §
QRS duration (ms)	100.0 (90.0;120.0)	98.0 (88.0;112.0)	0.004 §
QRS axis (degrees)	30.0 (15.0;50.0)	40.0 (20.0;60.0)	<0.001 §
Atrioventricular block : 1st degree	80/628 (12.74%)	97/1053 (9.21%)	0.023∼
Bundle branch block : Incomplete LBBB+LBBB	80/588 (13.61%)	92/983 (9.36%)	0.009∼
ST elevation	113/585 (19.32%)	226/978 (23.11%)	0.078∼
Maximum R in praecordial (mm)	17.0 (11.0;23.0)	15.0 (10.0;22.0)	0.009 §
Maximum S in praecordial (mm)	15.0 (11.0;21.0)	17.0 (12.0;23.0)	<0.001 §
Preexcitation	3/588 (0.51%)	18/983 (1.83%)	0.027∼
**Echocardiogram**			
LV ejection fraction (Simpson's biplane) (%)	60.0 (55.0;69.0)	64.0 (58.0;70.0)	0.020 §
LV posterior wall thickness diastole (mm)	12.0 (10.0;14.0)	11.0 (9.0;13.0)	<0.001 §
Left atrium diameter (mm)	45.0 (40.0;51.0)	43.0 (38.0;49.0)	<0.001 §
Left atrial area (cm²)	27.0 (22.0;33.0)	24.9 (20.0;31.0)	0.005 §
Pattern of LV hypertrophy Asymmetrical septal hypertrophy	391/604 (64.74%)	776/1023 (75.86%)	<0.001∼
Concentric	112/604 (18.54%)	108/1023 (10.56%)	
Apical	67/604 (11.09%)	76/1023 (7.43%)	
Maximum RV wall thickness (mm)	6.0 (4.0;7.0)	5.0 (4.0;7.0)	0.006 §
Diastolic function - normal	67/467 (14.35%)	241/805 (29.94%)	<0.001∼
Left ventricular outflow tract gradient (mmHg)	13.0 (6.0;45.0)	8.4 (4.0;35.0)	0.004 §
Maximum provoked (by any technique) peak left ventricular outflow tract gradient (mmHg)	20.0 (7.0;70.0)	10.6 (5.0;40.0)	<0.001 §
Aortic regurgitation - no	387/578 (66.96%)	785/964 (81.43%)	<0.001†
Mitral regurgitation - no	120/578 (20.76%)	280/964 (29.05%)	<0.001∼
Systolic Pulmonary Artery pressure (mmHg)	34.0 (25.0;40.0)	30.0 (24.0;37.0)	0.005 §
**Cardiac Magnetic Resonance**			
Late gadolinium enhancement	134/197 (68.02%)	300/391 (76.73%)	0.037∼
**Holter**			
Rhythm : atrial fibrillation throughout + paroxysmal atrial fibrillation in sinus rhythm	66/432 (15.28%)	62/730 (8.49%)	<0.001∼
**Exercise test**			
Absolute workload achieved (METS)	7.0 (5.1;8.2)	8.3 (6.0;11.3)	0.002 §
Max VO2 achieved (ml/min/Kg)	17.1 (14.0;20.5)	21.0 (17.5;27.1)	<0.001 §
% of maximum estimated VO2 achieved Bicycle	27.8 (9.7;32.2)	29.3 (12.2;35.6)	0.027 §
% of maximum estimated VO2 achieved Treadmill	31.1 (24.8;37.8)	38.4 (33.1;44.1)	<0.001 §
**Device**			
Cardioverter defibrillator implanted	82/648 (12.65%)	264/1091 (24.20%)	<0.001∼

**Key:** §: Kruskal–Wallis test; †: Exact–Fisher test; ∼: Chi-square test; NC: Not calculable. All continuous variables are presented as Median (Q1; Q3) and categorical variables as *N* and percentage. HCM, hypertrophic cardiomyopathy; SCD, sudden cardiac death; NYHA, New York Heart Association; BBB, bundle branch block; LBBB, left bundle branch block; LVEDD, left ventricular end-diastolic dimension; LVESD, left ventricular end-systolic dimension; LV, left ventricle; RV, right ventricle; VO2, oxygen consumption.

**Table 2:** Comparison between diabetic and non-diabetic patients (variables with significant associations; please see supplemental table 2 for all comparisons and stratification according to genotype)

	Diabetes (*n* = 176)	No diabetes (*n* = 1563)	*P*-value
**Demographics and co-morbidities**			
Age at first evaluation in the centre (years)	60.0 (53.0;69.0)	49.0 (36.0;60.0)	<0.001 §
Age at enrolment	65.0 (57.0;72.0)	54.0 (41.0;64.0)	<0.001 §
Body Mass Index (kg/m²)	28.9 (26.1;31.1)	26.2 (23.5;29.3)	<0.001 §
Family history of HCM	44/146 (30.14%)	617/1318 (46.81%)	<0.001∼
Family history of SCD	24/167 (14.37%)	326/1495 (21.81%)	0.025∼
Hypertension	122/176 (69.32%)	526/1563 (33.65%)	<0.001∼
Hyperlipidaemia/dyslipidaemia	108/176 (61.36%)	527/1563 (33.72%)	<0.001∼
Physical activity	51/139 (36.69%)	621/1227 (50.61%)	0.002∼
Renal impairment	38/176 (21.59%)	120/1563 (7.68%)	<0.001∼
Anaemia	22/172 (12.79%)	58/1545 (3.75%)	<0.001∼
Chronic obstructive pulmonary disease	19/176 (10.80%)	48/1563 (3.07%)	<0.001∼
**Symptoms**			
Age at first symptom (years)	54.0 (42.0;66.0)	43.0 (29.0;55.0)	<0.001 §
NYHA functional class > II	42/154 (27.27%)	203/1259 (16.12%)	<0.001∼
Palpitations	41/158 (25.95%)	506/1312 (38.57%)	0.002∼
**Arrhythmia history**			
History of Atrial Fibrillation	71/176 (40.34%)	392/1563 (25.08%)	<0.001∼
Sustained ventricular tachycardia	5/176 (2.84%)	129/1563 (8.25%)	0.011∼
**ECG**			
Rhythm : Atrial fibrillation and Atrial flutter	35/173 (20.23%)	112/1511 (7.41%)	<0.001∼
PR interval (ms)	180.0 (160.0;203.0)	164.0 (146.0;190.0)	<0.001 §
Atrioventricular block : 1st degree	27/173 (15.61%)	150/1508 (9.95%)	0.022∼
ST elevation	25/162 (15.43%)	314/1401 (22.41%)	0.041∼
Maximum S in praecordial (mm)	13.0 (10.0;19.0)	16.0 (12.0;22.0)	<0.001 §
**Echocardiogram**			
E-wave deceleration time (m/s)	225.0 (190.0;269.0)	200.0 (161.0;242.0)	0.003 §
Mitral A-wave (m/s)	0.8 (0.6;1.0)	0.6 (0.5;0.8)	<0.001 §
Diastolic dysfunction -normal	19/124 (15.32%)	289/1148 (25.17%)	0.003∼
Systolic Pulmonary Artery pressure (mmHg)	35.0 (28.0;49.0)	30.0 (24.0;39.0)	0.015 §
**Cardiac Magnetic Resonance**			
LV end-diastolic volume (mL)	126.5 (93.0;141.5)	137.0 (108.0;169.0)	0.008 §
**Holter**			
Rhythm : atrial fibrillation throughout + paroxysmal atrial fibrillation in sinus rhythm	25/123 (20.33%)	103/1039 (9.91%)	<0.001∼
**Exercise test**			
Absolute workload achieved (METS)	6.2 (5.0;7.0)	7.7 (5.7;10.7)	0.004 §
Max VO2 achieved (ml/min/Kg)	16.6 (13.8;21.0)	20.2 (16.9;25.9)	0.014 §
% of maximum estimated VO2 achieved Treadmill	30.4 (24.0;36.1)	37.8 (31.5;43.1)	0.002 §
**Device**			
Cardioverter defibrillator implanted	22/176 (12.50%)	324/1563 (20.73%)	0.010∼

**Key:** §: Kruskal-Wallis test; †: Exact-Fisher test; ∼: Chi-square test; NC: Not calculable. All continuous variables are presented as Median (Q1; Q3) and categorical variables as N and percentage. HCM, hypertrophic cardiomyopathy; SCD, sudden cardiac death; NYHA, New York Heart Association; BBB, bundle branch block; LBBB, left bundle branch block; LVEDD, left ventricular end-diastolic dimension; LVESD, left ventricular end-systolic dimension; LV, left ventricle; RV, right ventricle; VO2, oxygen consumption.

**Table 3:** Comparison between obese (body mass index≥30Kg/m2) and non-obese patients (variables with significant associations; please see supplemental table 3 for all comparisons and stratification according to genotype)

	Obese (*n* = 360)	Not obese (*n* = 1245)	*P*-value
**Demographics and co-morbidities**			
Age at first evaluation in the centre (years)	53.0 (44.0;62.0)	49.0 (35.0;61.0)	<0.001 §
Age at enrolment	58.0 (48.5;66.0)	54.0 (40.0;65.0)	<0.001 §
Body Mass Index (kg/m²)	32.7 (31.0;35.2)	25.4 (23.1;27.3)	<0.001 §
Hypertension	194/360 (53.89%)	393/1245 (31.57%)	<0.001∼
Diabetes mellitus II	57/360 (15.83%)	92/1245 (7.39%)	<0.001∼
Hyperlipidaemia/dyslipidaemia	191/360 (53.06%)	384/1245 (30.84%)	<0.001∼
Physical activity - no	111/266 (41.73%)	536/1007 (53.23%)	<0.001∼
Smoking	118/329 (35.87%)	343/1142 (30.04%)	0.045∼
**Symptoms**			
Age at first symptom (years)	49.0 (38.0;57.5)	43.0 (29.0;57.0)	0.015 §
Anginal chest pain	128/318 (40.25%)	351/1054 (33.30%)	0.023∼
NYHA class-I	71/309 (22.98%)	367/1010 (36.34%)	<0.001∼
II	167/309 (54.05%)	487/1010 (48.22%)	
III	68/309 (22.01%)	140/1010 (13.86%)	
IV	3/309 (0.97%)	16/1010 (1.58%)	
History of Atrial Fibrillation	111/360 (30.83%)	313/1245 (25.14%)	0.031∼
**ECG**			
Rhythm : Atrial fibrillation and Atrial flutter	40/355 (11.27%)	93/1226 (7.59%)	0.028∼
QT interval (ms)	436.0 (401.0;462.0)	426.0 (400.0;456.0)	0.003 §
PR interval (ms)	173.0 (156.0;197.0)	162.0 (145.0;190.0)	0.001 §
QRS duration (ms)	100.0 (90.0;120.0)	98.0 (88.0;112.0)	0.002 §
QRS axis (degrees)	30.0 (13.0;48.0)	39.0 (20.0;60.0)	<0.001 §
Maximum R in praecordial (mm)	14.0 (9.0;20.0)	16.0 (10.0;23.0)	0.002 §
Abn Q-waves	63/324 (19.44%)	286/1150 (24.87%)	0.042∼
**Echocardiogram**			
LVEDD (mm)	47.0 (43.0;51.0)	45.0 (40.2;49.0)	<0.001 §
LVESD (mm)	29.0 (25.0;33.0)	27.0 (23.0;32.0)	<0.001 §
Fractional shortening (%)	38.0 (32.0;44.0)	39.0 (33.0;44.0)	0.387 §
LVEDV (LV End Diastolic Volume) (mL)	97.0 (77.6;123.9)	90.0 (72.0;109.0)	0.004 §
LV posterior wall thickness diastole (mm)	12.0 (10.0;14.0)	11.0 (9.9;13.0)	0.005 §
Left atrium diameter (mm)	48.0 (42.9;52.0)	43.0 (38.0;49.0)	<0.001 §
Left atrial area (cm²)	28.0 (24.8;33.5)	24.7 (20.0;31.0)	<0.001 §
Maximum RV wall thickness (mm)	6.0 (5.0;8.0)	5.0 (4.0;7.0)	0.002 §
E-wave deceleration time (m/s)	217.5 (183.0;270.0)	200.0 (160.0;238.0)	<0.001 §
Mitral A-wave (m/s)	0.7 (0.5;0.9)	0.6 (0.5;0.8)	<0.001 §
Diastolic dysfunction - normal	36/266 (13.53%)	253/941 (26.89%)	<0.001∼
Maximum provoked (by any technique) peak left ventricular outflow tract gradient (mmHg)	23.0 (6.0;64.0)	12.0 (6.0;45.0)	0.036 §
LV ejection fraction (%)	70.5 (62.3;77.1)	67.1 (60.0;73.1)	0.021 §
**Cardiac Magnetic Resonance**			
Maximum LV thickness (mm)	20.0 (17.0;25.0)	19.0 (16.0;23.0)	0.018 §
**Exercise Test**			
Absolute workload achieved (METS)	6.0 (5.0;7.5)	7.8 (6.0;11.0)	<0.001 §
Max VO2 achieved (ml/min/Kg)	17.2 (14.0;20.2)	21.0 (17.5;27.2)	<0.001 §
% of maximum estimated VO2 achieved Treadmill	29.0 (23.6;32.2)	39.0 (34.0;44.0)	<0.001 §

**Key:** §: Kruskal-Wallis test; †: Exact-Fisher test; ∼: Chi-square test; NC: Not calculable. All continuous variables are presented as Median (Q1; Q3) and categorical variables as N and percentage. HCM, hypertrophic cardiomyopathy; SCD, sudden cardiac death; NYHA, New York Heart Association; BBB, bundle branch block; LBBB, left bundle branch block; LVEDD, left ventricular end-diastolic dimension; LVESD, left ventricular end-systolic dimension; LV, left ventricle; RV, right ventricle; VO2, oxygen consumption.

Supplementary *Figure 1* shows the prevalence and overlap of these risk factors in the study population. The majority of patients were male. For the overall population, there was no difference in sex distribution between hypertensives and non-hypertensives or between diabetics and non-diabetics. Females were more prevalent than males amongst the genotype-positive individuals with HT. In normal weight patients, women were more prevalent for the overall population and in genotype-positives.

Hypertension, diabetes, and overweight/obesity were associated with older age at symptom onset, first clinical evaluation and enrolment into the registry. The prevalence of a family history of HCM was greater in non-hypertensive, non-diabetic, and non-overweight/non-obese patients. Family history of SCD was also more prevalent in non-hypertensive and non-diabetic individuals.

Higher BMI and higher prevalence of smoking, dyslipidaemia, and sedentary lifestyle were noted in hypertensive and diabetic patients. Renal impairment, anaemia, and chronic obstructive pulmonary disease were more prevalent in hypertensive and diabetic patients.

### Associations between hypertension and clinical phenotype (Table 1, Figure 1, Supplemental material)

Hypertension was associated with higher NYHA functional class, more paroxysmal nocturnal dyspnoea and ankle oedema, and more frequent chest pain. Unexplained syncope was more common in non-hypertensive patients. Absolute workload achieved in metabolic equivalents (METS) and peak oxygen consumption (peak VO2 in mL/min/Kg) were lower in hypertensives. A past history of AF and stroke were more prevalent in hypertensives. AF was more prevalent on the baseline ECG and Holter for the overall population and in the genotype-positive HT patient subgroup only. A previous resuscitated ventricular fibrillation/arrest episode was more frequent in non-hypertensives, who had a higher prevalence of ICDs (implantable cardioverter-defibrillators) at baseline.

**Figure 1:**
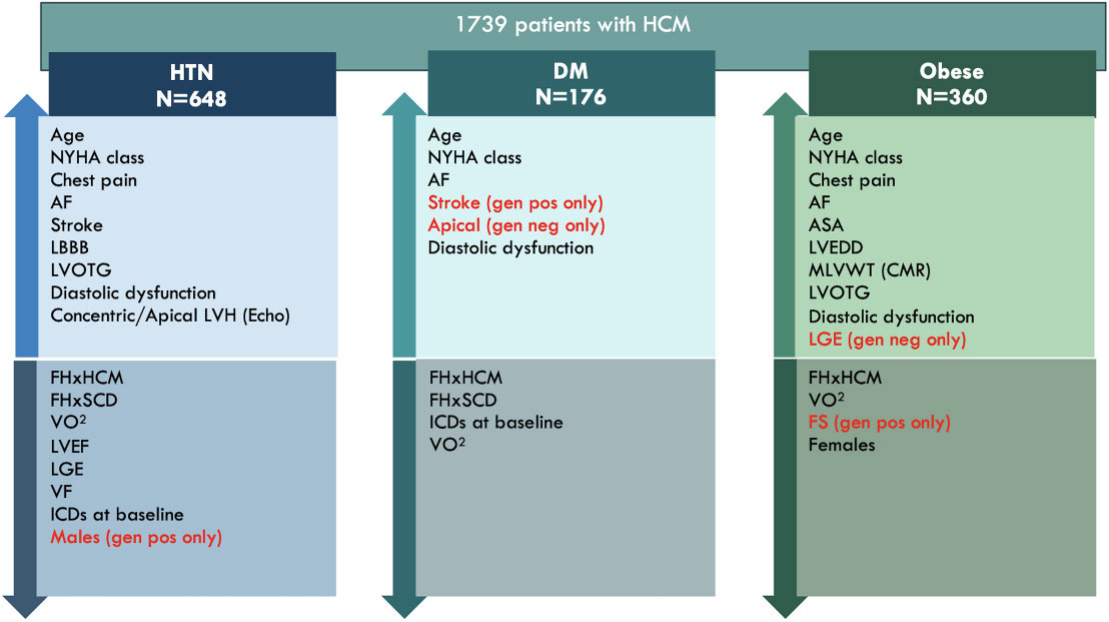
Associations of common cardiovascular risk factors with HCM phenotype. Up-arrows represent positive associations and down-arrows represent negative associations. HTN, hypertension; DM, diabetes mellitus; NYHA, New York Heart Association; AF, atrial fibrillation; LBBB, left bundle branch block; LVTOG, left ventricular outflow tract gradient; LVH, left ventricular hypertrophy; FHxSCD, family history of sudden cardiac death; VO2, maximal oxygen consumption on the CPET; LVEF, left ventricular ejection fraction; LGE, late gadolinium enhancement; VF, ventricular fibrillation; ICD, implantable cardioverter-defibrillator; ASA, alcohol septal ablation; LVEDD, left ventricular end-diastolic dimension; MLVWT, maximal left ventricular wall thickness; CMR, cardiac magnetic resonance; FS, fractional shortening.

Left bundle branch block (LBBB) was more frequent in hypertensives. Maximum R wave amplitude in the precordial leads was higher in hypertensive individuals for the global cohort, but lower for the genotype-positive individuals with HT compared to the non-hypertensives. Hypertensive individuals had lower ejection fraction and hypertensive genotype-positive patients had lower fractional shortening (FS). Left ventricular (LV) posterior wall thickness was greater in hypertensives and concentric and apical patterns of hypertrophy were more frequent on echocardiography. Diastolic dysfunction was more prevalent in the presence of HT and hypertensive patients had larger left atria and higher estimated pulmonary artery systolic pressures (PASP). Left ventricular outflow tract gradients were higher in hypertensive individuals, both at rest and upon provocation. Late gadolinium enhancement (LGE) on cardiac MRI was more prevalent in non-hypertensives.

### Associations between diabetes and clinical phenotype (Table 2, Figure 1, Supplemental material)

Diabetes was associated with higher NYHA functional class. Absolute workload achieved during exercise testing in METS and peak VO2 were lower in the presence of diabetes.

A history of AF and stroke were more prevalent amongst genotype-positive diabetic patients and AF was more prevalent in the baseline ECG and Holter, both for the overall diabetic population and when analysing the genotype-positive patient subgroup. Diabetic patients less frequently had ICDs implanted at baseline.

Apical HCM was more prevalent in diabetic compared to non-diabetic patients, when analysing the genotype-negative subcohort. Diastolic dysfunction was more frequent in the presence of DM and diabetics had higher pulmonary artery systolic pressures.

### Associations between obesity and clinical phenotype (Table 3, Figure 1 and Supplemental material)

Obesity was associated with higher NYHA functional class and chest pain. A previous history of alcohol septal ablation was more frequent in overweight/obese patients. Absolute workload achieved in METS and peak VO2 were lower in the presence of obesity.

A history of AF and AF on the ECG was more prevalent in the presence of obesity.

Left bundle branch block (LBBB) was more frequent in overweight/obese patients.

Obese-overweight patients had larger LV ventricular dimension and the genotype-positive individuals had lower FS. LV posterior wall thickness was greater. Maximum LV wall thickness on CMR but not echocardiography was higher in obese patients. RV wall thickness was greater in obese patients. Diastolic dysfunction was more frequent in the presence of Ob and left atria larger. Left ventricular outflow tract gradients were higher at provocation in obese. Late gadolinium enhancement (LGE) on CMR was more prevalent in genotype-negative obese patients.

### Multivariable analysis (Table 4 and Supplemental material)

Hypertension and obesity were independently associated with ≥2 NYHA functional class at presentation, when controlling for age, sex, maximal wall thickness, AF, LA diameter, proband vs. relative status and genotype. The analysed risk factors were not independently associated with maximal wall thickness, AF, or LVEF (supplemental material).

**Table 4:** Univariate and multivariate logistic regression analysis for NYHA superior or equal to 2

				Univariate		Multivariate	
Variable	Modality	Number of patients with NYHA > = 2 (*n* = 954)	Number of patients with NYHA = 1 (*n* = 463)	OR [95% CI]	OR *P*-value	OR [95% CI]	OR *P*-value
**Hypertension**	No	538/954 (56.39%)	344/463 (74.30%)	/	/		
	Yes	416/954 (43.61%)	119/463 (25.70%)	2.235 [1.750-2.855]	<0.001	1.411 [1.029-1.935]	0.032
**Diabetes mellitus**	No diabetes	829/954 (86.90%)	433/463 (93.52%)	/	/		
	Diabetes	125/954 (13.10%)	30/463 (6.48%)	2.176 [1.437-3.296]	<0.001		
**Obesity**	Not obese	646/954 (67.71%)	367/463 (79.27%)	/	/		
	Obese	239/954 (25.05%)	71/463 (15.33%)	1.912 [1.425-2.567]	<0.001	1.577 [1.107-2.245]	0.012
**Age at enrolment**		954/954 (mean = 56.9)	463/463 (mean = 48.7)	1.034 [1.027-1.042]	<0.001	1.020 [1.011-1.030]	<0.001
**Males**	Male	492/954 (51.57%)	316/463 (68.25%)	/	/		
	Female	462/954 (48.43%)	147/463 (31.75%)	2.019 [1.599-2.549]	<0.001	2.073 [1.567-2.742]	<0.001
**Genotype**	Genotype-negative	68/954 (7.13%)	28/463 (6.05%)	/	/		
	Genotype-positive	163/954 (17.09%)	100/463 (21.60%)	0.671 [0.405-1.113]	0.122		
**Maximum LV thickness (mm)**		880/954 (mean = 19.8)	436/463 (mean = 19.5)	1.011 [0.988-1.035]	0.343		
**Maximum provoked (by any technique) peak left ventricular outflow tract gradient (mmHg)**		283/954 (mean = 39.5)	108/463 (mean = 29.8)	1.007 [1.001-1.013]	0.032		
**Left atrium diameter (mm)**		816/954 (mean = 45.8)	399/463 (mean = 42.6)	1.047 [1.031-1.063]	<0.001	1.031 [1.013-1.049]	<0.001
**Atrial Fibrillation**	No	582/954 (61.01%)	379/463 (81.86%)	/	/		
	Yes	346/954 (36.27%)	79/463 (17.06%)	2.852 [2.163-3.761]	<0.001	2.092 [1.497-2.925]	<0.001
**Proband or relative**	Relative	109/954 (11.43%)	107/463 (23.11%)	/	/		
	Proband	612/954 (64.15%)	209/463 (45.14%)	2.874 [2.109-3.917]	<0.001		
**Age at diagnosis (years)**		940/954 (mean = 48.6)	459/463 (mean = 40.5)	1.025 [1.019-1.032]	<0.001		

**Key:** NC, Not calculable due to few or no events; #, Reference; LV, left ventricle Covariates significant at 20% and at least 80% of available data are selected from univariate analysis. After that a stepwise logistic regression with slentry = 0.05 and slstay = 0.05 is performed. Finally a final model will be rerun with selected covariates.

## Discussion

This study shows that patients with HCM enrolled into the EORP registry have a high prevalence of cardiovascular risk factors, comparable with data in the general European population.^11^ Hypertension, diabetes, and obesity were associated with older age at presentation, a lower prevalence of family history of HCM and SCD, more symptoms, frequent AF and worse LV diastolic function. Hypertension and obesity were associated with higher provocable LV outflow tract gradients and LV hypertrophy was more severe in obese patients. Hypertension and obesity were independently associated with more advanced NYHA class.

### Relation between risk factors and clinical phenotype

The association between the severity of cardiac disease and CVRF that cause left ventricular hypertrophy could suggest that the diagnosis of HCM was incorrect in some individuals. For example, the differential diagnosis between HCM and LVH in the context of hypertensive heart disease is often challenging in the presence of less severe hypertrophy, even when using guideline suggested thresholds of wall thickness to aid in the differential.^1^ While this is potentially supported by the lower prevalence of a family history of HCM and an older age in patients with risk factors, the presence of similar phenotype and family history associations in patients with and without a positive genotype suggests a more complex explanation. For example, it is possible that the diagnosis of HCM was in fact delayed in some individuals due to misattribution of their phenotype to HT or obesity at first presentation.^12,13^ Another possibility is that HT triggers phenotype development in families where the mutation is less penetrant, as suggested previously in one study of a founder mutation in *MYL2*.^3^

The presence of more severe disease in patients with risk factors is likely to be multifactorial. For example, a higher prevalence of LVOTO in obese patients may be related to the more severe hypertrophy. Other studies^2,14^ also reported more prevalent LVOTO in obese patients and hypothesized that higher stroke volumes were responsible.

A recent study has described an association between diabetes with symptoms, diastolic dysfunction, and higher mortality in HCM.^4^ In the registry data hereby analysed, we found similar findings including worse diastolic dysfunction, larger left atria, and higher PASP. This could suggest a common pathophysiology similar to that proposed for diabetic cardiomyopathy.^15^ In contrast to the previously mentioned publication,^4^ we did not observe an association with mitral regurgitation, conduction disease, or pacemaker implantation in diabetics.

The proportion of individuals with fibrosis detected by CMR was higher in non-hypertensives and in genotype-negative obese patients, but data regarding LGE percentage of total LV mass, LGE location, or other MRI techniques such as T1 mapping for quantification of diffuse fibrosis were not available to characterize this finding in more detail. However, a higher prevalence of LGE in obese patients has been previously observed.^2^

### Risk factors and prognosis

As this was a cross-sectional analysis of the EORP registry, it is not possible to determine the influence of common risk factors on outcomes in HCM. Nevertheless, as in the general population,^16^ a history of stroke was more frequent in patients with HT and diabetes and AF, particularly in older patients with diabetes.^17^ In addition, LV systolic function was more severely impaired in genotype-positive hypertensive patients and genotype-positive obese patients, a relevant finding as evolution to systolic dysfunction is an ominous prognostic feature in HCM that occurs in 5–10% of patients.^18^

These observations highlight the need for stricter control of these risk factors in HCM patients.

Non-diabetic patients had more ICDs implanted at baseline; recent data reporting higher 15-year mortality,^4^ has shown no differences in SCD rate or appropriate ICD therapy in diabetic patients with HCM. The increased frequency of syncope in non-hypertensives, who also had more family history of SCD and more episodes of resuscitated cardiac arrest and more ICDs at baseline, seems to point towards a more severe arrhythmogenic phenotype in this group. One possible explanation is that the genetic mutation is more penetrant in these patients and thus correlates with greater arrhythmic risk.

### Interaction between genotype and common cardiovascular risk factors

Many of the associations between risk factors and phenotype were significant in patients with and without a positive genotype (age, family history of HCM, LA dimension, peak VO2, and AF) and others were specific to genotype-positive patients. For example, there were more female than male hypertensive mutation carriers. This is contrary to the usual sex distribution in HCM and suggests a putative modifier effect of HT that might be more marked in women. In contrast, there were more obese men than women, suggesting a stronger environment-gene interaction for obesity in males with HCM.

LV systolic function was impaired only in genotype-positive hypertensive or obese patients. This suggests a synergistic effect of risk factor and genotype in systolic function. Similarly, stroke was more prevalent in genotype-positive diabetic patients, but not in the overall population or genotype-negative diabetics. A recent association was described for an increased incidence of AF in *MYH7* HCM patients of a large US based multicentre study, which may be in keeping with this finding.^19^

### Clinical implications

The prevalence of risk factors in this European HCM cohort is in accord with data in the general European population.^11^ The detrimental effects of these factors in cardiovascular and non-cardiovascular mortality are well known,^16^ but this study suggests that cardiovascular risk factors have an additional impact on the clinical expression of HCM. Cardiovascular risk factor assessment is therefore essential as stricter control of these risk factors in patients with HCM might contribute to a lower symptom burden and improved outcomes.

### Future research

Future studies should focus on analysing the longitudinal impact of CVRF in HCM prognosis, including cardiovascular mortality and sudden death outcomes. Such studies should also analyse gene-environment-phenotype interactions at both a rare variant and common variant/polygenic level.

### Limitations

In common with other registry-based studies with voluntary participation, selection bias can be present due to inclusion of more severe/symptomatic patients from referral centres and the cohort might not be fully representative of the overall European HCM patient population. Also in common with other registry-based studies, each centre was expected to follow contemporary guidelines for the correct definition of HT and DM, which always has the potential to introduce a level of heterogeneity in data collection.

The cross-sectional nature of the data prevents further assessment of the prognostic impact of the analysed risk factors.

Ethnicity or coronary artery disease data were not available, as these parameters were not included as part of the study design and as such not systematically collected on the registry's CRF.

Gene-risk factor interactions could only be analysed in the genotyped patients. Hence, sub-analyses focused on genetic results are small and challenging to interpret.

### Conclusions

In this large multicentre European cohort, we have observed that CVRF are associated with clinical and imaging phenotype traits in HCM. These findings highlight the importance of assessing and treating comorbid risk factors in this population.

## Supplementary Material

qcac006_Supplemental_Files

## Notes

**Conflicts of interest:** Dr. Lopes reports consulting fees from Bristol-Meyer-Squibb. Dr. Charron reports personal fees from Amicus, personal fees from Pfizer, grants from Sanofi, grants from Shire, outside the submitted work. Dr. Kaski reports grants from British Heart Foundation, grants from Medical Research Council, outside the submitted work; Dr. Maggioni reports personal fees from Bayer, personal fees from Novartis, personal fees from Fresenius, outside the submitted work; Dr. Tavazzi reports personal fees from Servier, personal fees from CVIE Therapeutics, outside the submitted work; Dr. Čelutkienė reports personal fees from Novartis, personal fees from AstraZeneca, personal fees from Boehringer Ingelheim, personal fees from Servier, personal fees from Amgen, outside the submitted work. Dr Arbustini, Cécile Laroche, Dr. Brito, Dr Caforio, Dr Elliott, Dr Gimeno, Dr Hagege, Dr Linhart, Dr Mogensen, Dr Ripoll-Vera, Dr Seggewiss, Dr Sheikh, Dr Losi, Dr Manuel Garcia-Pinilla, Dr Villacorta have nothing to disclose.

## Data availability

The majority of data underlying this article are available in the article and in its online supplementary material. Any additional data will be shared on reasonable request to the corresponding author

## Appendix 1.

**EORP Oversight Committee**

Christopher Peter Gale, Chair, GB, Branko Beleslin, RS, Andrzej Budaj, PL, Ovidiu Chioncel, RO, Nikolaos Dagres, DE, Nicolas Danchin, FR, David Erlinge, SE, Jonathan Emberson, GB, Michael Glikson, IL, Alastair Gray, GB, Meral Kayikcioglu, TR, Aldo Maggioni, IT, Klaudia Vivien Nagy, HU, Aleksandr Nedoshivin, RU, Anna-Sonia Petronio, IT, Jolien Roo Hesselink, NL, Lars Wallentin, SE, Uwe Zeymer, DE.

**Executive Committee**

Alida Caforio (Chair), IT, Juan Ramon Gimeno Blanes, ES, Philippe Charron, FR, Perry Elliott, GB, Juan Pablo Kaski, GB, Aldo P. Maggioni, IT, Luigi Tavazzi, IT, Michal Tendera, PL

**Investigators**

Belarus: *Minsk:* S. Komissarova, N. Chakova, S. Niyazova, Czech Republic: *Prague:*A. Linhart, P. Kuchynka, T. Palecek, J. Podzimkova, M. Fikrle, E. Nemecek, Denmark: *Copenhagen:* H. Bundgaard, J. Tfelt-Hansen, J. Theilade, J.J. Thune, A. Axelsson, *Odense:* J. Mogensen, F. Henriksen, T. Hey, S.K. Nielsen, L. Videbaek, S. Andreasen, H. Arnsted, Egypt: *Zagazig:* A. Saad, M. Ali, Finland: *Helsinki:* J. Lommi, T. Helio, M.S. Nieminen, France: *Boulogne-Billancourt:* O. Dubourg, N. Mansencal, M. Arslan, V. Siam Tsieu, *Créteil:* T. Damy, A. Guellich, S. Guendouz, C.M. Tissot, A. Lamine, S. Rappeneau, *Paris:* A. Hagege, M. Desnos, A. Bachet, M. Hamzaoui, *Paris:* P. Charron, R. Isnard, L. Legrand, C. Maupain, E. Gandjbakhch, M. Kerneis, J-F. Pruny, Germany: *Crailsheim:* A. Bauer, B. Pfeiffer, *Greifswald:* S.B. Felix, M. Dorr, S. Kaczmarek, K. Lehnert, A-L. Pedersen, D. Beug, M. Bruder, *Homburg/Saar:* M. Böhm, I. Kindermann, Y. Linicus, C. Werner, B. Neurath, M. Schild-Ungerbuehler, *Schweinfurt:* H. Seggewiss, B. Pfeiffer, A. Neugebauer, Great Britain: *Belfast:* P. McKeown, A. Muir, J. McOsker, T. Jardine, G. Divine, *London:* P. Elliott, M. Lorenzini, O. Watkinson, E. Wicks, *London:* H. Iqbal, S. Mohiddin, C. O'Mahony, N. Sekri, *London:* G. Carr-White, T. Bueser, R. Rajani, L. Clack, J. Damm, S. Jones, R. Sanchez-Vidal, M. Smith, T. Walters, K. Wilson, *London:* S. Rosmini, Greece: *Athens:* A. Anastasakis, K. Ritsatos, V. Vlagkouli, Hungary: *Szeged:* T. Forster, R. Sepp, J. Borbas, V. Nagy, A. Tringer, K. Kakonyi, L.A. Szabo, Iran: *Tehran:* M. Maleki, F. Noohi Bezanjani, A. Amin, N. Naderi, M. Parsaee, S. Taghavi, B. Ghadrdoost, S. Jafari, M. Khoshavi, Italy: *Bologna:* C. Rapezzi, E. Biagini, A. Corsini, C. Gagliardi, M. Graziosi, S. Longhi, A. Milandri, L. Ragni, S. Palmieri, *Florence:* I. Olivotto, A. Arretini, G. Castelli, F. Cecchi, A. Fornaro, B. Tomberli, *Genoa:* P. Spirito, E. Devoto, *Milan:* P. Della Bella, G. Maccabelli, S. Sala, F. Guarracini, G. Peretto, *Naples:* M.G. Russo, R. Calabro, G. Pacileo, G. Limongelli, D. Masarone, V. Pazzanese, A. Rea, M. Rubino, S. Tramonte, F. Valente, M. Caiazza, A. Cirillo, G. Del Giorno, A. Esposito, R. Gravino, T. Marrazzo, *Naples:* B. Trimarco, M-A. Losi, C. Di Nardo, A. Giamundo, F. Musella, F. Pacelli, A. Scatteia, G. Canciello, *Padua:* A. Caforio, S. Iliceto, C. Calore, L. Leoni, M. Perazzolo Marra, I. Rigato, G. Tarantini, A. Schiavo, M. Testolina, *Pavia:* E. Arbustini, A. Di Toro, L.P. Giuliani, A. Serio. *Rome:* F. Fedele, A. Frustaci, M. Alfarano, C. Chimenti, *Rome:* F. Drago, A. Baban, *Rome:* L. Calò, C. Lanzillo, A. Martino, *Rome:* M. Uguccioni, E. Zachara, G. Halasz, F. Re, *Trieste:* G. Sinagra, C. Carriere, M. Merlo, F. Ramani, Lithuania: *Kaunas:* A. Kavoliuniene, A. Krivickiene, E. Tamuleviciute-Prasciene, M. Viezelis, *Vilnius:* J. Celutkiene, L. Balkeviciene, M. Laukyte, E. Paleviciute, Netherlands: *Amsterdam:* Y. Pinto, A. Wilde, *Utrecht:* F.W. Asselbergs, A. Sammani, J. Van Der Heijden, L. Van Laake, N. De Jonge, R. Hassink, J.H. Kirkels, Nigeria: *Lagos:* J. Ajuluchukwu, A. Olusegun-Joseph, E. Ekure, Poland: *Katowice:* K. Mizia-Stec, M. Tendera, A. Czekaj, A. Sikora-Puz, A. Skoczynska, M. Wybraniec, *Krakow:* P. Rubis, E. Dziewiecka, S. Wisniowska-Smialek, *Warsaw:* Z. Bilinska, P. Chmielewski, B. Foss- Nieradko, E. Michalak, M. Stepien-Wojno, B. Mazek, Portugal: *Almada:* L. Rocha Lopes, A.R. Almeida, I. Cruz, A.C. Gomes, A.R. Pereira, *Lisbon:* D. Brito, H. Madeira, A.R. Francisco, M. Menezes, O. Moldovan, T. Oliveira Guimaraes, D. Silva, Romania: *Bucharest:* C. Ginghina, R. Jurcut, A. Mursa, B.A. Popescu, E. Apetrei, S. Militaru, I. Mircea Coman, *Targu-Mures:* A. Frigy, Z. Fogarasi, I. Kocsis, I.A. Szabo, L. Fehervari, Russian Federation: *Moscow:* I. Nikitin, E. Resnik, M. Komissarova, V. Lazarev, M. Shebzukhova, D. Ustyuzhanin, *Moscow:* O. Blagova, I. Alieva, V. Kulikova, Y. Lutokhina, E. Pavlenko, N. Varionchik, Serbia: *Belgrade:* A.D. Ristic, P.M. Seferovic, I. Veljic, I. Zivkovic, I. Milinkovic, A. Pavlovic, G. Radovanovic, D. Simeunovic, *Belgrade:* M. Zdravkovic, M. Aleksic, J. Djokic, S. Hinic, S. Klasnja, K. Mircetic, Spain: *A Coruna:* L. Monserrat, X. Fernandez, D. Garcia-Giustiniani, J.M. Larrañaga, M. Ortiz-Genga, R. Barriales-Villa, C. Martinez-Veira, E. Veira, *Barcelona:* A. Cequier, J. Salazar-Mendiguchia, N. Manito, J. Gonzalez, *Madrid:* F. Fernández-Avilés, C. Medrano, R. Yotti, S. Cuenca, M.A. Espinosa, I. Mendez, E. Zatarain, R. Alvarez, *Madrid:* P. Garcia Pavia, A. Briceno, M. Cobo-Marcos, F. Dominguez, *Malaga:* E. De Teresa Galvan, J.M. García Pinilla, N. Abdeselam-Mohamed, M.A. Lopez-Garrido, L. Morcillo Hidalgo, M.V. Ortega-Jimenez, A. Robles Mezcua, A. Guijarro-Contreras, D. Gomez-Garcia, M. Robles-Mezcua, *Murcia:* J.R. Gimeno Blanes, F.J. Castro, C. Munoz Esparza, M. Sabater Molina, M. Sorli García, D. Lopez Cuenca, *Palma de Mallorca:* T. Ripoll-Vera, J. Alvarez, J. Nunez, Y. Gomez, *Salamanca:* P.L. Sanchez Fernandez, E. Villacorta, C. Avila, L. Bravo, E. Diaz-Pelaez, M. Gallego-Delgado, L. Garcia-Cuenllas, B. Plata, *Seville:* J.E. Lopez-Haldon, M.L. Pena Pena, E.M. Cantero Perez, *Valencia:* E. Zorio, M.A. Arnau, J. Sanz, E. Marques-Sule.

## Notes

Listed in the Appendix 1
