# Supplementary material for: Association between common cardiovascular risk factors and clinical phenotype in patients with hypertrophic cardiomyopathy from the European Society of Cardiology (ESC) EurObservational Research Programme (EORP) Cardiomyopathy/Myocarditis registry
Source: Eur Heart J Qual Care Clin Outcomes. 2022 Feb 9;9(1):42–53. doi: 10.1093/ehjqcco/qcac006 (PMC9745665; doi:10.1093/ehjqcco/qcac006)
Supplement: qcac006_Supplemental_Files [file qcac006_supplemental_files.zip › Supplemental tables.docx]

# **Supplemental table 1. Comparison between hypertensive and non-hypertensive patients.**

|  | | **All** | | | **Genotype-positive** | | | **Genotype-negative** | | |
| --- | --- | --- | --- | --- | --- | --- | --- | --- | --- | --- |
|  |  | **No HTN (N=1091)** | **HTN (N=648)** | **P-value** | **No HTN (N=257)** | **HTN (N=62)** | **P-value** | **No HTN (N=87)** | **HTN (N=44)** | **P-value** |
| **Demographic variables and co-mobidities** | | | | | | | | | | |
| Age at first evaluation (years) | Median (Q1 ; Q3) | 44.0 (32.0;55.0) | 60.0 (51.0;67.0) | <0.001 **(S)** § | 40.0 (27.0;50.0) | 58.0 (50.0;65.5) | <0.001 **(S)** § | 46.0 (31.5;60.0) | 57.5 (50.5;68.0) | <0.001 **(S)** § |
| Age at enrolment (years) | Median (Q1 ; Q3) | 48.0 (37.0;60.0) | 63.0 (55.0;71.0) | <0.001 **(S)** § | 46.0 (36.0;57.0) | 63.0 (56.0;71.0) | <0.001 **(S)** § | 53.0 (42.0;66.0) | 64.0 (57.0;70.0) | <0.001 **(S)** § |
| Gender | Female | 434/1091 (39.78%) | 277/648 (42.75%) | 0.224(NS)~ | 108/257 (42.02%) | 39/62 (62.90%) | 0.003**(S)**~ | 26/87 (29.89%) | 16/44 (36.36%) | 0.453(NS)~ |
|  | Male | 657/1091 (60.22%) | 371/648 (57.25%) |  | 149/257 (57.98%) | 23/62 (37.10%) |  | 61/87 (70.11%) | 28/44 (63.64%) |  |
| Body Mass Index (kg/m²) | Median (Q1 ; Q3) | 25.7 (23.2;28.4) | 27.8 (25.1;31.1) | <0.001 **(S)** § | 25.6 (23.1;28.4) | 27.6 (25.0;31.4) | <0.001 **(S)** § | 25.9 (23.7;29.7) | 29.1 (25.0;31.2) | 0.046 **(S)** § |
| Family history of HCM |  | 505/940 (53.72%) | 156/524 (29.77%) | <0.001**(S)**~ | 191/241 (79.25%) | 39/59 (66.10%) | 0.042**(S)**~ | 33/77 (42.86%) | 11/41 (26.83%) | 0.230(NS)~ |
| Family history of SCD |  | 241/1041 (23.15%) | 109/621 (17.55%) | 0.007**(S)**~ | 83/254 (32.68%) | 21/62 (33.87%) | 0.858(NS)~ | 20/83 (24.10%) | 7/42 (16.67%) | 0.340(NS)~ |
| Diabetes mellitus II |  | 47/1091 (4.31%) | 116/648 (17.90%) | <0.001**(S)**~ | 9/257 (3.50%) | 8/62 (12.90%) | 0.007**(S)**† | 7/87 (8.05%) | 7/44 (15.91%) | 0.231(NS)† |
| Hyperlipidaemia/dyslipidaemia |  | 266/1091 (24.38%) | 369/648 (56.94%) | <0.001**(S)**~ | 52/257 (20.23%) | 35/62 (56.45%) | <0.001**(S)**~ | 27/87 (31.03%) | 23/44 (52.27%) | 0.018**(S)**~ |
| Physical activity |  | 455/867 (52.48%) | 217/499 (43.49%) | 0.001**(S)**~ | 108/226 (47.79%) | 8/43 (18.60%) | <0.001**(S)**~ | 33/61 (54.10%) | 13/25 (52.00%) | 0.859(NS)~ |
| Smoking (Y/N) |  | 295/995 (29.65%) | 206/591 (34.86%) | 0.031**(S)**~ | 70/228 (30.70%) | 19/51 (37.25%) | 0.364(NS)~ | 14/67 (20.90%) | 11/35 (31.43%) | 0.240(NS)~ |
| Renal impairment |  | 55/1091 (5.04%) | 103/648 (15.90%) | <0.001**(S)**~ | 6/257 (2.33%) | 8/62 (12.90%) | 0.002**(S)**† | 8/87 (9.20%) | 9/44 (20.45%) | 0.070(NS)~ |
| Anaemia |  | 36/1076 (3.35%) | 44/641 (6.86%) | <0.001**(S)**~ | 4/254 (1.57%) | 2/59 (3.39%) | 0.316(NS)† | 3/87 (3.45%) | 3/44 (6.82%) | 0.403(NS)† |
| Chronic obstructive pulmonary disease |  | 25/1091 (2.29%) | 42/648 (6.48%) | <0.001**(S)**~ | 4/257 (1.56%) | 4/62 (6.45%) | 0.049**(S)**† | 3/87 (3.45%) | 4/44 (9.09%) | 0.224(NS)† |
| **Symptoms** | | | | | | | | | | |
| Age at first symptom (years) | Median (Q1 ; Q3) | 39.0 (25.0;51.5) | 55.0 (45.0;63.0) | <0.001 (S) § | 34.0 (22.0;47.0) | 50.5 (41.0;59.0) | <0.001 (S) § | 44.0 (22.0;58.0) | 56.0 (47.5;66.5) | 0.012 (S) § |
| Unexplained syncope (suspected arrhythmic cardiogenic+mechanism uncertain) |  | 178/900 (19.78%) | 85/553 (15.37%) | 0.034(S)~ | 44/212 (20.75%) | 8/55 (14.55%) | 0.300(NS)~ | 10/62 (16.13%) | 5/35 (14.29%) | 0.809(NS)~ |
| Anginal chest pain |  | 268/911 (29.42%) | 245/559 (43.83%) | <0.001(S)~ | 50/213 (23.47%) | 17/55 (30.91%) | 0.256(NS)~ | 20/63 (31.75%) | 11/35 (31.43%) | 0.974(NS)~ |
| NYHA class | NYHA I | 344/881 (39.05%) | 119/532 (22.37%) | <0.001(S)~ | 85/210 (40.48%) | 15/52 (28.85%) | 0.288(NS)~ | 18/61 (29.51%) | 10/35 (28.57%) | 0.739(NS)† |
|  | NYHA II | 399/881 (45.29%) | 306/532 (57.52%) |  | 95/210 (45.24%) | 29/52 (55.77%) |  | 32/61 (52.46%) | 16/35 (45.71%) |  |
|  | NYHA III | 127/881 (14.42%) | 99/532 (18.61%) |  | 30/210 (14.29%) | 8/52 (15.38%) |  | 10/61 (16.39%) | 9/35 (25.71%) |  |
|  | NYHA IV | 11/881 (1.25%) | 8/532 (1.50%) |  | 0/210 (0.00%) | 0/52 (0.00%) |  | 1/61 (1.64%) | 0/35 (0.00%) |  |
| NYHA functional class > II |  | 138/881 (15.66%) | 107/532 (20.11%) | 0.032(S)~ | 30/210 (14.29%) | 8/52 (15.38%) | 0.840(NS)~ | 11/61 (18.03%) | 9/35 (25.71%) | 0.372(NS)~ |
| Palpitations |  | 358/911 (39.30%) | 189/559 (33.81%) | 0.035(S)~ | 82/213 (38.50%) | 18/55 (32.73%) | 0.430(NS)~ | 30/63 (47.62%) | 13/35 (37.14%) | 0.317(NS)~ |
| Orthopnea |  | 60/911 (6.59%) | 50/559 (8.94%) | 0.095(NS)~ | 11/213 (5.16%) | 3/55 (5.45%) | 1.000(NS)† | 5/63 (7.94%) | 3/35 (8.57%) | 1.000(NS)† |
| Ankle oedema |  | 55/911 (6.04%) | 52/559 (9.30%) | 0.019(S)~ | 9/213 (4.23%) | 4/55 (7.27%) | 0.312(NS)† | 2/63 (3.17%) | 2/35 (5.71%) | 0.615(NS)† |
| Paroxysmal nocturnal dyspnea |  | 37/911 (4.06%) | 37/559 (6.62%) | 0.029(S)~ | 8/213 (3.76%) | 2/55 (3.64%) | 1.000(NS)† | 3/63 (4.76%) | 2/35 (5.71%) | 1.000(NS)† |
| **Arrhythmia history** | | | | | | | | | | |
| History of Atrial Fibrillation |  | 258/1091 (23.65%) | 205/648 (31.64%) | <0.001(S)~ | 64/257 (24.90%) | 25/62 (40.32%) | 0.015(S)~ | 22/87 (25.29%) | 13/44 (29.55%) | 0.603(NS)~ |
| History of stroke | No | 1037/1091 (95.05%) | 591/648 (91.20%) | <0.001(S)~ | 240/257 (93.39%) | 53/62 (85.48%) | 0.064(NS)† | 83/87 (95.40%) | 40/44 (90.91%) | 0.212(NS)† |
|  | TIA | 23/1091 (2.11%) | 18/648 (2.78%) |  | 8/257 (3.11%) | 2/62 (3.23%) |  | 0/87 (0.00%) | 2/44 (4.55%) |  |
|  | Stroke | 22/1091 (2.02%) | 37/648 (5.71%) |  | 6/257 (2.33%) | 5/62 (8.06%) |  | 3/87 (3.45%) | 2/44 (4.55%) |  |
| Sustained ventricular tachycardia |  | 92/1091 (8.43%) | 42/648 (6.48%) | 0.140(NS)~ | 24/257 (9.34%) | 8/62 (12.90%) | 0.402(NS)~ | 5/87 (5.75%) | 3/44 (6.82%) | 1.000(NS)† |
| Resuscitated ventricular fibrillation/cardiac arrest |  | 41/1091 (3.76%) | 8/648 (1.23%) | 0.002(S)~ | 14/257 (5.45%) | 2/62 (3.23%) | 0.746(NS)† | 2/87 (2.30%) | 1/44 (2.27%) | 1.000(NS)† |
| History of BBB |  | 70/607 (11.53%) | 82/451 (18.18%) | 0.002(S)~ | 15/141 (10.64%) | 7/33 (21.21%) | 0.141(NS)† | 16/71 (22.54%) | 6/37 (16.22%) | 0.439(NS)~ |
| **ECG** | | | | | | | | | | |
| Rhythm : Atrial fibrillation and Atrial flutter |  | 73/1056 (6.91%) | 74/628 (11.78%) | <0.001(S)~ | 14/252 (5.56%) | 12/60 (20.00%) | <0.001(S)~ | 6/87 (6.90%) | 4/44 (9.09%) | 0.732(NS)† |
| QT interval (ms) | Median (Q1 ; Q3) | 425.5 (400.0;454.0) | 434.0 (400.0;462.0) | 0.023 (S) § | 430.0 (400.0;452.0) | 435.0 (400.0;462.0) | 0.723 (NS) § | 436.0 (416.0;480.0) | 427.0 (400.0;456.0) | 0.080 (NS) § |
| PR interval (ms) | Median (Q1 ; Q3) | 160.0 (144.0;188.0) | 170.0 (154.0;200.0) | <0.001 (S) § | 162.0 (146.0;190.0) | 179.0 (160.0;198.0) | 0.030 (S) § | 166.0 (141.0;201.0) | 180.0 (160.0;194.0) | 0.297 (NS) § |
| QRS duration (ms | Median (Q1 ; Q3) | 98.0 (88.0;112.0) | 100.0 (90.0;120.0) | 0.004 (S) § | 98.0 (86.0;112.0) | 100.0 (90.0;108.0) | 0.507 (NS) § | 104.0 (93.0;130.0) | 98.0 (90.0;106.0) | 0.029 (S) § |
| QRS axis (degrees) | Median (Q1 ; Q3) | 40.0 (20.0;60.0) | 30.0 (15.0;50.0) | <0.001 (S) § | 38.5 (15.5;60.0) | 29.0 (12.5;46.0) | 0.026 (S) § | 39.0 (18.0;60.0) | 30.0 (10.0;45.0) | 0.138 (NS) § |
| Atrioventricular block : 1st degree |  | 97/1053 (9.21%) | 80/628 (12.74%) | 0.023(S)~ | 23/252 (9.13%) | 10/60 (16.67%) | 0.088(NS)~ | 14/87 (16.09%) | 4/44 (9.09%) | 0.272(NS)~ |
| Bundle branch block : Incomplete LBBB+LBBB |  | 92/983 (9.36%) | 80/588 (13.61%) | 0.009(S)~ | 20/231 (8.66%) | 7/57 (12.28%) | 0.401(NS)~ | 10/83 (12.05%) | 4/38 (10.53%) | 1.000(NS)† |
| Negative T waves |  | 637/979 (65.07%) | 380/586 (64.85%) | 0.930(NS)~ | 151/231 (65.37%) | 29/56 (51.79%) | 0.059(NS)~ | 59/83 (71.08%) | 26/38 (68.42%) | 0.766(NS)~ |
| ST depression |  | 321/977 (32.86%) | 214/585 (36.58%) | 0.133(NS)~ | 73/231 (31.60%) | 13/56 (23.21%) | 0.219(NS)~ | 30/83 (36.14%) | 9/38 (23.68%) | 0.173(NS)~ |
| ST elevation |  | 226/978 (23.11%) | 113/585 (19.32%) | 0.078(NS)~ | 59/231 (25.54%) | 6/56 (10.71%) | 0.017(S)~ | 16/83 (19.28%) | 7/38 (18.42%) | 0.911(NS)~ |
| Maximum R in praecordial (mm) | Median (Q1 ; Q3) | 15.0 (10.0;22.0) | 17.0 (11.0;23.0) | 0.009 (S) § | 14.0 (9.0;20.0) | 10.0 (6.0;16.0) | 0.016 (S) § | 19.0 (12.0;24.0) | 23.0 (15.0;27.0) | 0.195 (NS) § |
| Maximum S in praecordial (mm) | Median (Q1 ; Q3) | 17.0 (12.0;23.0) | 15.0 (11.0;21.0) | <0.001 (S) § | 18.0 (12.0;23.0) | 13.0 (11.0;19.0) | 0.015 (S) § | 16.0 (12.0;24.0) | 15.0 (12.0;18.0) | 0.282 (NS) § |
| Maximum R in limbs (mm) | Median (Q1 ; Q3) | 11.0 (8.0;16.0) | 12.0 (8.0;16.0) | 0.195 (NS) § | 11.0 (8.0;15.0) | 9.0 (7.0;12.0) | 0.071 (NS) § | 13.0 (9.0;22.0) | 15.0 (10.0;19.0) | 0.596 (NS) § |
| Maximum S in limbs (mm) | Median (Q1 ; Q3) | 10.0 (6.0;14.0) | 10.0 (6.0;14.0) | 0.756 (NS) § | 9.5 (6.0;13.0) | 8.0 (5.0;12.0) | 0.188 (NS) § | 12.0 (7.0;17.0) | 13.0 (6.0;17.0) | 0.662 (NS) § |
| Preexcitation |  | 18/983 (1.83%) | 3/588 (0.51%) | 0.027(S)~ | 4/231 (1.73%) | 0/57 (0.00%) | 1.000(NS)† | 3/83 (3.61%) | 0/38 (0.00%) | 0.551(NS)† |
| Abn Q-waves |  | 232/983 (23.60%) | 125/588 (21.26%) | 0.284(NS)~ | 65/231 (28.14%) | 12/57 (21.05%) | 0.279(NS)~ | 16/83 (19.28%) | 3/38 (7.89%) | 0.110(NS)~ |
| **Echocardiogram** | | | | | | | | | | |
| LVEDD (mm) | Median (Q1 ; Q3) | 45.0 (41.0;49.0) | 45.0 (41.0;50.0) | 0.224 (NS) § | 44.0 (40.0;48.0) | 45.0 (41.0;50.0) | 0.118 (NS) § | 45.0 (43.0;50.0) | 45.0 (39.0;48.0) | 0.224 (NS) § |
| LVESD (mm) | Median (Q1 ; Q3) | 28.0 (23.0;32.0) | 28.0 (24.0;33.0) | 0.375 (NS) § | 26.0 (22.3;31.0) | 28.0 (26.8;34.0) | 0.010 (S) § | 27.9 (24.0;31.0) | 26.5 (24.0;30.0) | 0.675 (NS) § |
| LV ejection fraction (Simpson>s biplane) (%) | Median (Q1 ; Q3) | 64.0 (58.0;70.0) | 60.0 (55.0;69.0) | 0.020 (S) § | 65.0 (60.0;71.0) | 63.0 (59.0;68.0) | 0.099 (NS) § | 64.0 (57.0;71.5) | 65.0 (60.0;70.0) | 0.438 (NS) § |
| Fractional shortening (%) | Median (Q1 ; Q3) | 39.0 (33.0;44.0) | 37.4 (32.0;44.0) | 0.094 (NS) § | 40.0 (35.0;45.0) | 35.5 (32.1;38.5) | 0.005 (S) § | 40.4 (35.0;46.0) | 40.0 (37.0;48.0) | 0.782 (NS) § |
| LVEDV (LV End Diastolic Volume) (mL) | Median (Q1 ; Q3) | 89.0 (72.0;110.0) | 94.5 (76.0;118.0) | 0.081 (NS) § | 88.0 (71.0;105.0) | 87.0 (68.2;103.0) | 0.929 (NS) § | 93.5 (69.0;110.0) | 97.0 (75.0;112.0) | 0.834 (NS) § |
| LVESV (LV end systolic volume) (mL) | Median (Q1 ; Q3) | 30.0 (22.0;42.0) | 31.5 (23.0;45.0) | 0.352 (NS) § | 30.0 (22.0;40.0) | 30.0 (24.0;36.5) | 0.796 (NS) § | 33.0 (23.0;40.0) | 30.0 (20.0;39.0) | 0.491 (NS) § |
| Maximum LV thickness (mm) | Median (Q1 ; Q3) | 19.0 (16.0;23.0) | 18.9 (16.0;22.0) | 0.234 (NS) § | 20.0 (17.0;22.5) | 18.0 (16.0;22.0) | 0.166 (NS) § | 20.0 (16.0;24.0) | 17.0 (16.0;23.0) | 0.213 (NS) § |
| LV septal thickness diastole (mm) | Median (Q1 ; Q3) | 18.0 (15.0;22.0) | 18.0 (15.0;21.0) | 0.536 (NS) § | 18.5 (15.8;22.0) | 18.0 (16.0;21.5) | 0.469 (NS) § | 19.0 (15.0;22.0) | 17.0 (15.0;23.0) | 0.502 (NS) § |
| LV posterior wall thickness diastole (mm) | Median (Q1 ; Q3) | 11.0 (9.0;13.0) | 12.0 (10.0;14.0) | <0.001 (S) § | 10.0 (9.0;12.0) | 11.0 (9.0;12.6) | 0.221 (NS) § | 11.0 (9.0;13.0) | 12.0 (11.0;13.0) | 0.167 (NS) § |
| Left atrium diameter (mm) | Median (Q1 ; Q3) | 43.0 (38.0;49.0) | 45.0 (40.0;51.0) | <0.001 (S) § | 43.2 (37.0;50.0) | 47.5 (41.5;57.0) | <0.001 (S) § | 44.5 (37.0;50.0) | 45.0 (41.0;50.0) | 0.313 (NS) § |
| Left atrial area (cm²) | Median (Q1 ; Q3) | 24.9 (20.0;31.0) | 27.0 (22.0;33.0) | 0.005 (S) § | 23.0 (20.0;30.0) | 30.0 (22.4;32.0) | 0.174 (NS) § | 25.0 (23.0;30.2) | 23.8 (20.6;28.0) | 0.330 (NS) § |
| Pattern of LV hypertrophy | No hypertrophy | 27/1023 (2.64%) | 13/604 (2.15%) | <0.001(S)~ | 3/248 (1.21%) | 1/57 (1.75%) | 0.731(NS)† | 0/73 (0.00%) | 0/38 (0.00%) | 0.308(NS)† |
|  | Asymmetrical septal | 776/1023 (75.86%) | 391/604 (64.74%) |  | 217/248 (87.50%) | 49/57 (85.96%) |  | 51/73 (69.86%) | 24/38 (63.16%) |  |
|  | Concentric | 108/1023 (10.56%) | 112/604 (18.54%) |  | 10/248 (4.03%) | 4/57 (7.02%) |  | 10/73 (13.70%) | 9/38 (23.68%) |  |
|  | Apical | 76/1023 (7.43%) | 67/604 (11.09%) |  | 9/248 (3.63%) | 2/57 (3.51%) |  | 8/73 (10.96%) | 5/38 (13.16%) |  |
| RV dilation |  | 32/1041 (3.07%) | 23/624 (3.69%) | 0.499(NS)~ | 3/249 (1.20%) | 2/58 (3.45%) | 0.239(NS)† | 3/77 (3.90%) | 1/40 (2.50%) | 1.000(NS)† |
| RV global systolic dysfunction |  | 42/1041 (4.03%) | 24/624 (3.85%) | 0.849(NS)~ | 4/249 (1.61%) | 1/58 (1.72%) | 1.000(NS)† | 3/77 (3.90%) | 3/40 (7.50%) | 0.410(NS)† |
| RV hypertrophy |  | 139/1041 (13.35%) | 104/624 (16.67%) | 0.064(NS)~ | 21/249 (8.43%) | 6/58 (10.34%) | 0.643(NS)~ | 9/77 (11.69%) | 8/40 (20.00%) | 0.226(NS)~ |
| Maximum RV wall thickness (mm) | Median (Q1 ; Q3) | 5.0 (4.0;7.0) | 6.0 (4.0;7.0) | 0.006 (S) § | 5.0 (3.0;6.0) | 4.0 (4.0;7.0) | 0.885 (NS) § | 5.0 (5.0;7.0) | 6.0 (2.0;7.0) | 0.796 (NS) § |
| Mitral E-wave (m/s) | Median (Q1 ; Q3) | 0.7 (0.6;0.9) | 0.7 (0.6;0.9) | 0.186 (NS) § | 0.7 (0.6;0.9) | 0.7 (0.6;0.9) | 0.690 (NS) § | 0.7 (0.6;0.9) | 0.9 (0.7;1.0) | 0.033 (S) § |
| E-wave deceleration time (m/s) | Median (Q1 ; Q3) | 200.0 (161.0;236.0) | 209.0 (169.0;260.0) | 0.008 (S) § | 201.0 (170.0;233.5) | 200.5 (160.0;243.0) | 0.763 (NS) § | 206.0 (175.0;264.0) | 225.0 (190.0;296.0) | 0.217 (NS) § |
| Mitral A-wave (m/s) | Median (Q1 ; Q3) | 0.6 (0.4;0.8) | 0.8 (0.6;1.0) | <0.001 (S) § | 0.5 (0.4;0.7) | 0.7 (0.5;0.8) | 0.002 (S) § | 0.6 (0.5;0.8) | 0.9 (0.6;1.1) | 0.003 (S) § |
| TDI lateral mitral annulus peak E velocity m/s (E or Ea) (m/s) | Median (Q1 ; Q3) | 0.1 (0.1;1.0) | 0.1 (0.1;0.1) | <0.001 (S) § | 0.1 (0.1;0.2) | 0.1 (0.0;0.8) | 0.228 (NS) § | 0.1 (0.1;0.1) | 0.1 (0.1;0.1) | 0.563 (NS) § |
| Diastolic dysfunction (grade) | Nomal | 241/805 (29.94%) | 67/467 (14.35%) | <0.001(S)~ | 58/188 (30.85%) | 9/38 (23.68%) | 0.829(NS)~ | 20/55 (36.36%) | 4/33 (12.12%) | 0.070(NS)† |
|  | Grade I (impaired relaxation) | 281/805 (34.91%) | 255/467 (54.60%) |  | 65/188 (34.57%) | 15/38 (39.47%) |  | 21/55 (38.18%) | 16/33 (48.48%) |  |
|  | Grade II (pseudo normal) | 208/805 (25.84%) | 116/467 (24.84%) |  | 44/188 (23.40%) | 10/38 (26.32%) |  | 11/55 (20.00%) | 9/33 (27.27%) |  |
|  | Restrictive | 75/805 (9.32%) | 29/467 (6.21%) |  | 21/188 (11.17%) | 4/38 (10.53%) |  | 3/55 (5.45%) | 4/33 (12.12%) |  |
| Left ventricular outflow tract gradient (mmHg) | Median (Q1 ; Q3) | 8.4 (4.0;35.0) | 13.0 (6.0;45.0) | 0.004 (S) § | 6.0 (3.0;15.0) | 5.2 (0.0;10.0) | 0.358 (NS) § | 10.0 (5.0;40.0) | 12.0 (6.7;42.0) | 0.729 (NS) § |
| Maximum provoked (by any technique) peak left ventricular outflow tract gradient (mmHg) | Median (Q1 ; Q3) | 10.6 (5.0;40.0) | 20.0 (7.0;70.0) | <0.001 (S) § | 8.0 (4.0;30.0) | 5.2 (1.5;18.0) | 0.323 (NS) § | 23.0 (6.0;58.0) | 19.0 (8.2;60.0) | 0.713 (NS) § |
| Aortic regurgitation | None | 785/964 (81.43%) | 387/578 (66.96%) | <0.001(S)† | 205/235 (87.23%) | 36/55 (65.45%) | <0.001(S)† | 56/73 (76.71%) | 25/38 (65.79%) | 0.368(NS)† |
|  | Mild | 159/964 (16.49%) | 165/578 (28.55%) |  | 24/235 (10.21%) | 16/55 (29.09%) |  | 13/73 (17.81%) | 9/38 (23.68%) |  |
|  | Moderate | 18/964 (1.87%) | 25/578 (4.33%) |  | 5/235 (2.13%) | 3/55 (5.45%) |  | 3/73 (4.11%) | 4/38 (10.53%) |  |
|  | Severe | 2/964 (0.21%) | 1/578 (0.17%) |  | 1/235 (0.43%) | 0/55 (0.00%) |  | 1/73 (1.37%) | 0/38 (0.00%) |  |
| Mitral regurgitation | None | 280/964 (29.05%) | 120/578 (20.76%) | <0.001(S)~ | 85/235 (36.17%) | 18/55 (32.73%) | 0.098(NS)† | 28/73 (38.36%) | 13/38 (34.21%) | 0.457(NS)† |
|  | Mild | 544/964 (56.43%) | 327/578 (56.57%) |  | 124/235 (52.77%) | 26/55 (47.27%) |  | 28/73 (38.36%) | 20/38 (52.63%) |  |
|  | Moderate | 119/964 (12.34%) | 118/578 (20.42%) |  | 24/235 (10.21%) | 8/55 (14.55%) |  | 15/73 (20.55%) | 5/38 (13.16%) |  |
|  | Severe | 21/964 (2.18%) | 13/578 (2.25%) |  | 2/235 (0.85%) | 3/55 (5.45%) |  | 2/73 (2.74%) | 0/38 (0.00%) |  |
| Systolic Pulmonary Artery pressure (mmHg) | Median (Q1 ; Q3) | 30.0 (24.0;37.0) | 34.0 (25.0;40.0) | 0.005 (S) § | 27.0 (20.0;40.0) | 35.0 (30.5;48.5) | 0.030 (S) § | 39.0 (32.0;49.0) | 38.0 (35.0;45.0) | 0.785 (NS) § |
| **Cardiac Magnetic Resonance Imaging** | | | | | | | | | | |
| Late gadolinium enhancement |  | 300/391 (76.73%) | 134/197 (68.02%) | 0.037(S)~ | 66/90 (73.33%) | 11/15 (73.33%) | 0.899(NS)† | 11/19 (57.89%) | 7/11 (63.64%) | 1.000(NS)† |
| LV end-diastolic volume (mL) | Median (Q1 ; Q3) | 134.2 (103.5;163.0) | 136.0 (109.0;171.0) | 0.341 (NS) § | 127.0 (97.0;159.0) | 114.4 (90.0;160.6) | 0.738 (NS) § | 146.0 (110.0;154.0) | 136.5 (113.0;167.0) | 0.781 (NS) § |
| LV end-systolic volume (mL) | Median (Q1 ; Q3) | 42.0 (29.0;57.0) | 42.5 (30.0;59.0) | 0.531 (NS) § | 39.0 (29.0;57.0) | 35.0 (28.0;53.0) | 0.743 (NS) § | 49.0 (30.0;63.0) | 34.5 (29.0;53.0) | 0.470 (NS) § |
| LV ejection fraction (%) | Median (Q1 ; Q3) | 67.3 (61.0;74.1) | 69.0 (60.0;74.0) | 0.724 (NS) § | 67.1 (60.0;73.4) | 66.7 (64.4;72.0) | 0.827 (NS) § | 65.6 (55.5;78.5) | 71.5 (69.0;76.0) | 0.316 (NS) § |
|  | Median (Q1 ; Q3) | 67.3 (61.0;74.1) | 69.0 (60.0;74.0) |  | 67.1 (60.0;73.4) | 66.7 (64.4;72.0) |  | 65.6 (55.5;78.5) | 71.5 (69.0;76.0) |  |
| Maximum LV thickness by CMR (mm) | Median (Q1 ; Q3) | 20.0 (16.0;24.0) | 19.0 (17.0;22.0) | 0.467 (NS) § | 20.0 (17.0;25.0) | 18.0 (16.0;22.0) | 0.390 (NS) § | 19.0 (15.0;23.0) | 20.0 (17.0;22.0) | 0.400 (NS) § |
| Pattern of hypertrophy | Septal | 122/385 (31.69%) | 67/194 (34.54%) | 0.350(NS)~ | 28/90 (31.11%) | 4/15 (26.67%) | 0.638(NS)† | 9/19 (47.37%) | 5/11 (45.45%) | 0.754(NS)† |
|  | Concentric | 178/385 (46.23%) | 73/194 (37.63%) |  | 49/90 (54.44%) | 9/15 (60.00%) |  | 5/19 (26.32%) | 4/11 (36.36%) |  |
|  | Eccentric | 17/385 (4.42%) | 14/194 (7.22%) |  | 1/90 (1.11%) | 1/15 (6.67%) |  | 1/19 (5.26%) | 0/11 (0.00%) |  |
|  | Apical | 25/385 (6.49%) | 14/194 (7.22%) |  | 6/90 (6.67%) | 1/15 (6.67%) |  | 4/19 (21.05%) | 1/11 (9.09%) |  |
| **Holter** | | | | | | | | | | |
| Non-sustained ventricular tachycardia |  | 160/729 (21.95%) | 77/430 (17.91%) | 0.099(NS)~ | 38/175 (21.71%) | 22/46 (47.83%) | <0.001(S)~ | 10/52 (19.23%) | 5/23 (21.74%) | 0.765(NS)† |
| Rhythm : atrial fibrillation throughout + paroxysmal atrial fibrillation in sinus rhythm |  | 62/730 (8.49%) | 66/432 (15.28%) | <0.001(S)~ | 10/175 (5.71%) | 12/46 (26.09%) | <0.001(S)† | 6/52 (11.54%) | 2/23 (8.70%) | 1.000(NS)† |
| Rhythm : Conduction defect |  | 5/730 (0.68%) | 8/432 (1.85%) | 0.084(NS)† | 1/175 (0.57%) | 1/46 (2.17%) | 0.374(NS)† | 0/52 (0.00%) | 0/23 (0.00%) | NA |
| **Exercise test** | | | | | | | | | | |
| Absolute workload achieved (Watts)* | Median (Q1 ; Q3) | 120.0 (95.0;166.0) | 111.0 (76.8;150.0) | 0.192 (NS) § | 119.0 (100.0;175.0) | 119.5 (58.0;150.0) | 0.539 (NS) § | 142.0 (122.0;216.0) | 150.0 (135.0;150.0) | 0.806 (NS) § |
| Absolute workload achieved (METS) | Median (Q1 ; Q3) | 8.3 (6.0;11.3) | 7.0 (5.1;8.2) | 0.002 (S) § | 9.8 (6.8;12.0) | 7.0 (5.5;8.9) | 0.135 (NS) § | 8.0 (5.6;10.9) | 8.1 (6.0;9.5) | 1.000 (NS) § |
| Max VO2 achieved (ml/min/Kg) | Median (Q1 ; Q3) | 21.0 (17.5;27.1) | 17.1 (14.0;20.5) | <0.001 (S) § | 23.6 (18.1;29.1) | 15.1 (10.0;18.6) | 0.006 (S) § | 23.6 (18.1;29.1) | 15.1 (10.0;18.6) | 0.513 (NS) § |
| % of maximum estimated VO2 achieved Bicycle | Median (Q1 ; Q3) | 29.3 (12.2;35.6) | 27.8 (9.7;32.2) | 0.027 (S) § | 31.5 (14.0;36.7) | 28.1 (6.8;31.1) | 0.082 (NS) § | 29.1 (19.9;33.5) | 28.9 (22.2;32.2) | 0.673 (NS) § |
| % of maximum estimated VO2 achieved Treadmill | Median (Q1 ; Q3) | 38.4 (33.1;44.1) | 31.1 (24.8;37.8) | <0.001 (S) § | 38.6 (34.2;44.8) | 31.0 (21.2;41.6) | 0.055 (NS) § | 37.4 (29.5;40.8) | 24.9 (24.1;25.8) | 0.040 (S) § |
| Ventricular arrhythmia |  | 18/486 (3.70%) | 11/200 (5.50%) | 0.288(NS)~ | 3/117 (2.56%) | 2/23 (8.70%) | 0.189(NS)† | 0/23 (0.00%) | 0/15 (0.00%) | NA |
| Supraventricular arrhythmia |  | 18/487 (3.70%) | 6/200 (3.00%) | 0.652(NS)~ | 5/117 (4.27%) | 2/23 (8.70%) | 0.323(NS)† | 1/23 (4.35%) | 1/15 (6.67%) | 1.000(NS)† |
| **Laboratory** | | | | | | | | | | |
| NT-proBNP (pg/mL) | Median (Q1 ; Q3) | 853.0 (280.0;1853.0) | 941.3 (388.0;2300.0) | 0.336 (NS) § | 810.0 (318.0;1813.6) | 1331.0 (715.0;2700.0) | 0.147 (NS) § | 1038.5 (467.0;2565.0) | 731.0 (440.7;880.0) | 0.803 (NS) § |
| BNP (pg/mL) | Median (Q1 ; Q3) | 210.0 (90.0;494.0) | 220.0 (122.0;432.5) | 0.678 (NS) § | 120.0 (70.0;227.5) | 390.0 (177.0;587.0) | 0.193 (NS) § | 205.5 (155.0;530.0) | 107.0 (27.0;231.0) | 0.121 (NS) § |
| **Medications** | | | | | | | | | | |
| Alpha-blockers |  | 13/918 (1.42%) | 36/639 (5.63%) | <0.001(S)~ | 2/219 (0.91%) | 3/60 (5.00%) | 0.068(NS)† | 1/69 (1.45%) | 1/44 (2.27%) | 1.000(NS)† |
| Calcium antagonists |  | 95/920 (10.33%) | 170/643 (26.44%) | <0.001(S)~ | 25/219 (11.42%) | 15/61 (24.59%) | 0.009(S)~ | 7/69 (10.14%) | 14/44 (31.82%) | 0.004(S)~ |
| ACE-inhibitors |  | 110/920 (11.96%) | 232/643 (36.08%) | <0.001(S)~ | 23/219 (10.50%) | 23/61 (37.70%) | <0.001(S)~ | 11/69 (15.94%) | 14/44 (31.82%) | 0.047(S)~ |
| Angiotensin II receptor blockers |  | 67/920 (7.28%) | 198/643 (30.79%) | <0.001(S)~ | 11/219 (5.02%) | 17/61 (27.87%) | <0.001(S)~ | 8/69 (11.59%) | 14/44 (31.82%) | 0.008(S)~ |
| ACE inhibitors/angiotensin II receptor blockers |  | 174/920 (18.91%) | 427/642 (66.51%) | <0.001(S)~ | 34/219 (15.53%) | 40/61 (65.57%) | <0.001(S)~ | 19/69 (27.54%) | 28/44 (63.64%) | <0.001(S)~ |
| Mineralocorticoid receptor antagonists |  | 120/920 (13.04%) | 113/643 (17.57%) | 0.013(S)~ | 29/219 (13.24%) | 16/61 (26.23%) | 0.015(S)~ | 4/69 (5.80%) | 3/44 (6.82%) | 1.000(NS)† |
| Antiplatelets |  | 187/920 (20.33%) | 233/643 (36.24%) | <0.001(S)~ | 33/219 (15.07%) | 13/61 (21.31%) | 0.245(NS)~ | 19/69 (27.54%) | 16/44 (36.36%) | 0.322(NS)~ |
| Oral anticoagulants |  | 235/920 (25.54%) | 189/643 (29.39%) | 0.092(NS)~ | 58/219 (26.48%) | 28/61 (45.90%) | 0.004(S)~ | 14/69 (20.29%) | 11/44 (25.00%) | 0.556(NS)~ |
| Amiodarone |  | 89/848 (10.50%) | 73/613 (11.91%) | 0.396(NS)~ | 24/204 (11.76%) | 7/60 (11.67%) | 0.983(NS)~ | 8/62 (12.90%) | 3/41 (7.32%) | 0.519(NS)† |
| **Device therapy** | | | | | | | | | | |
| Cardioverter defibrillator implanted |  | 264/1091 (24.20%) | 82/648 (12.65%) | <0.001(S)~ | 92/257 (35.80%) | 17/62 (27.42%) | 0.212(NS)~ | 22/87 (25.29%) | 5/44 (11.36%) | 0.063(NS)~ |
| Reason for cardioverter defibrillator | Primary prophylaxis | 223/264 (84.47%) | 74/82 (90.24%) | 0.190(NS)~ | 82/92 (89.13%) | 16/17 (94.12%) | 1.000(NS)† | 19/22 (86.36%) | 3/5 (60.00%) | 0.221(NS)† |
|  | Secondary prophylaxis | 41/264 (15.53%) | 8/82 (9.76%) |  | 10/92 (10.87%) | 1/17 (5.88%) |  | 3/22 (13.64%) | 2/5 (40.00%) |  |
| Pacemaker implanted |  | 85/1078 (7.88%) | 50/645 (7.75%) | 0.921(NS)~ | 17/254 (6.69%) | 5/61 (8.20%) | 0.779(NS)† | 9/87 (10.34%) | 7/44 (15.91%) | 0.358(NS)~ |
| Reason for pacemaker: Brady |  | 51/92 (55.43%) | 36/51 (70.59%) | 0.075(NS)~ | 10/19 (52.63%) | 4/6 (66.67%) | 0.661(NS)† | 7/9 (77.78%) | 4/7 (57.14%) | 0.596(NS)† |
| Reason for pacemaker: Treatment of left ventricular outflow tract obstruction |  | 18/89 (20.22%) | 10/52 (19.23%) | 0.886(NS)~ | 4/18 (22.22%) | 0/5 (0.00%) | 0.539(NS)† | 0/9 (0.00%) | 1/7 (14.29%) | 0.438(NS)† |
| Reason for pacemaker: Cardiac resynchronization therapy |  | 14/91 (15.38%) | 2/50 (4.00%) | 0.041(S)~ | 5/18 (27.78%) | 0/5 (0.00%) | 0.545(NS)† | 0/9 (0.00%) | 1/7 (14.29%) | 0.438(NS)† |
| **Other procedures** | | | | | | | | | | |
| Septal myectomy |  | 54/1091 (4.95%) | 31/648 (4.78%) | 0.877(NS)~ | 16/257 (6.23%) | 4/62 (6.45%) | 1.000(NS)† | 1/87 (1.15%) | 2/44 (4.55%) | 0.261(NS)† |
| Alcohol septal ablation |  | 43/1091 (3.94%) | 27/648 (4.17%) | 0.817(NS)~ | 6/257 (2.33%) | 2/62 (3.23%) | 0.655(NS)† | 4/87 (4.60%) | 1/44 (2.27%) | 0.663(NS)† |

**Legend:** § : Kruskal-Wallis test ; † : Exact-Fisher test ; ~ : Chi-square test ; NC : Not calculable. All continuous variables are presented as Median (Q1 ; Q3) and categorical variables as N and percentage. HCM : hypertrophic cardiomyopathy ; SCD : sudden cardiac death; NYHA : New York Heart Association ; BBB : bundle branch block ; LBBB : left bundle branch block ; LVEDD : left ventricular end-diastolic dimension ; LVESD : left ventricular end-systolic dimension ; LV : left ventricle ; RV : right ventricle ; VO2 : oxygen consumption.

#

# **Supplemental table 2. Comparison between diabetic and non-diabetic patients.**

|  | | **All** | | | **Genotype-positive** | | | **Genotype-negative** | | |
| --- | --- | --- | --- | --- | --- | --- | --- | --- | --- | --- |
| **Variable** | **Modality** | **Diabetes (N=176)** | **No diabetes (N=1563)** | **P-value** | **Diabetes (N=20)** | **No diabetes (N=299)** | **P-value** | **Diabetes (N=14)** | **No diabetes (N=117)** | **P-value** |
| **Demographic variables and co-morbidities** | | | | | | | | | | |
| Age at first evaluation in the centre (years) | Median (Q1 ; Q3) | 60.0 (53.0;69.0) | 49.0 (36.0;60.0) | <0.001 **(S)** § | 56.0 (50.0;69.0) | 42.0 (29.0;53.0) | <0.001 **(S)** § | 60.0 (52.0;68.0) | 51.0 (40.0;62.0) | 0.028 **(S)** § |
| Age at enrolment | Median (Q1 ; Q3) | 65.0 (57.0;72.0) | 54.0 (41.0;64.0) | <0.001 **(S)** § | 65.0 (55.0;73.5) | 49.0 (37.0;61.0) | <0.001 **(S)** § | 67.0 (61.0;74.0) | 57.0 (45.0;69.0) | 0.010 **(S)** § |
| Gender | Female | 78/176 (44.32%) | 633/1563 (40.50%) | 0.329(NS)~ | 12/20 (60.00%) | 135/299 (45.15%) | 0.197(NS)~ | 3/14 (21.43%) | 39/117 (33.33%) | 0.547(NS)† |
|  | Male | 98/176 (55.68%) | 930/1563 (59.50%) |  | 8/20 (40.00%) | 164/299 (54.85%) |  | 11/14 (78.57%) | 78/117 (66.67%) |  |
| Body Mass Index (kg/m²) | Median (Q1 ; Q3) | 28.9 (26.1;31.1) | 26.2 (23.5;29.3) | <0.001 **(S)** § | 29.7 (27.5;32.4) | 25.8 (23.2;28.4) | <0.001 **(S)** § | 29.9 (24.7;32.6) | 26.5 (24.1;30.0) | 0.199 (NS) § |
| Family history of HCM |  | 44/146 (30.14%) | 617/1318 (46.81%) | <0.001**(S)**~ | 13/19 (68.42%) | 217/281 (77.22%) | 0.298(NS)† | 3/13 (23.08%) | 41/105 (39.05%) | 0.323(NS)† |
| Family history of SCD |  | 24/167 (14.37%) | 326/1495 (21.81%) | 0.025**(S)**~ | 8/19 (42.11%) | 96/297 (32.32%) | 0.379(NS)~ | 1/13 (7.69%) | 26/112 (23.21%) | 0.295(NS)† |
| Hypertension |  | 122/176 (69.32%) | 526/1563 (33.65%) | <0.001**(S)**~ | 9/20 (45.00%) | 53/299 (17.73%) | 0.006**(S)**† | 7/14 (50.00%) | 37/117 (31.62%) | 0.231(NS)† |
| Hyperlipidaemia/dyslipidaemia |  | 108/176 (61.36%) | 527/1563 (33.72%) | <0.001**(S)**~ | 11/20 (55.00%) | 76/299 (25.42%) | 0.004**(S)**~ | 9/14 (64.29%) | 41/117 (35.04%) | 0.033**(S)**~ |
| Physical activity |  | 51/139 (36.69%) | 621/1227 (50.61%) | 0.002**(S)**~ | 5/16 (31.25%) | 111/253 (43.87%) | 0.323(NS)~ | 6/10 (60.00%) | 40/76 (52.63%) | 0.745(NS)† |
| Smoking |  | 61/161 (37.89%) | 440/1425 (30.88%) | 0.070(NS)~ | 3/16 (18.75%) | 86/263 (32.70%) | 0.245(NS)~ | 7/12 (58.33%) | 18/90 (20.00%) | 0.008**(S)**† |
| Renal impairment |  | 38/176 (21.59%) | 120/1563 (7.68%) | <0.001**(S)**~ | 4/20 (20.00%) | 10/299 (3.34%) | 0.008**(S)**† | 2/14 (14.29%) | 15/117 (12.82%) | 1.000(NS)† |
| Anaemia |  | 22/172 (12.79%) | 58/1545 (3.75%) | <0.001**(S)**~ | 1/19 (5.26%) | 5/294 (1.70%) | 0.315(NS)† | 1/14 (7.14%) | 5/117 (4.27%) | 0.500(NS)† |
| Chronic obstructive pulmonary disease |  | 19/176 (10.80%) | 48/1563 (3.07%) | <0.001**(S)**~ | 0/20 (0.00%) | 8/299 (2.68%) | 1.000(NS)† | 4/14 (28.57%) | 3/117 (2.56%) | 0.002**(S)**† |
|  |  |  |  |  |  |  |  |  |  |  |

|  | | **All** | | | **Genotype-positive** | | | **Genotype-negative** | | |
| --- | --- | --- | --- | --- | --- | --- | --- | --- | --- | --- |
| **Variable** | **Modality** | **Diabetes (N=176)** | **No diabetes (N=1563)** | **P-value** | **Diabetes (N=20)** | **No diabetes (N=299)** | **P-value** | **Diabetes (N=14)** | **No diabetes (N=117)** | **P-value** |
| **Symptoms** | | | | | | | | | | |
| Age at first symptom (years) | Median (Q1 ; Q3) | 54.0 (42.0;66.0) | 43.0 (29.0;55.0) | <0.001 **(S)** § | 38.0 (34.0;55.0) | 36.0 (24.0;49.0) | 0.222 (NS) § | 60.0 (49.0;63.0) | 50.0 (29.0;58.0) | 0.142 (NS) § |
| Unexplained syncope (suspected arrhythmic cardiogenic+mechanism uncertain) |  | 26/156 (16.67%) | 237/1297 (18.27%) | 0.623(NS)~ | 4/19 (21.05%) | 48/248 (19.35%) | 0.771(NS)† | 2/12 (16.67%) | 13/85 (15.29%) | 1.000(NS)† |
| Anginal chest pain |  | 60/158 (37.97%) | 453/1312 (34.53%) | 0.390(NS)~ | 6/19 (31.58%) | 61/249 (24.50%) | 0.582(NS)† | 3/12 (25.00%) | 28/86 (32.56%) | 0.747(NS)† |
| NYHA class | NYHA I | 30/154 (19.48%) | 433/1259 (34.39%) | <0.001**(S)**~ | 4/19 (21.05%) | 96/243 (39.51%) | 0.160(NS)~ | 2/11 (18.18%) | 26/85 (30.59%) | 0.434(NS)† |
|  | NYHA II | 82/154 (53.25%) | 623/1259 (49.48%) |  | 10/19 (52.63%) | 114/243 (46.91%) |  | 5/11 (45.45%) | 43/85 (50.59%) |  |
|  | NYHA III | 38/154 (24.68%) | 188/1259 (14.93%) |  | 5/19 (26.32%) | 33/243 (13.58%) |  | 4/11 (36.36%) | 15/85 (17.65%) |  |
|  | NYHA IV | 4/154 (2.60%) | 15/1259 (1.19%) |  | 0/19 (0.00%) | 0/243 (0.00%) |  | 0/11 (0.00%) | 1/85 (1.18%) |  |
| NYHA functional class > II |  | 42/154 (27.27%) | 203/1259 (16.12%) | <0.001**(S)**~ | 5/19 (26.32%) | 33/243 (13.58%) | 0.167(NS)† | 4/11 (36.36%) | 16/85 (18.82%) | 0.233(NS)† |
| Palpitations |  | 41/158 (25.95%) | 506/1312 (38.57%) | 0.002**(S)**~ | 2/19 (10.53%) | 98/249 (39.36%) | 0.012**(S)**~ | 2/12 (16.67%) | 41/86 (47.67%) | 0.043**(S)**~ |
| Orthopnea |  | 15/158 (9.49%) | 95/1312 (7.24%) | 0.309(NS)~ | 3/19 (15.79%) | 11/249 (4.42%) | 0.067(NS)† | 0/12 (0.00%) | 8/86 (9.30%) | 0.590(NS)† |
| Ankle oedema |  | 16/158 (10.13%) | 91/1312 (6.94%) | 0.145(NS)~ | 0/19 (0.00%) | 13/249 (5.22%) | 0.608(NS)† | 1/12 (8.33%) | 3/86 (3.49%) | 0.412(NS)† |
| Paroxysmal nocturnal dyspnea |  | 12/158 (7.59%) | 62/1312 (4.73%) | 0.119(NS)~ | 0/19 (0.00%) | 10/249 (4.02%) | 1.000(NS)† | 1/12 (8.33%) | 4/86 (4.65%) | 0.487(NS)† |
| **Arrhythmia history** | | | | | | | | | | |
| History of Atrial Fibrillation |  | 71/176 (40.34%) | 392/1563 (25.08%) | <0.001**(S)**~ | 11/20 (55.00%) | 78/299 (26.09%) | 0.005**(S)**~ | 5/14 (35.71%) | 30/117 (25.64%) | 0.523(NS)† |
| History of stroke | No | 160/176 (90.91%) | 1468/1563 (93.92%) | 0.146(NS)† | 16/20 (80.00%) | 277/299 (92.64%) | 0.020**(S)**† | 13/14 (92.86%) | 110/117 (94.02%) | 0.606(NS)† |
|  | TIA | 5/176 (2.84%) | 36/1563 (2.30%) |  | 0/20 (0.00%) | 10/299 (3.34%) |  | 0/14 (0.00%) | 2/117 (1.71%) |  |
|  | Stroke | 8/176 (4.55%) | 51/1563 (3.26%) |  | 2/20 (10.00%) | 9/299 (3.01%) |  | 1/14 (7.14%) | 4/117 (3.42%) |  |
| Sustained ventricular tachycardia |  | 5/176 (2.84%) | 129/1563 (8.25%) | 0.011**(S)**~ | 1/20 (5.00%) | 31/299 (10.37%) | 0.706(NS)† | 1/14 (7.14%) | 7/117 (5.98%) | 1.000(NS)† |
| Resuscitated ventricular fibrillation/cardiac arrest |  | 1/176 (0.57%) | 48/1563 (3.07%) | 0.055(NS)† | 1/20 (5.00%) | 15/299 (5.02%) | 1.000(NS)† | 0/14 (0.00%) | 3/117 (2.56%) | 1.000(NS)† |
| History of BBB |  | 15/110 (13.64%) | 137/948 (14.45%) | 0.818(NS)~ | 3/10 (30.00%) | 19/164 (11.59%) | 0.117(NS)† | 1/12 (8.33%) | 21/96 (21.88%) | 0.453(NS)† |
| **ECG** | | | | | | | | | | |
| Rhythm : Atrial fibrillation and Atrial flutter |  | 35/173 (20.23%) | 112/1511 (7.41%) | <0.001**(S)**~ | 8/20 (40.00%) | 18/292 (6.16%) | <0.001**(S)**† | 4/14 (28.57%) | 6/117 (5.13%) | 0.012**(S)**† |
| QT interval (ms) | Median (Q1 ; Q3) | 418.0 (390.0;452.0) | 430.0 (400.0;458.0) | 0.094 (NS) § | 437.0 (400.0;451.0) | 430.0 (400.0;454.0) | 0.903 (NS) § | 400.0 (390.0;448.0) | 436.0 (410.0;476.0) | 0.068 (NS) § |
| PR interval (ms) | Median (Q1 ; Q3) | 180.0 (160.0;203.0) | 164.0 (146.0;190.0) | <0.001 **(S)** § | 189.0 (160.0;200.0) | 164.0 (146.0;190.0) | 0.105 (NS) § | 196.5 (176.5;207.0) | 170.0 (144.0;200.0) | 0.087 (NS) § |
| QRS duration (ms) | Median (Q1 ; Q3) | 98.0 (88.0;112.0) | Median (Q1 ; Q3) | 0.421 (NS) § | 98.0 (85.0;104.0) | 99.5 (88.0;112.0) | 0.214 (NS) § | 96.0 (90.0;106.0) | 100.0 (92.0;121.5) | 0.269 (NS) § |
| QRS axis (degrees) | Median (Q1 ; Q3) | 32.0 (14.5;46.5) | 37.0 (19.0;60.0) | 0.149 (NS) § | 30.0 (10.5;45.0) | 34.0 (15.0;60.0) | 0.214 (NS) § | 30.0 (13.0;40.0) | 33.0 (18.0;58.0) | 0.318 (NS) § |
| Atrioventricular block : 1st degree |  | 27/173 (15.61%) | 150/1508 (9.95%) | 0.022**(S)**~ | 3/20 (15.00%) | 30/292 (10.27%) | 0.455(NS)† | 4/14 (28.57%) | 14/117 (11.97%) | 0.103(NS)† |
| Bundle branch block : Incomplete LBBB+LBBB |  | 19/162 (11.73%) | 153/1409 (10.86%) | 0.737(NS)~ | 2/20 (10.00%) | 25/268 (9.33%) | 1.000(NS)† | 1/13 (7.69%) | 13/108 (12.04%) | 1.000(NS)† |
| Negative T waves |  | 98/162 (60.49%) | 919/1403 (65.50%) | 0.206(NS)~ | 10/20 (50.00%) | 170/267 (63.67%) | 0.223(NS)~ | 10/13 (76.92%) | 75/108 (69.44%) | 0.753(NS)† |
| ST depression |  | 57/162 (35.19%) | 478/1400 (34.14%) | 0.791(NS)~ | 6/20 (30.00%) | 80/267 (29.96%) | 0.997(NS)~ | 4/13 (30.77%) | 35/108 (32.41%) | 1.000(NS)† |
| ST elevation |  | 25/162 (15.43%) | 314/1401 (22.41%) | 0.041**(S)**~ | 2/20 (10.00%) | 63/267 (23.60%) | 0.265(NS)† | 0/13 (0.00%) | 23/108 (21.30%) | 0.126(NS)† |
| Maximum R in praecordial (mm) | Median (Q1 ; Q3) | 15.0 (10.0;21.0) | 16.0 (10.0;22.0) | 0.162 (NS) § | 10.5 (8.0;14.0) | 14.0 (9.0;19.5) | 0.145 (NS) § | 22.0 (11.5;27.5) | 20.0 (12.0;25.0) | 0.622 (NS) § |
| Maximum S in praecordial (mm) | Median (Q1 ; Q3) | 13.0 (10.0;19.0) | 16.0 (12.0;22.0) | <0.001 **(S)** § | 19.5 (9.5;24.0) | 17.0 (12.0;23.0) | 0.809 (NS) § | 13.0 (9.5;17.0) | 16.0 (12.0;24.0) | 0.135 (NS) § |
| Maximum R in limbs (mm) | Median (Q1 ; Q3) | 10.0 (8.0;14.0) | 12.0 (8.0;16.0) | 0.053 (NS) § | 9.5 (5.5;15.0) | 10.5 (8.0;15.0) | 0.445 (NS) § | 14.5 (5.5;17.0) | 14.0 (9.0;20.0) | 0.414 (NS) § |
| Maximum S in limbs (mm) | Median (Q1 ; Q3) | 8.0 (6.0;12.0) | 10.0 (6.0;14.0) | 0.062 (NS) § | 9.5 (5.0;12.5) | 9.0 (6.0;13.0) | 0.997 (NS) § | 10.0 (5.5;13.0) | 12.0 (7.0;17.0) | 0.231 (NS) § |
| Preexcitation |  | 0/162 (0.00%) | 21/1409 (1.49%) | 0.157(NS)† | 0/20 (0.00%) | 4/268 (1.49%) | 1.000(NS)† | 0/13 (0.00%) | 3/108 (2.78%) | 1.000(NS)† |
| Abn Q-waves |  | 33/162 (20.37%) | 324/1409 (23.00%) | 0.450(NS)~ | 3/20 (15.00%) | 74/268 (27.61%) | 0.219(NS)~ | 0/13 (0.00%) | 19/108 (17.59%) | 0.218(NS)† |
| **Echocardiogram** | | | | | | | | | | |
| LVEDD (mm) | Median (Q1 ; Q3) | 46.0 (41.0;50.0) | 45.0 (41.0;50.0) | 0.570 (NS) § | 44.2 (39.0;49.5) | 45.0 (41.0;49.0) | 0.764 (NS) § | 47.5 (45.0;52.0) | 45.0 (42.0;50.0) | 0.282 (NS) § |
| LVESD (mm) | Median (Q1 ; Q3) | 27.5 (23.7;33.0) | 28.0 (23.0;32.0) | 0.597 (NS) § | 28.0 (22.9;32.0) | 26.9 (23.0;32.0) | 0.464 (NS) § | 29.0 (24.0;31.0) | 27.0 (24.0;30.0) | 0.716 (NS) § |
| LV ejection fraction (Simpson>s biplane) (%) | Median (Q1 ; Q3) | 63.0 (58.0;69.0) | 63.0 (57.0;70.0) | 0.783 (NS) § | 61.5 (53.0;67.0) | 65.0 (60.0;70.0) | 0.103 (NS) § | 67.5 (60.0;72.0) | 64.5 (58.0;71.0) | 0.377 (NS) § |
| Fractional shortening (%) | Median (Q1 ; Q3) | 36.0 (32.0;43.0) | 39.0 (32.3;44.0) | 0.205 (NS) § | 13 (7) | 172 (115) | 0.158 (NS) § | 45.5 (41.2;50.5) | 39.8 (35.0;46.5) | 0.111 (NS) § |
| LVEDV (LV End Diastolic Volume) (mL) | Median (Q1 ; Q3) | 86.0 (67.0;110.0) | 91.0 (74.0;112.0) | 0.139 (NS) § | 79.5 (60.0;101.5) | 88.0 (71.0;105.0) | 0.189 (NS) § | 68.5 (51.5;88.0) | 94.0 (72.0;113.0) | 0.102 (NS) § |
| LVESV (LV end systolic volume) (mL) | Median (Q1 ; Q3) | 31.5 (22.0;45.8) | 30.8 (22.0;43.0) | 0.790 (NS) § | 26.0 (22.0;44.0) | 30.0 (22.0;40.0) | 0.646 (NS) § | 24.0 (15.5;28.0) | 33.0 (23.0;40.0) | 0.071 (NS) § |
| Maximum LV thickness (mm) | Median (Q1 ; Q3) | 18.0 (16.0;22.0) | 19.0 (16.0;22.0) | 0.287 (NS) § | 19.5 (17.0;23.5) | 19.0 (17.0;22.0) | 0.505 (NS) § | 17.0 (15.5;21.5) | 20.0 (16.0;24.0) | 0.145 (NS) § |
| LV septal thickness diastole (mm) | Median (Q1 ; Q3) | 18.0 (15.0;21.0) | 18.0 (15.0;22.0) | 0.234 (NS) § | 18.5 (17.0;21.5) | 18.0 (15.3;22.0) | 0.498 (NS) § | 16.5 (14.5;21.5) | 18.0 (15.5;23.0) | 0.264 (NS) § |
| LV posterior wall thickness diastole (mm) | Median (Q1 ; Q3) | 12.0 (10.0;14.0) | 11.0 (10.0;13.0) | 0.054 (NS) § | 10.9 (9.0;12.0) | 10.0 (9.0;12.0) | 0.454 (NS) § | 13.0 (10.0;14.0) | 11.0 (10.0;13.0) | 0.270 (NS) § |
| Left atrium diameter (mm) | Median (Q1 ; Q3) | 44.0 (40.0;51.0) | 44.0 (38.0;50.0) | 0.058 (NS) § | 45.5 (41.0;57.0) | 45.0 (37.5;51.0) | 0.377 (NS) § | 46.0 (41.5;54.5) | 45.0 (38.0;50.0) | 0.306 (NS) § |
| Left atrial area (cm²) | Median (Q1 ; Q3) | 25.3 (20.5;34.0) | 26.0 (20.6;32.0) | 0.830 (NS) § | 25.5 (21.0;30.0) | 23.2 (20.0;30.0) | 0.815 (NS) § | 21.0 (20.9;24.5) | 25.0 (23.0;29.6) | 0.216 (NS) § |
| Pattern of LV hypertrophy | No hypertrophy | 4/168 (2.38%) | 36/1459 (2.47%) | 0.705(NS)~ | 0/20 (0.00%) | 4/285 (1.40%) | 1.000(NS)† | 0/13 (0.00%) | 0/98 (0.00%) | 0.024**(S)**† |
|  | Asymmetrical septal | 117/168 (69.64%) | 1050/1459 (71.97%) |  | 19/20 (95.00%) | 247/285 (86.67%) |  | 6/13 (46.15%) | 69/98 (70.41%) |  |
|  | Concentric | 28/168 (16.67%) | 192/1459 (13.16%) |  | 1/20 (5.00%) | 13/285 (4.56%) |  | 2/13 (15.38%) | 17/98 (17.35%) |  |
|  | Apical | 15/168 (8.93%) | 128/1459 (8.77%) |  | 0/20 (0.00%) | 11/285 (3.86%) |  | 5/13 (38.46%) | 8/98 (8.16%) |  |
| RV dilation |  | 5/174 (2.87%) | 50/1491 (3.35%) | 0.737(NS)~ | 0/20 (0.00%) | 5/287 (1.74%) | 1.000(NS)† | 1/14 (7.14%) | 3/103 (2.91%) | 0.404(NS)† |
| RV global systolic dysfunction |  | 7/174 (4.02%) | 59/1491 (3.96%) | 0.966(NS)~ | 1/20 (5.00%) | 4/287 (1.39%) | 0.288(NS)† | 2/14 (14.29%) | 4/103 (3.88%) | 0.151(NS)† |
| RV hypertrophy |  | 26/174 (14.94%) | 217/1491 (14.55%) | 0.891(NS)~ | 2/20 (10.00%) | 25/287 (8.71%) | 0.692(NS)† | 4/14 (28.57%) | 13/103 (12.62%) | 0.121(NS)† |
| Maximum RV wall thickness (mm) | Median (Q1 ; Q3) | 5.3 (4.0;7.0) | 5.9 (4.0;7.0) | 0.349 (NS) § | 5.0 (5.0;5.0) | 4.6 (3.0;6.8) | 0.809 (NS) § | 6.0 (6.0;6.0) | 5.0 (4.8;7.0) | 0.785 (NS) § |
| Mitral E-wave (m/s) | Median (Q1 ; Q3) | 0.7 (0.6;1.0) | 0.7 (0.6;0.9) | 0.609 (NS) § | 0.8 (0.7;1.1) | 0.7 (0.6;0.9) | 0.031 **(S)** § | 0.6 (0.5;0.9) | 0.8 (0.6;0.9) | 0.351 (NS) § |
| E-wave deceleration time (m/s) | Median (Q1 ; Q3) | 225.0 (190.0;269.0) | 200.0 (161.0;242.0) | 0.003 **(S)** § | 209.5 (160.0;271.0) | 200.0 (170.0;238.5) | 0.874 (NS) § | 227.5 (205.0;300.0) | 206.0 (180.0;270.0) | 0.369 (NS) § |
| Mitral A-wave (m/s) | Median (Q1 ; Q3) | 0.8 (0.6;1.0) | 0.6 (0.5;0.8) | <0.001 **(S)** § | 0.8 (0.5;1.0) | 0.6 (0.4;0.7) | 0.048 **(S)** § | 0.7 (0.6;1.0) | 0.7 (0.5;0.9) | 0.375 (NS) § |
| TDI lateral mitral annulus peak E velocity m/s (E or Ea) (m/s) | Median (Q1 ; Q3) | 0.1 (0.1;0.1) | 0.1 (0.1;0.6) | <0.001 **(S)** § | 0.1 (0.0;0.1) | 0.1 (0.1;0.3) | 0.012 **(S)** § | 0.1 (0.1;0.1) | 0.1 (0.1;0.1) | 0.704 (NS) § |
| Diastolic dysfunction (grade) | Nomal | 19/124 (15.32%) | 289/1148 (25.17%) | 0.003**(S)**~ | 2/11 (18.18%) | 65/215 (30.23%) | 0.059(NS)† | 0/9 (0.00%) | 24/79 (30.38%) | 0.081(NS)† |
|  | Grade I (impaired relaxation) | 71/124 (57.26%) | 465/1148 (40.51%) |  | 2/11 (18.18%) | 78/215 (36.28%) |  | 5/9 (55.56%) | 32/79 (40.51%) |  |
|  | Grade II (pseudo normal) | 24/124 (19.35%) | 300/1148 (26.13%) |  | 3/11 (27.27%) | 51/215 (23.72%) |  | 2/9 (22.22%) | 18/79 (22.78%) |  |
|  | Restrictive | 10/124 (8.06%) | 94/1148 (8.19%) |  | 4/11 (36.36%) | 21/215 (9.77%) |  | 2/9 (22.22%) | 5/79 (6.33%) |  |
| Left ventricular outflow tract gradient (mmHg) | Median (Q1 ; Q3) | 12.0 (6.0;34.0) | 10.0 (5.0;40.0) | 0.428 (NS) § | 6.3 (0.0;45.0) | 6.0 (2.0;14.5) | 0.742 (NS) § | 9.0 (5.0;15.0) | 14.0 (6.0;42.0) | 0.363 (NS) § |
|  | Median (Q1 ; Q3) | 12.0 (6.0;34.0) | 10.0 (5.0;40.0) |  | 6.3 (0.0;45.0) | 6.0 (2.0;14.5) |  | 9.0 (5.0;15.0) | 14.0 (6.0;42.0) |  |
| Maximum provoked (by any technique) peak left ventricular outflow tract gradient (mmHg) | Median (Q1 ; Q3) | 15.5 (8.0;58.0) | 13.5 (5.7;50.0) | 0.207 (NS) § | 6.3 (6.3;6.3) | 8.0 (3.8;28.0) | 0.781 (NS) § | 12.0 (4.0;40.0) | 21.0 (7.0;60.0) | 0.279 (NS) § |
| Aortic regurgitation | None | 117/160 (73.13%) | 1055/1382 (76.34%) | 0.633(NS)† | 14/19 (73.68%) | 227/271 (83.76%) | 0.285(NS)† | 9/14 (64.29%) | 72/97 (74.23%) | 0.579(NS)† |
|  | Mild | 37/160 (23.13%) | 287/1382 (20.77%) |  | 5/19 (26.32%) | 35/271 (12.92%) |  | 4/14 (28.57%) | 18/97 (18.56%) |  |
|  | Moderate | 6/160 (3.75%) | 37/1382 (2.68%) |  | 0/19 (0.00%) | 8/271 (2.95%) |  | 1/14 (7.14%) | 6/97 (6.19%) |  |
|  | Severe | 0/160 (0.00%) | 3/1382 (0.22%) |  | 0/19 (0.00%) | 1/271 (0.37%) |  | 0/14 (0.00%) | 1/97 (1.03%) |  |
| Mitral regurgitation | None | 39/160 (24.38%) | 361/1382 (26.12%) | 0.520(NS)~ | 4/19 (21.05%) | 99/271 (36.53%) | 0.514(NS)† | 3/14 (21.43%) | 38/97 (39.18%) | 0.292(NS)† |
|  | Mild | 87/160 (54.38%) | 784/1382 (56.73%) |  | 12/19 (63.16%) | 138/271 (50.92%) |  | 6/14 (42.86%) | 42/97 (43.30%) |  |
|  | Moderate | 31/160 (19.38%) | 206/1382 (14.91%) |  | 3/19 (15.79%) | 29/271 (10.70%) |  | 5/14 (35.71%) | 15/97 (15.46%) |  |
|  | Severe | 3/160 (1.88%) | 31/1382 (2.24%) |  | 0/19 (0.00%) | 5/271 (1.85%) |  | 0/14 (0.00%) | 2/97 (2.06%) |  |
| Systolic Pulmonary Artery pressure (mmHg) | Median (Q1 ; Q3) | 35.0 (28.0;49.0) | 30.0 (24.0;39.0) | 0.015 **(S)** § | 37.0 (35.0;50.0) | 28.0 (21.0;39.5) | 0.024 **(S)** § | 49.0 (35.0;65.0) | 38.0 (31.0;45.0) | 0.118 (NS) § |
| **Cardiac Magnetic Resonance Imaging** | | | | | | | | | | |
| Late gadolinium enhancement |  | 41/60 (68.33%) | 393/528 (74.43%) | 0.221(NS)~ | 4/5 (80.00%) | 73/100 (73.00%) | 0.543(NS)† | 2/3 (66.67%) | 16/27 (59.26%) | 1.000(NS)† |
|  | No | 17/60 (28.33%) | 103/528 (19.51%) |  | 0/5 (0.00%) | 18/100 (18.00%) |  | 1/3 (33.33%) | 11/27 (40.74%) |  |
| LV end-diastolic volume (mL) | Median (Q1 ; Q3) | 126.5 (93.0;141.5) | 137.0 (108.0;169.0) | 0.008 **(S)** § | 132.0 (95.0;132.0) | 127.0 (95.0;160.6) | 0.646 (NS) § | 124.0 (94.0;154.0) | 140.0 (113.0;165.0) | 0.548 (NS) § |
| LV end-systolic volume (mL) | Median (Q1 ; Q3) | 38.0 (25.0;50.0) | 42.3 (30.0;59.5) | 0.058 (NS) § | 38.0 (28.0;48.0) | 39.0 (29.0;58.0) | 0.639 (NS) § | 56.0 (43.0;69.0) | 36.0 (29.0;58.0) | 0.367 (NS) § |
| LV ejection fraction (%) | Median (Q1 ; Q3) | 68.0 (60.0;74.3) | 68.0 (60.6;74.0) | 0.969 (NS) § | 66.0 (64.0;74.3) | 67.1 (61.3;73.0) | 0.760 (NS) § | 54.5 (54.0;55.0) | 70.0 (60.5;78.0) | 0.083 (NS) § |
| Maximum LV thickness by CMR (mm) | Median (Q1 ; Q3) | 19.0 (17.0;22.0) | 20.0 (16.0;24.0) | 0.351 (NS) § | 20.5 (16.0;25.5) | 20.0 (17.0;25.0) | 0.871 (NS) § | 19.0 (15.0;20.0) | 20.0 (16.0;23.0) | 0.488 (NS) § |
| Pattern of hypertrophy | Septal | 18/58 (31.03%) | 171/521 (32.82%) | NC | 2/5 (40.00%) | 30/100 (30.00%) | 1.000(NS)† | 1/3 (33.33%) | 13/27 (48.15%) | 0.238(NS)† |
|  | Concentric | 24/58 (41.38%) | 227/521 (43.57%) |  | 3/5 (60.00%) | 55/100 (55.00%) |  | 1/3 (33.33%) | 8/27 (29.63%) |  |
|  | Eccentric | 6/58 (10.34%) | 25/521 (4.80%) |  | 0/5 (0.00%) | 2/100 (2.00%) |  | 0/3 (0.00%) | 1/27 (3.70%) |  |
|  | Apical | 4/58 (6.90%) | 35/521 (6.72%) |  | 0/5 (0.00%) | 7/100 (7.00%) |  | 0/3 (0.00%) | 5/27 (18.52%) |  |
| **Holter** | | | | | | | | | | |
| Non-sustained ventricular tachycardia |  | 24/123 (19.51%) | 213/1036 (20.56%) | 0.785(NS)~ | 5/16 (31.25%) | 55/205 (26.83%) | 0.771(NS)† | 2/8 (25.00%) | 13/67 (19.40%) | 0.657(NS)† |
| Rhythm : atrial fibrillation throughout + paroxysmal atrial fibrillation in sinus rhythm |  | 25/123 (20.33%) | 103/1039 (9.91%) | <0.001**(S)**~ | 7/16 (43.75%) | 15/205 (7.32%) | <0.001**(S)**† | 0/8 (0.00%) | 8/67 (11.94%) | 0.588(NS)† |
| Rhythm : Conduction defect |  | 3/123 (2.44%) | 10/1039 (0.96%) | 0.151(NS)† | 0/16 (0.00%) | 2/205 (0.98%) | 1.000(NS)† | 0/8 (0.00%) | 0/67 (0.00%) | NA |
| **Exercise test** | | | | | | | | | | |
| Absolute workload achieved (Watts)* | Median (Q1 ; Q3) | 120.0 (60.0;140.0) | 119.0 (94.0;158.0) | 0.149 (NS) § | 110.0 (100.0;120.0) | 119.0 (97.0;175.0) | 0.773 (NS) § | 105.0 (60.0;150.0) | 150.0 (127.0;164.0) | 0.303 (NS) § |
| Absolute workload achieved (METS) | Median (Q1 ; Q3) | 6.2 (5.0;7.0) | 7.7 (5.7;10.7) | 0.004 **(S)** § | 6.0 (4.1;7.0) | 9.7 (6.8;11.6) | 0.077 (NS) § | . (.;.) | 8.1 (5.7;9.7) |  |
| Max VO2 achieved (ml/min/Kg) | Median (Q1 ; Q3) | 16.6 (13.8;21.0) | 20.2 (16.9;25.9) | 0.014 **(S)** § | 27.6 (27.6;27.6) | 23.0 (17.8;28.3) | 0.431 (NS) § | 12.2 (12.2;12.2) | 22.8 (18.1;24.9) | 0.143 (NS) § |
| % of maximum estimated VO2 achieved Bicycle | Median (Q1 ; Q3) | 28.9 (9.7;31.9) | 28.7 (12.1;34.8) | 0.302 (NS) § | 19.6 (8.1;31.1) | 31.5 (13.8;36.3) | 0.286 (NS) § | 29.3 (27.0;33.4) | 28.5 (16.0;32.2) | 0.580 (NS) § |
| % of maximum estimated VO2 achieved Treadmill | Median (Q1 ; Q3) | 30.4 (24.0;36.1) | 37.8 (31.5;43.1) | 0.002 **(S)** § | 23.4 (21.9;26.7) | 39.3 (34.0;44.8) | 0.004 **(S)** § | . (.;.) | 32.5 (26.4;38.8) |  |
| Ventricular arrhythmia |  | 1/55 (1.82%) | 28/631 (4.44%) | 0.722(NS)† | 0/6 (0.00%) | 5/134 (3.73%) | 1.000(NS)† | 0/5 (0.00%) | 0/33 (0.00%) | NA |
| Supraventricular arrhythmia |  | 2/55 (3.64%) | 22/632 (3.48%) | 1.000(NS)† | 1/6 (16.67%) | 6/134 (4.48%) | 0.269(NS)† | 0/5 (0.00%) | 2/33 (6.06%) | 1.000(NS)† |
| **Laboratory** | | | | | | | | | | |
| NT-proBNP (pg/mL) | Median (Q1 ; Q3) | 1067.0 (469.8;4514.5) | 854.4 (325.0;1888.0) | 0.167 (NS) § | 802.5 (615.0;2182.0) | 861.0 (343.0;1888.0) | 0.639 (NS) § | 824.3 (440.7;1208.0) | 869.0 (467.0;2565.0) | 0.643 (NS) § |
| BNP (pg/mL) | Median (Q1 ; Q3) | 200.0 (93.0;391.0) | 213.0 (97.0;494.0) | 0.550 (NS) § | . (.;.) | 120.0 (70.0;429.0) |  | 27.0 (27.0;27.0) | 202.5 (139.5;383.5) | 0.121 (NS) § |
| **Medications** | | | | | | | | | | |
| Alpha-blockers |  | 8/174 (4.60%) | 41/1383 (2.96%) | 0.245(NS)~ | 0/20 (0.00%) | 5/259 (1.93%) | 1.000(NS)† | 0/14 (0.00%) | 2/99 (2.02%) | 1.000(NS)† |
| Calcium antagonists |  | 46/174 (26.44%) | 219/1389 (15.77%) | <0.001**(S)**~ | 6/20 (30.00%) | 34/260 (13.08%) | 0.048**(S)**† | 5/14 (35.71%) | 16/99 (16.16%) | 0.133(NS)† |
| ACE-inhibitors |  | 65/174 (37.36%) | 277/1389 (19.94%) | <0.001**(S)**~ | 7/20 (35.00%) | 39/260 (15.00%) | 0.029**(S)**† | 6/14 (42.86%) | 19/99 (19.19%) | 0.079(NS)† |
| Angiotensin II receptor blockers |  | 39/174 (22.41%) | 226/1389 (16.27%) | 0.042**(S)**~ | 3/20 (15.00%) | 25/260 (9.62%) | 0.434(NS)† | 2/14 (14.29%) | 20/99 (20.20%) | 1.000(NS)† |
| ACE inhibitors/angiotensin II receptor blockers |  | 104/174 (59.77%) | 497/1388 (35.81%) | <0.001**(S)**~ | 10/20 (50.00%) | 64/260 (24.62%) | 0.013**(S)**~ | 8/14 (57.14%) | 39/99 (39.39%) | 0.207(NS)~ |
| Mineralocorticoid receptor antagonists |  | 35/174 (20.11%) | 198/1389 (14.25%) | 0.041**(S)**~ | 5/20 (25.00%) | 40/260 (15.38%) | 0.337(NS)† | 3/14 (21.43%) | 4/99 (4.04%) | 0.040**(S)**† |
| Antiplatelets |  | 80/174 (45.98%) | 340/1389 (24.48%) | <0.001**(S)**~ | 4/20 (20.00%) | 42/260 (16.15%) | 0.753(NS)† | 7/14 (50.00%) | 28/99 (28.28%) | 0.125(NS)† |
| Oral anticoagulants |  | 54/174 (31.03%) | 370/1389 (26.64%) | 0.219(NS)~ | 11/20 (55.00%) | 75/260 (28.85%) | 0.015**(S)**~ | 2/14 (14.29%) | 23/99 (23.23%) | 0.732(NS)† |
| Amiodarone |  | 21/165 (12.73%) | 141/1296 (10.88%) | 0.477(NS)~ | 1/19 (5.26%) | 30/245 (12.24%) | 0.709(NS)† | 0/14 (0.00%) | 11/89 (12.36%) | 0.353(NS)† |
| **Device therapy** | | | | | | | | | | |
| Cardioverter defibrillator implanted |  | 22/176 (12.50%) | 324/1563 (20.73%) | 0.010**(S)**~ | 5/20 (25.00%) | 104/299 (34.78%) | 0.372(NS)~ | 2/14 (14.29%) | 25/117 (21.37%) | 0.733(NS)† |
| Reason for cardioverter defibrillator | Primary prophylaxis | 20/22 (90.91%) | 277/324 (85.49%) | 0.752(NS)† | 5/5 (100.00%) | 93/104 (89.42%) | 1.000(NS)† | 1/2 (50.00%) | 21/25 (84.00%) | 0.342(NS)† |
|  | Secondary prophylaxis | 2/22 (9.09%) | 47/324 (14.51%) |  | 0/5 (0.00%) | 11/104 (10.58%) |  | 1/2 (50.00%) | 4/25 (16.00%) |  |
| Pacemaker implanted |  | 14/175 (8.00%) | 121/1548 (7.82%) | 0.932(NS)~ | 0/19 (0.00%) | 22/296 (7.43%) | 0.380(NS)† | 3/14 (21.43%) | 13/117 (11.11%) | 0.378(NS)† |
| Reason for pacemaker: Brady |  | 9/15 (60.00%) | 78/128 (60.94%) | 0.944(NS)~ | 1/1 (100.00%) | 13/24 (54.17%) | 1.000(NS)† | 2/3 (66.67%) | 9/13 (69.23%) | 1.000(NS)† |
| Reason for pacemaker: Treatment of left ventricular outflow tract obstruction |  | 3/14 (21.43%) | 25/127 (19.69%) | 1.000(NS)† | 0/0 (0.00%) | 4/23 (17.39%) | ..~ | 0/3 (0.00%) | 1/13 (7.69%) | 1.000(NS)† |
| Reason for pacemaker: Cardiac resynchronization therapy |  | 1/14 (7.14%) | 15/127 (11.81%) | 1.000(NS)† | 0/0 (0.00%) | 5/23 (21.74%) | ..~ | 1/3 (33.33%) | 0/13 (0.00%) | 0.188(NS)† |
| **Other procedures** | | | | | | | | | | |
| Septal myectomy |  | 10/176 (5.68%) | 75/1563 (4.80%) | 0.606(NS)~ | 2/20 (10.00%) | 18/299 (6.02%) | 0.362(NS)† | 0/14 (0.00%) | 3/117 (2.56%) | 1.000(NS)† |
| Alcohol septal ablation |  | 7/176 (3.98%) | 63/1563 (4.03%) | 0.973(NS)~ | 0/20 (0.00%) | 8/299 (2.68%) | 1.000(NS)† | 0/14 (0.00%) | 5/117 (4.27%) | 1.000(NS)† |

**Legend:** § : Kruskal-Wallis test ; † : Exact-Fisher test ; ~ : Chi-square test ; NC : Not calculable. All continuous variables are presented as Median (Q1 ; Q3) and categorical variables as N and percentage. HCM : hypertrophic cardiomyopathy ; SCD : sudden cardiac death; NYHA : New York Heart Association ; BBB : bundle branch block ; LBBB : left bundle branch block ; LVEDD : left ventricular end-diastolic dimension ; LVESD : left ventricular end-systolic dimension ; LV : left ventricle ; RV : right ventricle ; VO2 : oxygen consumption.

# **Supplemental table 3. Comparison between obese and non-obese patients**

|  | | **All** | | | **Genotype-positive** | | | **Genotype-negative** | | |
| --- | --- | --- | --- | --- | --- | --- | --- | --- | --- | --- |
| **Variable** | **Modality** | **Obese (N=360)** | **Not obese (N=1245)** | **P-value** | **Obese (N=54)** | **Not obese (N=246)** | **P-value** | **Obese (N=35)** | **Not obese (N=89)** | **P-value** |
| **Demographic variables and co-morbidities** | | | | | | | | | | |
| Age at first evaluation in the centre (years) | Median (Q1 ; Q3) | 53.0 (44.0;62.0) | 49.0 (35.0;61.0) | <0.001 **(S)** § | 48.0 (40.0;57.0) | 42.0 (28.0;53.0) | 0.008 **(S)** § | 52.5 (47.0;58.0) | 53.0 (31.0;66.0) | 0.701 (NS) § |
| Age at enrolment | Median (Q1 ; Q3) | 58.0 (48.5;66.0) | 54.0 (40.0;65.0) | <0.001 **(S)** § | 55.0 (44.0;64.0) | 49.0 (37.0;61.0) | 0.025 **(S)** § | 58.0 (50.0;66.0) | 58.0 (43.0;70.0) | 0.667 (NS) § |
| Gender | Female | 156/360 (43.33%) | 506/1245 (40.64%) | 0.361(NS)~ | 28/54 (51.85%) | 111/246 (45.12%) | 0.369(NS)~ | 11/35 (31.43%) | 28/89 (31.46%) | 0.997(NS)~ |
|  | Male | 204/360 (56.67%) | 739/1245 (59.36%) |  | 26/54 (48.15%) | 135/246 (54.88%) |  | 24/35 (68.57%) | 61/89 (68.54%) |  |
| Body Mass Index (kg/m²) | Median (Q1 ; Q3) | 32.7 (31.0;35.2) | 25.4 (23.1;27.3) | <0.001 **(S)** § | 32.4 (30.8;35.7) | 25.0 (22.9;27.1) | <0.001 **(S)** § | 32.5 (30.9;34.9) | 25.2 (23.1;27.4) | <0.001 **(S)** § |
| Family history of HCM |  | 127/302 (42.05%) | 505/1078 (46.85%) | 0.190(NS)~ | 41/53 (77.36%) | 180/229 (78.60%) | 0.005**(S)**~ | 11/30 (36.67%) | 29/81 (35.80%) | 0.033**(S)**~ |
| Family history of SCD |  | 79/346 (22.83%) | 256/1196 (21.40%) | 0.571(NS)~ | 21/53 (39.62%) | 79/244 (32.38%) | 0.312(NS)~ | 5/32 (15.63%) | 20/86 (23.26%) | 0.367(NS)~ |
| Hypertension |  | 194/360 (53.89%) | 393/1245 (31.57%) | <0.001**(S)**~ | 17/54 (31.48%) | 38/246 (15.45%) | 0.006**(S)**~ | 17/35 (48.57%) | 24/89 (26.97%) | 0.021**(S)**~ |
| Diabetes mellitus II |  | 57/360 (15.83%) | 92/1245 (7.39%) | <0.001**(S)**~ | 8/54 (14.81%) | 8/246 (3.25%) | 0.003**(S)**† | 6/35 (17.14%) | 6/89 (6.74%) | 0.096(NS)† |
| Hyperlipidaemia/dyslipidaemia |  | 191/360 (53.06%) | 384/1245 (30.84%) | <0.001**(S)**~ | 24/54 (44.44%) | 58/246 (23.58%) | 0.002**(S)**~ | 15/35 (42.86%) | 30/89 (33.71%) | 0.340(NS)~ |
| Physical activity |  | 111/266 (41.73%) | 536/1007 (53.23%) | <0.001**(S)**~ | 17/45 (37.78%) | 92/211 (43.60%) | 0.473(NS)~ | 11/19 (57.89%) | 33/63 (52.38%) | 0.673(NS)~ |
| Smoking |  | 118/329 (35.87%) | 343/1142 (30.04%) | 0.045**(S)**~ | 16/46 (34.78%) | 67/215 (31.16%) | 0.632(NS)~ | 11/27 (40.74%) | 12/68 (17.65%) | 0.018**(S)**~ |
| Renal impairment |  | 37/360 (10.28%) | 103/1245 (8.27%) | 0.235(NS)~ | 3/54 (5.56%) | 9/246 (3.66%) | 0.458(NS)† | 6/35 (17.14%) | 10/89 (11.24%) | 0.384(NS)† |
| Anaemia |  | 11/357 (3.08%) | 60/1228 (4.89%) | 0.147(NS)~ | 0/53 (0.00%) | 6/241 (2.49%) | 0.596(NS)† | 1/35 (2.86%) | 4/89 (4.49%) | 1.000(NS)† |
| Chronic obstructive pulmonary disease |  | 19/360 (5.28%) | 42/1245 (3.37%) | 0.096(NS)~ | 2/54 (3.70%) | 5/246 (2.03%) | 0.613(NS)† | 1/35 (2.86%) | 5/89 (5.62%) | 1.000(NS)† |

#

|  | | **All** | | | **Genotype-positive** | | | **Genotype-negative** | | |
| --- | --- | --- | --- | --- | --- | --- | --- | --- | --- | --- |
| **Variable** | **Modality** | **Obese (N=360)** | **Not obese (N=1245)** | **P-value** | **Obese (N=54)** | **Not obese (N=246)** | **P-value** | **Obese (N=35)** | **Not obese (N=89)** | **P-value** |
| **Symptoms** | | | | | | | | | | |
| Age at first symptom (years) | Median (Q1 ; Q3) | 49.0 (38.0;57.5) | 43.0 (29.0;57.0) | 0.015 **(S)** § | 42.5 (32.5;51.5) | 35.0 (23.0;49.0) | 0.019 **(S)** § | 53.0 (44.0;57.0) | 47.5 (27.5;60.0) | 0.666 (NS) § |
| Unexplained syncope (suspected arrhythmic cardiogenic+mechanism uncertain) |  | 57/316 (18.04%) | 190/1042 (18.23%) | 0.937(NS)~ | 9/46 (19.57%) | 40/207 (19.32%) | 0.970(NS)~ | 3/25 (12.00%) | 11/67 (16.42%) | 0.751(NS)† |
| Anginal chest pain |  | 128/318 (40.25%) | 351/1054 (33.30%) | 0.023**(S)**~ | 14/46 (30.43%) | 49/208 (23.56%) | 0.328(NS)~ | 7/25 (28.00%) | 23/68 (33.82%) | 0.594(NS)~ |
| NYHA class | NYHA I | 71/309 (22.98%) | 367/1010 (36.34%) | <0.001**(S)**~ | 8/45 (17.78%) | 85/203 (41.87%) | 0.009**(S)**~ | 5/23 (21.74%) | 21/68 (30.88%) | 0.387(NS)† |
|  | NYHA II | 167/309 (54.05%) | 487/1010 (48.22%) |  | 27/45 (60.00%) | 91/203 (44.83%) |  | 11/23 (47.83%) | 36/68 (52.94%) |  |
|  | NYHA III | 68/309 (22.01%) | 140/1010 (13.86%) |  | 10/45 (22.22%) | 27/203 (13.30%) |  | 7/23 (30.43%) | 10/68 (14.71%) |  |
|  | NYHA IV | 3/309 (0.97%) | 16/1010 (1.58%) |  | 0/45 (0.00%) | 0/203 (0.00%) |  | 0/23 (0.00%) | 1/68 (1.47%) |  |
| NYHA functional class > II |  | 71/309 (22.98%) | 156/1010 (15.45%) | 0.002**(S)**~ | 10/45 (22.22%) | 27/203 (13.30%) | 0.129(NS)~ | 7/23 (30.43%) | 11/68 (16.18%) | 0.224(NS)† |
| Palpitations |  | 110/318 (34.59%) | 402/1054 (38.14%) | 0.251(NS)~ | 12/46 (26.09%) | 82/208 (39.42%) | 0.090(NS)~ | 11/25 (44.00%) | 31/68 (45.59%) | 0.891(NS)~ |
| Orthopnea |  | 26/318 (8.18%) | 80/1054 (7.59%) | 0.732(NS)~ | 2/46 (4.35%) | 11/208 (5.29%) | 1.000(NS)† | 4/25 (16.00%) | 4/68 (5.88%) | 0.204(NS)† |
| Ankle oedema |  | 30/318 (9.43%) | 70/1054 (6.64%) | 0.093(NS)~ | 1/46 (2.17%) | 11/208 (5.29%) | 0.700(NS)† | 1/25 (4.00%) | 3/68 (4.41%) | 1.000(NS)† |
| Paroxysmal nocturnal dyspnea |  | 20/318 (6.29%) | 51/1054 (4.84%) | 0.306(NS)~ | 1/46 (2.17%) | 9/208 (4.33%) | 0.695(NS)† | 2/25 (8.00%) | 3/68 (4.41%) | 0.608(NS)† |
| **Arrhythmia history** |  |  |  |  |  |  |  |  |  |  |
| History of Atrial Fibrillation |  | 111/360 (30.83%) | 313/1245 (25.14%) | 0.031**(S)**~ | 19/54 (35.19%) | 66/246 (26.83%) | 0.217(NS)~ | 9/35 (25.71%) | 24/89 (26.97%) | 0.887(NS)~ |
| History of stroke | No | 336/360 (93.33%) | 1164/1245 (93.49%) | 0.814(NS)~ | 48/54 (88.89%) | 227/246 (92.28%) | 0.217(NS)† | 34/35 (97.14%) | 83/89 (93.26%) | 1.000(NS)† |
|  | TIA | 7/360 (1.94%) | 32/1245 (2.57%) |  | 1/54 (1.85%) | 9/246 (3.66%) |  | 0/35 (0.00%) | 2/89 (2.25%) |  |
|  | Stroke | 15/360 (4.17%) | 42/1245 (3.37%) |  | 3/54 (5.56%) | 8/246 (3.25%) |  | 1/35 (2.86%) | 3/89 (3.37%) |  |
| Sustained ventricular tachycardia |  | 28/360 (7.78%) | 95/1245 (7.63%) | 0.926(NS)~ | 6/54 (11.11%) | 25/246 (10.16%) | 0.836(NS)~ | 2/35 (5.71%) | 6/89 (6.74%) | 1.000(NS)† |
| Resuscitated ventricular fibrillation/cardiac arrest |  | 11/360 (3.06%) | 35/1245 (2.81%) | 0.807(NS)~ | 4/54 (7.41%) | 12/246 (4.88%) | 0.501(NS)† | 1/35 (2.86%) | 2/89 (2.25%) | 1.000(NS)† |
| History of BBB |  | 39/228 (17.11%) | 104/740 (14.05%) | 0.256(NS)~ | 7/32 (21.88%) | 13/127 (10.24%) | 0.131(NS)† | 5/30 (16.67%) | 16/71 (22.54%) | 0.507(NS)~ |
| **ECG** | | | | | | | | | | |
| Rhythm : Atrial fibrillation and Atrial flutter |  | 40/355 (11.27%) | 93/1226 (7.59%) | 0.028**(S)**~ | 7/53 (13.21%) | 16/242 (6.61%) | 0.151(NS)† | 3/35 (8.57%) | 6/89 (6.74%) | 0.711(NS)† |
| QT interval (ms) | Median (Q1 ; Q3) | 436.0 (401.0;462.0) | 426.0 (400.0;456.0) | 0.003 **(S)** § | 440.0 (401.0;458.0) | 430.0 (400.0;452.0) | 0.721 (NS) § | 443.0 (424.0;498.0) | 432.0 (400.0;474.0) | 0.078 (NS) § |
| PR interval (ms) | Median (Q1 ; Q3) | 173.0 (156.0;197.0) | 162.0 (145.0;190.0) | 0.001 **(S)** § | 184.0 (160.0;196.0) | 162.0 (145.0;190.0) | 0.017 **(S)** § | 170.0 (156.0;197.0) | 171.0 (148.0;200.0) | 0.951 (NS) § |
| QRS duration (ms) | Median (Q1 ; Q3) | 100.0 (90.0;120.0) | 98.0 (88.0;112.0) | 0.002 **(S)** § | 98.0 (90.0;110.0) | 98.0 (88.0;112.0) | 0.677 (NS) § | 100.0 (93.0;122.0) | 100.0 (90.0;120.5) | 0.945 (NS) § |
| QRS axis (degrees) | Median (Q1 ; Q3) | 30.0 (13.0;48.0) | 39.0 (20.0;60.0) | <0.001 **(S)** § | 30.0 (14.0;46.0) | 37.5 (15.0;60.0) | 0.134 (NS) § | 19.5 (13.0;42.0) | 39.0 (21.0;55.5) | 0.045 **(S)** § |
| Atrioventricular block : 1st degree |  | 46/355 (12.96%) | 122/1223 (9.98%) | 0.109(NS)~ | 6/53 (11.32%) | 24/242 (9.92%) | 0.759(NS)~ | 6/35 (17.14%) | 10/89 (11.24%) | 0.384(NS)† |
| Bundle branch block : Incomplete LBBB+LBBB |  | 44/324 (13.58%) | 115/1150 (10.00%) | 0.067(NS)~ | 7/47 (14.89%) | 18/224 (8.04%) | 0.164(NS)† | 3/29 (10.34%) | 11/85 (12.94%) | 1.000(NS)† |
| Negative T waves |  | 220/324 (67.90%) | 738/1145 (64.45%) | 0.250(NS)~ | 28/47 (59.57%) | 144/223 (64.57%) | 0.517(NS)~ | 26/29 (89.66%) | 55/85 (64.71%) | 0.011**(S)**~ |
| ST depression |  | 110/324 (33.95%) | 400/1141 (35.06%) | 0.712(NS)~ | 13/47 (27.66%) | 70/223 (31.39%) | 0.614(NS)~ | 8/29 (27.59%) | 30/85 (35.29%) | 0.447(NS)~ |
| ST elevation |  | 70/324 (21.60%) | 256/1143 (22.40%) | 0.762(NS)~ | 7/47 (14.89%) | 55/223 (24.66%) | 0.148(NS)~ | 5/29 (17.24%) | 18/85 (21.18%) | 0.648(NS)~ |
| Maximum R in praecordial (mm) | Median (Q1 ; Q3) | 14.0 (9.0;20.0) | 16.0 (10.0;23.0) | 0.002 **(S)** § | 10.0 (6.0;16.0) | 14.0 (10.0;20.0) | 0.002 **(S)** § | 20.0 (15.0;23.0) | 20.0 (10.0;27.5) | 0.873 (NS) § |
| Maximum S in praecordial (mm) | Median (Q1 ; Q3) | 16.0 (10.0;21.0) | 16.0 (12.0;22.0) | 0.187 (NS) § | 14.5 (10.0;20.5) | 17.0 (12.0;24.0) | 0.041 **(S)** § | 16.0 (10.0;19.0) | 15.0 (13.0;24.0) | 0.153 (NS) § |
| Maximum R in limbs (mm) | Median (Q1 ; Q3) | 12.0 (9.0;17.0) | 11.0 (8.0;16.0) | 0.065 (NS) § | 10.0 (8.0;15.0) | 10.0 (8.0;15.0) | 0.878 (NS) § | 15.0 (12.0;20.0) | 13.5 (8.0;20.0) | 0.211 (NS) § |
| Maximum S in limbs (mm) | Median (Q1 ; Q3) | 10.0 (7.0;14.0) | 10.0 (6.0;14.0) | 0.190 (NS) § | 9.5 (6.0;13.5) | 9.0 (5.5;13.0) | 0.902 (NS) § | 13.0 (8.0;17.0) | 11.5 (7.0;18.0) | 0.977 (NS) § |
| Preexcitation |  | 6/324 (1.85%) | 15/1150 (1.30%) | 0.433(NS)† | 0/47 (0.00%) | 4/224 (1.79%) | 1.000(NS)† | 2/29 (6.90%) | 1/85 (1.18%) | 0.159(NS)† |
| Abn Q-waves |  | 63/324 (19.44%) | 286/1150 (24.87%) | 0.042**(S)**~ | 14/47 (29.79%) | 61/224 (27.23%) | 0.722(NS)~ | 4/29 (13.79%) | 14/85 (16.47%) | 1.000(NS)† |
| **Echocardiogram** | | | | | | | | | | |
| LVEDD (mm) | Median (Q1 ; Q3) | 47.0 (43.0;51.0) | 45.0 (40.2;49.0) | <0.001 **(S)** § | 46.0 (42.0;50.0) | 44.0 (40.2;48.0) | 0.038 **(S)** § | 48.0 (43.0;51.5) | 45.0 (41.0;49.0) | 0.081 (NS) § |
| LVESD (mm) | Median (Q1 ; Q3) | 29.0 (25.0;33.0) | 27.0 (23.0;32.0) | <0.001 **(S)** § | 30.0 (27.0;35.0) | 26.0 (22.0;31.0) | <0.001 **(S)** § | 28.0 (25.0;30.0) | 27.0 (23.0;31.0) | 0.331 (NS) § |
| LV ejection fraction (Simpson>s biplane) (%) | Median (Q1 ; Q3) | 61.0 (58.0;70.0) | 63.0 (57.0;70.0) | 0.764 (NS) § | 64.0 (58.0;70.0) | 66.0 (60.0;70.0) | 0.421 (NS) § | 65.0 (60.0;70.0) | 64.0 (56.0;72.0) | 0.699 (NS) § |
| Fractional shortening (%) | Median (Q1 ; Q3) | 38.0 (32.0;44.0) | 39.0 (33.0;44.0) | 0.387 (NS) § | 36.0 (31.0;39.0) | 39.5 (34.6;45.0) | 0.005 **(S)** § | 42.5 (39.0;49.0) | 39.3 (33.3;47.0) | 0.155 (NS) § |
| LVEDV (LV End Diastolic Volume) (mL) | Median (Q1 ; Q3) | 97.0 (77.6;123.9) | 90.0 (72.0;109.0) | 0.004 **(S)** § | 87.0 (71.0;103.8) | 88.0 (71.0;105.0) | 0.723 (NS) § | 100.0 (72.0;110.0) | 94.0 (76.0;112.0) | 0.952 (NS) § |
| LVESV (LV end systolic volume) (mL) | Median (Q1 ; Q3) | 31.5 (24.0;45.3) | 30.0 (22.0;42.0) | 0.095 (NS) § | 28.0 (21.0;46.0) | 30.0 (22.0;39.0) | 0.791 (NS) § | 30.0 (28.0;35.0) | 33.0 (21.0;42.0) | 0.904 (NS) § |
| Maximum LV thickness (mm) | Median (Q1 ; Q3) | 19.0 (17.0;23.0) | 19.0 (16.0;22.0) | 0.168 (NS) § | 19.0 (17.0;22.0) | 19.9 (16.8;22.5) | 0.947 (NS) § | 19.0 (16.0;24.0) | 20.0 (16.0;24.0) | 0.361 (NS) § |
| LV septal thickness diastole (mm) | Median (Q1 ; Q3) | 18.0 (15.0;22.0) | 18.0 (15.0;21.7) | 0.290 (NS) § | 18.0 (17.0;22.0) | 18.0 (15.0;22.0) | 0.474 (NS) § | 18.0 (15.0;24.0) | 17.0 (15.0;22.0) | 0.930 (NS) § |
| LV posterior wall thickness diastole (mm) | Median (Q1 ; Q3) | 12.0 (10.0;14.0) | 11.0 (9.9;13.0) | 0.005 **(S)** § | 10.1 (9.0;12.0) | 10.0 (9.0;12.0) | 0.534 (NS) § | 12.5 (10.0;14.0) | 11.0 (10.0;13.0) | 0.343 (NS) § |
| Left atrium diameter (mm) | Median (Q1 ; Q3) | 48.0 (42.9;52.0) | 43.0 (38.0;49.0) | <0.001 **(S)** § | 48.0 (43.0;55.0) | 43.0 (37.0;50.0) | <0.001 **(S)** § | 48.0 (41.0;51.0) | 44.0 (37.0;49.0) | 0.018 **(S)** § |
| Left atrial area (cm²) | Median (Q1 ; Q3) | 28.0 (24.8;33.5) | 24.7 (20.0;31.0) | <0.001 **(S)** § | 27.3 (20.5;30.2) | 23.0 (18.5;30.0) | 0.474 (NS) § | 26.5 (22.5;32.6) | 24.0 (20.6;28.0) | 0.287 (NS) § |
| Pattern of LV hypertrophy | No hypertrophy | 8/334 (2.40%) | 28/1172 (2.39%) | 0.973(NS)~ | 1/52 (1.92%) | 3/236 (1.27%) | 0.524(NS)† | 0/31 (0.00%) | 0/75 (0.00%) | 0.323(NS)† |
|  | Asymmetrical septal | 239/334 (71.56%) | 846/1172 (72.18%) |  | 48/52 (92.31%) | 204/236 (86.44%) |  | 18/31 (58.06%) | 53/75 (70.67%) |  |
|  | Concentric | 42/334 (12.57%) | 156/1172 (13.31%) |  | 1/52 (1.92%) | 12/236 (5.08%) |  | 5/31 (16.13%) | 13/75 (17.33%) |  |
|  | Apical | 32/334 (9.58%) | 101/1172 (8.62%) |  | 2/52 (3.85%) | 8/236 (3.39%) |  | 6/31 (19.35%) | 7/75 (9.33%) |  |
| RV dilation |  | 16/347 (4.61%) | 39/1196 (3.26%) | 0.232(NS)~ | 1/53 (1.89%) | 4/237 (1.69%) | 1.000(NS)† | 2/33 (6.06%) | 2/79 (2.53%) | 0.580(NS)† |
| RV global systolic dysfunction |  | 12/347 (3.46%) | 50/1196 (4.18%) | 0.546(NS)~ | 0/53 (0.00%) | 5/237 (2.11%) | 0.589(NS)† | 2/33 (6.06%) | 3/79 (3.80%) | 0.630(NS)† |
| RV hypertrophy |  | 59/347 (17.00%) | 166/1196 (13.88%) | 0.147(NS)~ | 4/53 (7.55%) | 22/237 (9.28%) | 1.000(NS)† | 5/33 (15.15%) | 9/79 (11.39%) | 0.549(NS)† |
| Maximum RV wall thickness (mm) | Median (Q1 ; Q3) | 6.0 (5.0;8.0) | 5.0 (4.0;7.0) | 0.002 **(S)** § | 5.4 (4.0;6.4) | 4.8 (3.5;6.8) | 0.707 (NS) § | 5.0 (5.0;6.0) | 6.0 (4.6;7.0) | 0.730 (NS) § |
| Mitral E-wave (m/s) | Median (Q1 ; Q3) | 0.7 (0.6;0.9) | 0.7 (0.6;0.9) | 0.526 (NS) § | 0.7 (0.5;0.9) | 0.7 (0.6;0.9) | 0.616 (NS) § | 0.7 (0.6;0.9) | 0.8 (0.6;1.0) | 0.467 (NS) § |
| E-wave deceleration time (m/s) | Median (Q1 ; Q3) | 217.5 (183.0;270.0) | 200.0 (160.0;238.0) | <0.001 **(S)** § | 200.0 (170.0;271.0) | 200.5 (170.0;233.5) | 0.641 (NS) § | 212.0 (190.0;270.0) | 210.0 (175.0;280.0) | 0.744 (NS) § |
| Mitral A-wave (m/s) | Median (Q1 ; Q3) | 0.7 (0.5;0.9) | 0.6 (0.5;0.8) | <0.001 **(S)** § | 0.7 (0.5;0.9) | 0.5 (0.4;0.7) | 0.006 **(S)** § | 0.7 (0.6;0.9) | 0.7 (0.5;0.9) | 0.627 (NS) § |
| TDI lateral mitral annulus peak E velocity m/s (E or Ea) (m/s) | Median (Q1 ; Q3) | 0.1 (0.1;0.2) | 0.1 (0.1;0.8) | 0.166 (NS) § | 0.1 (0.1;0.1) | 0.1 (0.1;0.6) | 0.144 (NS) § | 0.1 (0.1;0.1) | 0.1 (0.1;0.1) | 0.793 (NS) § |
| Diastolic dysfunction (grade) | Nomal | 36/266 (13.53%) | 253/941 (26.89%) | <0.001**(S)**~ | 5/39 (12.82%) | 53/175 (30.29%) | 0.028**(S)**~ | 4/19 (21.05%) | 20/65 (30.77%) | 0.186(NS)† |
|  | Grade I (impaired relaxation) | 139/266 (52.26%) | 367/941 (39.00%) |  | 21/39 (53.85%) | 58/175 (33.14%) |  | 12/19 (63.16%) | 23/65 (35.38%) |  |
|  | Grade II (pseudo normal) | 76/266 (28.57%) | 237/941 (25.19%) |  | 11/39 (28.21%) | 42/175 (24.00%) |  | 3/19 (15.79%) | 16/65 (24.62%) |  |
|  | Restrictive | 15/266 (5.64%) | 84/941 (8.93%) |  | 2/39 (5.13%) | 22/175 (12.57%) |  | 0/19 (0.00%) | 6/65 (9.23%) |  |
| Left ventricular outflow tract gradient (mmHg) | Median (Q1 ; Q3) | 13.0 (5.0;46.0) | 9.0 (5.0;33.0) | 0.073 (NS) § | 6.7 (2.0;12.0) | 6.0 (2.0;14.5) | 0.831 (NS) § | 15.0 (8.0;33.0) | 10.0 (5.0;48.0) | 0.569 (NS) § |
| Maximum provoked (by any technique) peak left ventricular outflow tract gradient (mmHg) | Median (Q1 ; Q3) | 23.0 (6.0;64.0) | 12.0 (6.0;45.0) | 0.036 **(S)** § | 10.0 (4.4;30.0) | 8.0 (4.0;22.0) | 0.807 (NS) § | 25.0 (9.0;58.0) | 20.0 (6.0;60.0) | 0.532 (NS) § |
| Aortic regurgitation | None | 258/326 (79.14%) | 857/1128 (75.98%) | 0.712(NS)† | 46/50 (92.00%) | 185/225 (82.22%) | 0.081(NS)~ | 21/32 (65.63%) | 56/74 (75.68%) | 0.196(NS)† |
|  | Mild | 60/326 (18.40%) | 237/1128 (21.01%) |  | 2/50 (4.00%) | 35/225 (15.56%) |  | 10/32 (31.25%) | 11/74 (14.86%) |  |
|  | Moderate | 8/326 (2.45%) | 32/1128 (2.84%) |  | 2/50 (4.00%) | 5/225 (2.22%) |  | 1/32 (3.13%) | 6/74 (8.11%) |  |
|  | Severe | 0/326 (0.00%) | 2/1128 (0.18%) |  | 0/50 (0.00%) | 0/225 (0.00%) |  | 0/32 (0.00%) | 1/74 (1.35%) |  |
| Mitral regurgitation | None | 85/326 (26.07%) | 298/1128 (26.42%) | 0.761(NS)~ | 21/50 (42.00%) | 76/225 (33.78%) | 0.612(NS)† | 12/32 (37.50%) | 27/74 (36.49%) | 0.399(NS)† |
|  | Mild | 185/326 (56.75%) | 636/1128 (56.38%) |  | 22/50 (44.00%) | 119/225 (52.89%) |  | 17/32 (53.13%) | 30/74 (40.54%) |  |
|  | Moderate | 51/326 (15.64%) | 166/1128 (14.72%) |  | 6/50 (12.00%) | 26/225 (11.56%) |  | 3/32 (9.38%) | 15/74 (20.27%) |  |
|  | Severe | 5/326 (1.53%) | 28/1128 (2.48%) |  | 1/50 (2.00%) | 4/225 (1.78%) |  | 0/32 (0.00%) | 2/74 (2.70%) |  |
| Systolic Pulmonary Artery pressure (mmHg) | Median (Q1 ; Q3) | 35.0 (24.0;40.0) | 30.0 (24.0;39.0) | 0.136 (NS) § | 35.0 (20.0;42.0) | 27.5 (21.0;38.5) | 0.583 (NS) § | 39.0 (30.0;47.5) | 37.0 (34.0;45.0) | 0.827 (NS) § |
| **Cardiac Magnetic Resonance Imaging** | | | | | | | | | | |
| Late gadolinium enhancement |  | 82/111 (73.87%) | 320/437 (73.23%) | 0.926(NS)~ | 8/13 (61.54%) | 65/86 (75.58%) | 0.315(NS)† | 7/7 (100.00%) | 11/23 (47.83%) | 0.024**(S)**† |
| LV end-diastolic volume (mL) | Median (Q1 ; Q3) | 133.0 (100.0;175.0) | 136.0 (108.0;166.0) | 0.815 (NS) § | 123.0 (90.0;132.0) | 133.5 (103.5;167.0) | 0.158 (NS) § | 165.0 (132.0;178.0) | 138.5 (111.0;152.5) | 0.174 (NS) § |
| LV end-systolic volume (mL) | Median (Q1 ; Q3) | 37.4 (27.0;57.0) | 43.0 (30.0;59.0) | 0.095 (NS) § | 28.0 (25.0;41.0) | 39.5 (30.0;60.0) | 0.114 (NS) § | 35.0 (34.0;36.0) | 46.5 (29.5;66.0) | 0.324 (NS) § |
| LV ejection fraction (%) | Median (Q1 ; Q3) | 70.5 (62.3;77.1) | 67.1 (60.0;73.1) | 0.021 **(S)** § | 68.3 (64.0;78.8) | 66.1 (60.0;72.2) | 0.526 (NS) § | 79.0 (76.0;80.0) | 67.3 (56.0;73.0) | 0.023 **(S)** § |
| Maximum LV thickness by CMR (mm) | Median (Q1 ; Q3) | 20.0 (17.0;25.0) | 19.0 (16.0;23.0) | 0.018 **(S)** § | 17.0 (15.0;30.0) | 21.0 (17.0;25.0) | 0.378 (NS) § | 21.0 (20.0;31.0) | 19.0 (15.0;22.0) | 0.069 (NS) § |
| Pattern of hypertrophy | Septal | 37/110 (33.64%) | 146/429 (34.03%) | 0.200(NS)~ | 5/13 (38.46%) | 25/86 (29.07%) | 0.300(NS)† | 4/7 (57.14%) | 10/23 (43.48%) | 0.097(NS)† |
|  | Concentric | 41/110 (37.27%) | 183/429 (42.66%) |  | 6/13 (46.15%) | 48/86 (55.81%) |  | 0/7 (0.00%) | 9/23 (39.13%) |  |
|  | Eccentric | 5/110 (4.55%) | 24/429 (5.59%) |  | 1/13 (7.69%) | 1/86 (1.16%) |  | 0/7 (0.00%) | 1/23 (4.35%) |  |
|  | Apical | 7/110 (6.36%) | 31/429 (7.23%) |  | 0/13 (0.00%) | 7/86 (8.14%) |  | 2/7 (28.57%) | 3/23 (13.04%) |  |
|  | Other | 9/110 (8.18%) | 13/429 (3.03%) |  | 0/13 (0.00%) | 3/86 (3.49%) |  | 0/7 (0.00%) | 0/23 (0.00%) |  |
| **Holter** | | | | | | | | | | |
| Non-sustained ventricular tachycardia |  | 56/244 (22.95%) | 169/837 (20.19%) | 0.350(NS)~ | 15/38 (39.47%) | 43/172 (25.00%) | 0.071(NS)~ | 6/22 (27.27%) | 8/51 (15.69%) | 0.332(NS)† |
| Rhythm : atrial fibrillation throughout + paroxysmal atrial fibrillation in sinus rhythm |  | 33/244 (13.52%) | 83/840 (9.88%) | 0.105(NS)~ | 8/38 (21.05%) | 12/172 (6.98%) | 0.013**(S)**† | 1/22 (4.55%) | 7/51 (13.73%) | 0.421(NS)† |
| Rhythm : Conduction defect |  | 5/244 (2.05%) | 8/840 (0.95%) | 0.182(NS)† | 0/38 (0.00%) | 2/172 (1.16%) | 1.000(NS)† | 0/22 (0.00%) | 0/51 (0.00%) | NA |
| **Exercise test** | | | | | | | | | | |
| Absolute workload achieved (Watts)* | Median (Q1 ; Q3) | 100.0 (72.0;142.0) | 120.0 (99.0;167.0) | 0.007 **(S)** § | 100.0 (70.0;150.0) | 119.5 (100.0;177.0) | 0.256 (NS) § | 117.0 (60.0;150.0) | 153.5 (135.0;164.0) | 0.092 (NS) § |
| Absolute workload achieved (METS) | Median (Q1 ; Q3) | 6.0 (5.0;7.5) | 7.8 (6.0;11.0) | <0.001 **(S)** § | 6.0 (5.0;7.5) | 9.7 (6.9;11.5) | 0.020 **(S)** § | 5.4 (5.0;9.7) | 8.2 (6.9;10.8) | 0.221 (NS) § |
| Max VO2 achieved (ml/min/Kg) | Median (Q1 ; Q3) | 17.2 (14.0;20.2) | 21.0 (17.5;27.2) | <0.001 **(S)** § | 17.1 (14.8;23.0) | 24.0 (18.1;29.9) | 0.017 **(S)** § | 14.0 (12.2;29.2) | 22.8 (18.1;24.9) | 0.513 (NS) § |
| % of maximum estimated VO2 achieved Bicycle | Median (Q1 ; Q3) | 29.8 (8.1;33.4) | 28.5 (12.4;34.8) | 0.353 (NS) § | 29.3 (6.8;31.9) | 31.5 (14.8;36.7) | 0.021 **(S)** § | 29.6 (27.0;32.6) | 28.5 (22.2;31.5) | 0.829 (NS) § |
| % of maximum estimated VO2 achieved Treadmill | Median (Q1 ; Q3) | 29.0 (23.6;32.2) | 39.0 (34.0;44.0) | <0.001 **(S)** § | 23.8 (21.2;31.8) | 39.8 (34.0;44.5) | <0.001 **(S)** § | 27.9 (26.4;29.5) | 37.4 (25.8;40.8) | 0.380 (NS) § |
| Ventricular arrhythmia |  | 3/143 (2.10%) | 23/504 (4.56%) | 0.185(NS)~ | 0/24 (0.00%) | 5/110 (4.55%) | 0.585(NS)† | 0/14 (0.00%) | 0/24 (0.00%) | NA |
| Supraventricular arrhythmia |  | 4/143 (2.80%) | 18/504 (3.57%) | 0.798(NS)† | 1/24 (4.17%) | 5/110 (4.55%) | 1.000(NS)† | 1/14 (7.14%) | 1/24 (4.17%) | 1.000(NS)† |
| **Laboratory** | | | | | | | | | | |
| NT-proBNP (pg/mL) | Median (Q1 ; Q3) | 701.5 (321.0;1386.0) | 949.2 (344.9;2128.0) | 0.154 (NS) § | 810.0 (533.0;1466.1) | 858.5 (330.5;2001.0) | 0.993 (NS) § | 661.5 (453.8;3160.0) | 1109.5 (369.0;2565.0) | 0.617 (NS) § |
| BNP (pg/mL) | Median (Q1 ; Q3) | 202.0 (142.0;399.0) | 221.0 (94.5;500.0) | 0.836 (NS) § | 274.5 (120.0;429.0) | 120.0 (70.0;346.0) | 0.573 (NS) § | 155.0 (27.0;237.0) | 202.5 (124.0;530.0) | 0.439 (NS) § |
| **Medications** | | | | | | | | | | |
| Alpha-blockers |  | 17/344 (4.94%) | 31/1096 (2.83%) | 0.057(NS)~ | 2/50 (4.00%) | 3/215 (1.40%) | 0.239(NS)† | 2/34 (5.88%) | 0/74 (0.00%) | 0.097(NS)† |
| Calcium antagonists |  | 82/346 (23.70%) | 156/1100 (14.18%) | <0.001**(S)**~ | 8/50 (16.00%) | 28/216 (12.96%) | 0.572(NS)~ | 12/34 (35.29%) | 7/74 (9.46%) | 0.001**(S)**~ |
| ACE-inhibitors |  | 84/346 (24.28%) | 229/1100 (20.82%) | 0.173(NS)~ | 12/50 (24.00%) | 32/216 (14.81%) | 0.115(NS)~ | 9/34 (26.47%) | 16/74 (21.62%) | 0.579(NS)~ |
| Angiotensin II receptor blockers |  | 77/346 (22.25%) | 163/1100 (14.82%) | 0.001**(S)**~ | 11/50 (22.00%) | 17/216 (7.87%) | 0.003**(S)**~ | 8/34 (23.53%) | 12/74 (16.22%) | 0.364(NS)~ |
| ACE inhibitors/angiotensin II receptor blockers |  | 160/346 (46.24%) | 389/1099 (35.40%) | <0.001**(S)**~ | 23/50 (46.00%) | 49/216 (22.69%) | <0.001**(S)**~ | 17/34 (50.00%) | 28/74 (37.84%) | 0.234(NS)~ |
| Mineralocorticoid receptor antagonists |  | 68/346 (19.65%) | 150/1100 (13.64%) | 0.006**(S)**~ | 11/50 (22.00%) | 33/216 (15.28%) | 0.249(NS)~ | 3/34 (8.82%) | 4/74 (5.41%) | 0.676(NS)† |
| Antiplatelets |  | 108/346 (31.21%) | 280/1100 (25.45%) | 0.035**(S)**~ | 12/50 (24.00%) | 33/216 (15.28%) | 0.138(NS)~ | 10/34 (29.41%) | 25/74 (33.78%) | 0.652(NS)~ |
| Oral anticoagulants |  | 106/346 (30.64%) | 279/1100 (25.36%) | 0.053(NS)~ | 17/50 (34.00%) | 62/216 (28.70%) | 0.460(NS)~ | 7/34 (20.59%) | 16/74 (21.62%) | 0.903(NS)~ |
| Amiodarone |  | 37/313 (11.82%) | 110/1039 (10.59%) | 0.539(NS)~ | 5/46 (10.87%) | 24/205 (11.71%) | 0.872(NS)~ | 2/30 (6.67%) | 8/69 (11.59%) | 0.719(NS)† |
| **Device therapy** | | | | | | | | | | |
| Cardioverter defibrillator implanted |  | 72/360 (20.00%) | 257/1245 (20.64%) | 0.790(NS)~ | 24/54 (44.44%) | 82/246 (33.33%) | 0.122(NS)~ | 7/35 (20.00%) | 20/89 (22.47%) | 0.764(NS)~ |
| Reason for cardioverter defibrillator | Primary prophylaxis | 60/72 (83.33%) | 224/257 (87.16%) | 0.404(NS)~ | 22/24 (91.67%) | 74/82 (90.24%) | 1.000(NS)† | 4/7 (57.14%) | 18/20 (90.00%) | 0.091(NS)† |
|  | Secondary prophylaxis | 12/72 (16.67%) | 33/257 (12.84%) |  | 2/24 (8.33%) | 8/82 (9.76%) |  | 3/7 (42.86%) | 2/20 (10.00%) |  |
| Pacemaker implanted |  | 32/356 (8.99%) | 92/1235 (7.45%) | 0.340(NS)~ | 3/51 (5.88%) | 18/245 (7.35%) | 1.000(NS)† | 6/35 (17.14%) | 9/89 (10.11%) | 0.359(NS)† |
| Reason for pacemaker: Brady |  | 17/35 (48.57%) | 63/97 (64.95%) | 0.089(NS)~ | 5/6 (83.33%) | 8/18 (44.44%) | 0.166(NS)† | 3/6 (50.00%) | 7/9 (77.78%) | 0.329(NS)† |
| Reason for pacemaker: Treatment of left ventricular outflow tract obstruction |  | 8/33 (24.24%) | 15/95 (15.79%) | 0.276(NS)~ | 1/3 (33.33%) | 3/19 (15.79%) | 0.470(NS)† | 1/6 (16.67%) | 0/9 (0.00%) | 0.400(NS)† |
| Reason for pacemaker: Cardiac resynchronization therapy |  | 5/34 (14.71%) | 11/96 (11.46%) | 0.762(NS)† | 2/4 (50.00%) | 3/18 (16.67%) | 0.210(NS)† | 1/6 (16.67%) | 0/9 (0.00%) | 0.400(NS)† |
| **Other procedures** | | | | | | | | | | |
| Septal myectomy |  | 20/360 (5.56%) | 61/1245 (4.90%) | 0.617(NS)~ | 3/54 (5.56%) | 16/246 (6.50%) | 1.000(NS)† | 1/35 (2.86%) | 2/89 (2.25%) | 1.000(NS)† |
| Alcohol septal ablation |  | 18/360 (5.00%) | 44/1245 (3.53%) | 0.204(NS)~ | 1/54 (1.85%) | 6/246 (2.44%) | 1.000(NS)† | 2/35 (5.71%) | 3/89 (3.37%) | 0.620(NS)† |

**Legend:** § : Kruskal-Wallis test ; † : Exact-Fisher test ; ~ : Chi-square test ; NC : Not calculable. All continuous variables are presented as Median (Q1 ; Q3) and categorical variables as N and percentage. HCM : hypertrophic cardiomyopathy ; SCD : sudden cardiac death; NYHA : New York Heart Association ; BBB : bundle branch block ; LBBB : left bundle branch block ; LVEDD : left ventricular end-diastolic dimension ; LVESD : left ventricular end-systolic dimension ; LV : left ventricle ; RV : right ventricle ; VO2 : oxygen consumption.

#

# **Supplemental Table 4. Univariate logistic regression analysis of different variables associated with atrial fibrillation**

| **Variable** |  | **Number of patients with Atrial Fibrillation (N=478)** | **Number of patients with no Atrial Fibrillation (N=1221)** | **OR [95% CI]** | **OR p-value** |
| --- | --- | --- | --- | --- | --- |
| Hypertension | No | 268/478 (56.07%) | 799/1221 (65.44%) | / | / |
|  | Yes | 210/478 (43.93%) | 422/1221 (34.56%) | 1.484 [1.196-1.841] | <0.001 (S) |
| Diabetes mellitus | No diabetes | 405/478 (84.73%) | 1120/1221 (91.73%) | / | / |
|  | Diabetes | 73/478 (15.27%) | 101/1221 (8.27%) | 1.999 [1.449-2.759] | <0.001 (S) |
| Obesity | Not obese | 323/478 (67.57%) | 909/1221 (74.45%) | / | / |
|  | Obese | 113/478 (23.64%) | 243/1221 (19.90%) | 1.309 [1.012-1.692] | 0.040 (S) |
| Age at enrolment |  | 478/478 (mean=60.0) | 1221/1221 (mean=50.9) | 1.041 [1.033-1.049] | <0.001 (S) |
| Males | Male | 271/478 (56.69%) | 730/1221 (59.79%) | / | / |
|  | Female | 207/478 (43.31%) | 491/1221 (40.21%) | 1.136 [0.917-1.407] | 0.244 (NS) |
| Genotype | Genotype-negative | 36/478 (7.53%) | 95/1221 (7.78%) | / | / |
|  | Genotype-positive | 92/478 (19.25%) | 223/1221 (18.26%) | 1.089 [0.691-1.714] | 0.714 (NS) |
| Maximum left ventricular wall thickness (mm) |  | 431/478 (mean=19.4) | 1117/1221 (mean=19.8) | 0.985 [0.963-1.008] | 0.207 (NS) |
| Maximum provoked (by any technique) peak left ventricular outflow tract gradient (mmHg) |  | 133/478 (mean=29.5) | 388/1221 (mean=33.6) | 0.997 [0.991-1.002] | 0.269 (NS) |
| Left atrium diameter (mm) |  | 412/478 (mean=49.2) | 1020/1221 (mean=42.6) | 1.103 [1.086-1.121] | <0.001 (S) |
| Proband or relative | Relative | 41/478 (8.58%) | 232/1221 (19.00%) | / | / |
|  | Proband | 314/478 (65.69%) | 714/1221 (58.48%) | 2.488 [1.740-3.558] | <0.001 (S) |
| Age at diagnosis (years) |  | 472/478 (mean=49.7) | 1201/1221 (mean=43.7) | 1.019 [1.013-1.025] | <0.001 (S) |
| Physical activity : intensily | No | 376/478 (78.66%) | 925/1221 (75.76%) | / | / |
|  | Yes | 3/478 (0.63%) | 34/1221 (2.78%) | 0.217 [0.066-0.711] | 0.012 (S) |

# **Supplemental Table 5. Multivariate logistic regression analysis of different variables associated with atrial fibrillation**

| **Sample size** | **Variable** | **OR [95% CI]** | **OR p-value** |
| --- | --- | --- | --- |
| 1414/1699 | Age at enrolment | 1.058 [1.042-1.075] | <0.001 (S) |
|  | Left atrium diameter (mm) | 1.097 [1.079-1.116] | <0.001 (S) |
|  | Age at diagnosis (years) | 0.981 [0.969-0.994] | 0.003 (S) |

# **Supplemental Table 6. Univariate regression analysis of different variables associated with maximum left ventricular wall thickness**

| **Variable** |  | **Estimation of parameters** | **Global P-value** |
| --- | --- | --- | --- |
| Age at diagnosis (years) |  | -0.043 [-0.056;-0.030] | <0.001 (S) |
| Age at enrollment |  | -0.049 [-0.064;-0.033] | <0.001 (S) |
| Atrial Fibrillation | Yes | -0.352 [-0.899;0.195] | 0.207 (NS) |
|  | No | / |  |
| Diabetes mellitus | Diabetes | -0.521 [-1.322;0.279] | 0.202 (NS) |
|  | No diabetes | / |  |
| Genotype | Genotype-positive | -0.283 [-1.477;0.912] | 0.642 (NS) |
|  | Genotype-negative | / |  |
| Hypertension | Yes | -0.491 [-0.995;0.014] | 0.057 (S) |
|  | No | / |  |
| Left atrium diameter (mm) |  | 0.085 [0.055;0.115] | <0.001 (S) |
| Maximum provoked (by any technique) peak left ventricular outflow tract gradient (mmHg) |  | 0.016 [0.005;0.027] | 0.005 (S) |
| Obesity | Obese | 0.264 [-0.341;0.869] | 0.392 (NS) |
|  | Not obese | / |  |
| Physical activity : intensily | Yes | 0.755 [-0.936;2.447] | 0.381 (NS) |
|  | No | / |  |
| Proband or relative | Proband | 1.132 [0.461;1.803] | <0.001 (S) |
|  | Relative | / |  |
| Sex | Female | -0.304 [-0.801;0.192] | 0.229 (NS) |
|  | Male | / |  |

# **Supplemental Table 7. Multivariate regression analysis of different variables associated with maximum left ventricular wall thickness**

| **Variable** | **Estimation of parameters** | **Global P-value** |
| --- | --- | --- |
| Age at diagnosis (years) | -0.009 [-0.061;0.042] | 0.716 (NS) |
| Age at enrollment | -0.052 [-0.107;0.004] | 0.066 (NS) |
| Hypertension | 0.078 [-0.931;1.086] | 0.880 (NS) |
|  | / |  |
| Left atrium diameter (mm) | 0.066 [0.012;0.119] | 0.017 (S) |
| Maximum provoked (by any technique) peak left ventricular outflow tract gradient (mmHg) | 0.018 [0.006;0.030] | 0.004 (S) |
| Proband or relative | 1.218 [-0.167;2.603] | 0.085 (NS) |
|  | / |  |

# **Supplemental Table 8. Univariate regression analysis of different variables associated with left ventricular ejection fraction**

| **Variable** |  | **Estimation of parameters** | **Global P-value** |
| --- | --- | --- | --- |
| Age at diagnosis (years) |  | 0.001 [-0.031;0.033] | 0.944 (NS) |
| Age at enrollment |  | -0.046 [-0.082;-0.010] | 0.013 (S) |
| Atrial Fibrillation | Yes | -4.535 [-5.808;-3.262] | <0.001 (S) |
|  | No | / |  |
| Diabetes mellitus | Diabetes | 0.305 [-1.575;2.185] | 0.750 (NS) |
|  | No diabetes | / |  |
| Genotype | Genotype-positive | 0.007 [-2.443;2.457] | 0.995 (NS) |
|  | Genotype-negative | / |  |
| Hypertension | Yes | -0.738 [-1.943;0.466] | 0.230 (NS) |
|  | No | / |  |
| Left atrium diameter (mm) |  | -0.129 [-0.198;-0.060] | <0.001 (S) |
| Maximum left ventricular wall thickness (mm) |  | 0.240 [0.123;0.357] | <0.001 (S) |
| Maximum provoked (by any technique) peak left ventricular outflow tract gradient (mmHg) |  | 0.062 [0.039;0.086] | <0.001 (S) |
| Obesity | Obese | 0.372 [-1.080;1.825] | 0.615 (NS) |
|  | Not obese | / |  |
| Physical activity : intensily | Yes | 3.518 [-0.620;7.655] | 0.096 (S) |
|  | No | / |  |
| Proband or relative | Proband | -1.263 [-2.805;0.279] | 0.108 (S) |
|  | Relative | / |  |
| Sex | Female | 1.059 [-0.125;2.242] | 0.080 (S) |
|  | Male | / |  |

# **Supplemental Table 9. Multivariate regression analysis of different variables associated with left ventricular ejection fraction**

| **Variable** | **Modality** | **comparison** | **Estimation of parameters** | **P-value** | **Global P-value** |
| --- | --- | --- | --- | --- | --- |
| Age at enrollment |  |  | 0.029 [-0.040;0.098] | 0.412 (NS) | 0.412 (NS) |
| Atrial Fibrillation | Yes | Yes vs No | -4.389 [-6.833;-1.945] | <0.001 (S) | <0.001 (S) |
|  | No | Ref# | / | / |  |
| Left atrium diameter (mm) |  |  | -0.018 [-0.150;0.114] | 0.787 (NS) | 0.787 (NS) |
| Maximum left ventricular wall thickness (mm) |  |  | 0.239 [0.025;0.453] | 0.029 (S) | 0.029 (S) |
| Maximum provoked (by any technique) peak left ventricular outflow tract gradient (mmHg) |  |  | 0.061 [0.034;0.088] | <0.001 (S) | <0.001 (S) |
| Physical activity : intensily | Yes | Yes vs No | 3.602 [-1.952;9.156] | 0.203 (NS) | 0.203 (NS) |
|  | No | Ref# | / | / |  |
| Proband or relative | Proband | Proband vs Relative | -0.580 [-3.708;2.549] | 0.716 (NS) | 0.716 (NS) |
|  | Relative | Ref# | / | / |  |
| Sex | Female | Female vs Male | 0.862 [-1.289;3.013] | 0.431 (NS) | 0.431 (NS) |
|  | Male | Ref# | / | / |  |
