# Supplementary material for: Association between common cardiovascular risk factors and clinical phenotype in patients with hypertrophic cardiomyopathy from the European Society of Cardiology (ESC) EurObservational Research Programme (EORP) Cardiomyopathy/Myocarditis registry
Source: Eur Heart J Qual Care Clin Outcomes. 2022 Feb 9;9(1):42–53. doi: 10.1093/ehjqcco/qcac006 (PMC9745665; doi:10.1093/ehjqcco/qcac006)
Supplement: qcac006_Supplemental_Files [file qcac006_supplemental_files.zip › appendix 1.docx]

##

## Appendix 1

## EORP Oversight Committee

Christopher Peter Gale, Chair, GB, Branko Beleslin, RS, Andrzej Budaj, PL, Ovidiu Chioncel, RO, Nikolaos Dagres, DE, Nicolas Danchin, FR, David Erlinge, SE, Jonathan Emberson, GB,

Michael Glikson, IL, Alastair Gray, GB, Meral Kayikcioglu, TR, Aldo Maggioni, IT, Klaudia

Vivien Nagy, HU, Aleksandr Nedoshivin, RU, Anna-Sonia Petronio, IT, Jolien Roo Hesselink, NL, Lars Wallentin, SE, Uwe Zeymer, DE.

## Executive Committee

Alida Caforio (Chair), IT, Juan Ramon Gimeno Blanes, ES, Philippe Charron, FR, Perry Elliott, GB, Juan Pablo Kaski, GB, Aldo P. Maggioni, IT, Luigi Tavazzi, IT, Michal Tendera, PL

## Investigators

**Belarus:** *Minsk:* S. Komissarova, N. Chakova, S. Niyazova, **Czech Republic:** *Prague:*A. Linhart, P. Kuchynka, T. Palecek, J. Podzimkova, M. Fikrle, E. Nemecek, **Denmark:** *Copenhagen:* H. Bundgaard, J. Tfelt-Hansen, J. Theilade, J.J. Thune, A. Axelsson, *Odense:* J. Mogensen, F. Henriksen, T. Hey, S.K. Nielsen, L. Videbaek, S. Andreasen, H. Arnsted, **Egypt:** *Zagazig:* A. Saad, M. Ali, **Finland:** *Helsinki:* J. Lommi, T. Helio, M.S. Nieminen, **France:** *Boulogne-Billancourt:* O. Dubourg, N. Mansencal, M. Arslan, V. Siam Tsieu, *Créteil:* T. Damy, A. Guellich, S. Guendouz, C.M. Tissot, A. Lamine, S. Rappeneau, *Paris:* A. Hagege, M. Desnos, A. Bachet, M. Hamzaoui, *Paris:* P. Charron, R. Isnard, L. Legrand, C. Maupain, E. Gandjbakhch, M. Kerneis, J-F. Pruny, **Germany:** *Crailsheim:* A. Bauer, B. Pfeiffer, *Greifswald:* S.B. Felix, M. Dorr, S. Kaczmarek, K. Lehnert, A-L. Pedersen, D. Beug, M. Bruder, *Homburg/Saar:* M. Böhm, I. Kindermann, Y. Linicus, C. Werner, B. Neurath, M. Schild-Ungerbuehler, *Schweinfurt:* H. Seggewiss, B. Pfeiffer, A. Neugebauer, **Great Britain:** *Belfast:* P. McKeown, A. Muir, J. McOsker, T. Jardine, G. Divine, *London:* P. Elliott, M. Lorenzini, O. Watkinson, E. Wicks, *London:* H. Iqbal, S. Mohiddin, C. O'Mahony, N. Sekri, *London:* G. Carr-White, T. Bueser, R. Rajani, L. Clack, J. Damm, S. Jones, R. Sanchez-Vidal, M. Smith, T. Walters, K. Wilson, *London:* S. Rosmini, **Greece:** *Athens:* A. Anastasakis, K. Ritsatos, V. Vlagkouli, **Hungary:** *Szeged:* T. Forster, R. Sepp, J. Borbas, V. Nagy, A. Tringer, K. Kakonyi, L.A. Szabo, **Iran:** *Tehran:* M. Maleki, F. Noohi Bezanjani, A. Amin, N. Naderi, M. Parsaee, S. Taghavi, B. Ghadrdoost, S. Jafari, M. Khoshavi, **Italy:** *Bologna:* C. Rapezzi, E. Biagini, A. Corsini, C. Gagliardi, M. Graziosi, S. Longhi, A. Milandri, L. Ragni, S. Palmieri, *Florence:* I. Olivotto, A. Arretini, G. Castelli, F. Cecchi, A. Fornaro, B. Tomberli, *Genoa:* P. Spirito, E. Devoto, *Milan:* P. Della Bella, G. Maccabelli, S. Sala, F. Guarracini, G. Peretto, *Naples:* M.G. Russo, R. Calabro, G. Pacileo, G. Limongelli, D. Masarone, V. Pazzanese, A. Rea, M. Rubino, S. Tramonte, F. Valente, M. Caiazza, A. Cirillo, G. Del Giorno, A. Esposito, R. Gravino, T. Marrazzo, *Naples:* B. Trimarco, M-A. Losi, C. Di Nardo, A. Giamundo, F. Musella, F. Pacelli, A. Scatteia, G. Canciello, *Padua:* A. Caforio, S. Iliceto, C. Calore, L. Leoni, M. Perazzolo Marra, I. Rigato, G. Tarantini, A. Schiavo, M. Testolina, *Pavia:* E. Arbustini, A. Di Toro, L.P. Giuliani, A. Serio . *Rome:* F. Fedele, A. Frustaci, M. Alfarano, C. Chimenti, *Rome:* F. Drago, A. Baban, *Rome:* L. Calò, C. Lanzillo, A. Martino, *Rome:* M. Uguccioni, E. Zachara, G. Halasz, F. Re, *Trieste:* G. Sinagra, C. Carriere, M. Merlo, F. Ramani, **Lithuania:** *Kaunas:* A. Kavoliuniene, A. Krivickiene, E. Tamuleviciute-Prasciene, M. Viezelis, *Vilnius:* J. Celutkiene, L. Balkeviciene, M. Laukyte, E. Paleviciute, **Netherlands:** *Amsterdam:* Y. Pinto, A. Wilde, *Utrecht:* F.W. Asselbergs, A. Sammani, J. Van Der Heijden, L. Van Laake, N. De Jonge, R. Hassink, J.H. Kirkels, **Nigeria:** *Lagos:* J. Ajuluchukwu, A. Olusegun-Joseph, E. Ekure, **Poland:** *Katowice:* K. Mizia-Stec, M. Tendera, A. Czekaj, A. Sikora-Puz, A. Skoczynska, M. Wybraniec, *Krakow:* P. Rubis, E. Dziewiecka, S. Wisniowska-Smialek, *Warsaw:* Z. Bilinska, P. Chmielewski, B. Foss- Nieradko, E. Michalak, M. Stepien-Wojno, B. Mazek, **Portugal:** *Almada:* L. Rocha Lopes, A.R. Almeida, I. Cruz, A.C. Gomes, A.R. Pereira, *Lisbon:* D. Brito, H. Madeira, A.R. Francisco, M. Menezes, O. Moldovan, T. Oliveira Guimaraes, D. Silva, **Romania:** *Bucharest:* C. Ginghina, R. Jurcut, A. Mursa, B.A. Popescu, E. Apetrei, S. Militaru, I. Mircea Coman, *Targu-Mures:* A. Frigy, Z. Fogarasi, I. Kocsis, I.A. Szabo, L. Fehervari, **Russian Federation:** *Moscow:* I. Nikitin, E. Resnik, M. Komissarova, V. Lazarev, M. Shebzukhova, D. Ustyuzhanin, *Moscow:* O. Blagova, I. Alieva, V. Kulikova, Y. Lutokhina, E. Pavlenko, N. Varionchik, **Serbia:** *Belgrade:*  A.D. Ristic, P.M. Seferovic, I. Veljic, I. Zivkovic, I. Milinkovic, A. Pavlovic, G. Radovanovic, D. Simeunovic, *Belgrade:* M. Zdravkovic, M. Aleksic, J. Djokic, S. Hinic, S. Klasnja, K. Mircetic, **Spain:** *A Coruna:* L. Monserrat, X. Fernandez, D. Garcia-Giustiniani, J.M. Larrañaga, M. Ortiz-Genga, R. Barriales-Villa, C. Martinez-Veira, E. Veira, *Barcelona:* A. Cequier, J. Salazar-Mendiguchia, N. Manito, J. Gonzalez, *Madrid:* F. Fernández-Avilés, C. Medrano, R. Yotti, S. Cuenca, M.A. Espinosa, I. Mendez, E. Zatarain, R. Alvarez, *Madrid:* P. Garcia Pavia, A. Briceno, M. Cobo-Marcos, F. Dominguez, *Malaga:* E. De Teresa Galvan, J.M. García Pinilla, N. Abdeselam-Mohamed, M.A. Lopez-Garrido, L. Morcillo Hidalgo, M.V. Ortega-Jimenez, A. Robles Mezcua, A. Guijarro-Contreras, D. Gomez-Garcia, M. Robles-Mezcua, *Murcia:* J.R. Gimeno Blanes, F.J. Castro, C. Munoz Esparza, M. Sabater Molina, M. Sorli García, D. Lopez Cuenca, *Palma de Mallorca:* T. Ripoll-Vera, J. Alvarez, J. Nunez, Y. Gomez, *Salamanca:* P.L. Sanchez Fernandez, E. Villacorta, C. Avila, L. Bravo, E. Diaz-Pelaez, M. Gallego-Delgado, L. Garcia-Cuenllas, B. Plata, *Seville:* J.E. Lopez-Haldon, M.L. Pena Pena, E.M. Cantero Perez, *Valencia:* E. Zorio, M.A. Arnau, J. Sanz, E. Marques-Sule.
