# Supplementary figures and images for: Association between common cardiovascular risk factors and clinical phenotype in patients with hypertrophic cardiomyopathy from the European Society of Cardiology (ESC) EurObservational Research Programme (EORP) Cardiomyopathy/Myocarditis registry
Source: Eur Heart J Qual Care Clin Outcomes. 2022 Feb 9;9(1):42–53. doi: 10.1093/ehjqcco/qcac006 (PMC9745665; doi:10.1093/ehjqcco/qcac006)

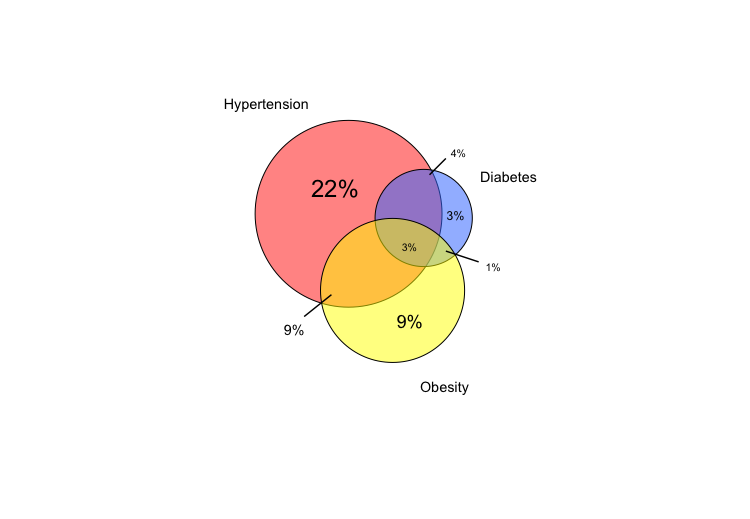

Supplement: qcac006_Supplemental_Files [file qcac006_supplemental_files.zip › Supplemental figure.tiff]
